# Histone Acetylation Differentially Modulates CTCF-CTCF Loops and Intra-TAD Interactions

**DOI:** 10.1038/s41467-026-75818-8

**Published:** 2026-07-20

**Authors:** Rebecca G. Smith, Yu Fu, Kathleen L. Schiela, Madison Dautle, Ryan A. Williams, Hannah M. Wilson, Chloe Azadegan, Johnathan R. Whetstine, Job Dekker, Yu Liu

**Affiliations:** 1 Cancer Epigenetics Institute, Fox Chase Cancer Center, Philadelphia, PA USA; 2 Nuclear Dynamics and Cancer Program, Fox Chase Cancer Center, Philadelphia, PA USA; 3 Institute for Cancer Research, Fox Chase Cancer Center, Philadelphia, PA USA; 4 Department of Biology, Temple University, Philadelphia, PA USA; 5 Department of Systems Biology, University of Massachusetts Chan Medical School, Worcester, MA USA; 6 Biostatitics and Bioinformatics Facility, Fox Chase Cancer Center, Philadelphia, PA USA; 7 Program in Molecular and Cellular Biology and Genetics, Graduate School of Biomedical Sciences and Professional Studies, Drexel University College of Medicine, Philadelphia, PA USA; 8 Howard Hughes Medical Institute, Chevy Chase, MD USA

**Keywords:** Genome, Epigenomics

## Abstract

The cohesin complex structures the interphase genome of human cells by extruding loops and organizing topologically associating domains (TADs), yet how chromatin state regulates cohesin-chromatin interactions remains unclear. Here, we show that histone hyperacetylation induced by trichostatin A (TSA) selectively disrupts short-range intra-TAD interactions while largely preserving CTCF-anchored loops. These distinct responses define two functional cohesin populations: a TSA-sensitive pool associated with dynamic loop extrusion, and a TSA-resistant pool at CTCF sites maintained by topological entrapment. Using a semi-in vitro system with TEV-cleavable RAD21, we demonstrate that hyperacetylation increases the sensitivity of CTCF-anchored loops to cohesin ring cleavage, supporting a topological basis for their stability. We further identify a TSA-sensitive cohesin fraction at CTCF sites, suggesting transient, non-encircling intermediates. Together, our results reveal that cohesin exists in distinct biochemical states that differentially regulate chromatin loop stability and responsiveness to epigenomic perturbation.

## Abstract (web summary)

Here the authors show that histone hyperacetylation selectively disrupts dynamic chromatin loops while preserving CTCF-anchored loops, revealing distinct cohesin states that balance genome flexibility and structural stability.

## Introduction

The cohesin complex plays a critical role in the formation of chromatin loops and domains^1,2^ and thereby contributes to the three-dimensional (3D) organization of the genome. Cohesin is composed of three core subunits: SMC1, SMC3 and RAD21, which together form a ring structure^3–7^. This ring structure allows cohesin to mediate cohesion of sister chromatids during mitosis^7–9^ by topologically encircling two sister chromatids. During interphase, cohesin actively extrudes chromatin loops that bring distant genomic regions into proximity^10–12^. CTCF-bound sites block cohesin-mediated loop extrusion in an orientation-dependent manner, leading to stalling of cohesin complexes at these sites. These stalled cohesin complexes produce chromosome structures that are visible as dots of enriched interactions on Hi-C contact maps. In contrast, the actively extruding cohesin mediates transient interactions, not reproducibly positioned at specific positions. Over the population, the sum of these dynamic loops produces enriched interactions within topologically associating domains (TADs)^2,13–17^.

Recent studies have shown that the stalled cohesin at pairs of convergent CTCF sites entraps chromatin in a similar way as cohesive cohesin holds pairs of sister chromatids during mitosis, i.e., chromatin going through the ring^18,19^. Proteolytic cleavage of RAD21 leads to opening of the cohesin ring, cohesin dissociation from CTCF-bound sites, and loss of CTCF-CTCF loops. In contrast, actively extruding cohesin complexes that are not stalled at CTCF sites and that are positioned within TADs engage chromatin in a different way (chromatin doesn’t go through the cohesin ring). These extruding complexes “clamp” the chromatin template^20,21^, remain bound to chromatin after proteolytic cleavage of RAD21, and their loops remain in place. These complexes can be dissociated from DNA by high salt concentrations even without cleavage of RAD21 indicating they do not encircle the DNA. These findings indicate cohesin engages with chromatin in different ways depending on its location within the genome. However, the precise mechanisms by which cohesin interacts with chromatin in these varied contexts, and how these interactions are modulated by chromatin modifications, remain incompletely understood.

One such chromatin modification is histone acetylation, which is known to reduce nucleosome interactions and promote a more open chromatin conformation^22^. Histone hyperacetylation, e.g., induced in the presence of HDAC inhibitors like trichostatin A (TSA), leads to global chromatin decompaction^23–25^. While the effects of histone hyperacetylation on chromatin accessibility and gene expression have been well documented, its impact on cohesin-mediated chromatin interactions is less clear.

In this study, we demonstrated that TSA-induced hyperacetylated histones have minimal impact on long-range chromatin interactions (>2 Mb) but reduce short-range (<2 Mb) interactions, due in part to loss of cohesin loops within TADs. While CTCF-CTCF loops are not affected by hyperacetylation when the cohesin ring is intact, they are lost to a greater extent after RAD21 cleavage. These findings suggest that histone acetylation lowers the affinity of the actively extruding clamped cohesin complexes within TADs, while cohesin complexes at the bases of CTCF-CTCF loops remain bound as long as they can encircle the two looping anchors. Most notably, our results suggest the presence of two distinct populations of cohesin that can co-exist at CTCF-CTCF sites: one that pseudotopologically encircles chromatin and is resistant to histone hyperacetylation (TSA-resistant), and another that does not encircle chromatin and is sensitive to histone hyperacetylation (TSA-sensitive), resembling the cohesin found within TADs. Together, these findings provide mechanistic insight into the context-dependent behavior of cohesin and underscore the differential impact of histone acetylation on 3D genome organization.

## Results

### Impact of TSA-induced histone hyperacetylation on genome compartmentalization

TSA is widely recognized for its ability to increase histone acetylation and promote chromatin decompaction^23,24,26^. While many studies employ overnight TSA treatments, such prolonged exposure often leads to extensive changes in the transcriptome, epigenome, and nuclear morphology, complicating the interpretation of results. To minimize these confounding effects, we treated cells with 500 nM TSA for 3 hours, as supported by recent studies^27,28^. Western blot analysis confirmed that histone acetylation levels peaked under these conditions (Supplementary Fig. 1a, b). Furthermore, cell cycle analysis indicated that this treatment did not perturb cell cycle progression (Supplementary Fig. 1c). Thus, we adopted the 3-hour 500 nM TSA treatment regimen for all subsequent experiments.

**Fig. 1:**
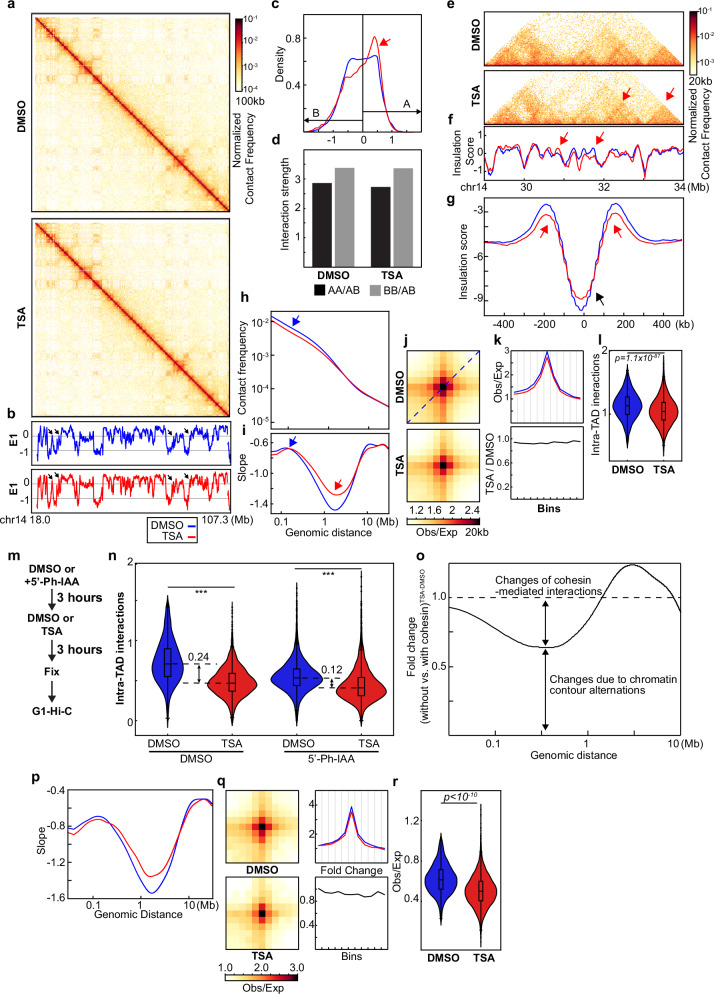
Histone hyperacetylation alters genome interactions. **a** Hi-C interaction maps for HAP1 cells treated with DMSO or TSA (chromosome 14: 18.0-107.3 Mb). **b** Eigenvector (E1) profiles across the same region; arrows indicate loci with increased E1 values. **c** Distribution of E1 values, showing increased A-compartment signals following TSA treatment. **d** Compartment interaction strength (A-A and B-B; see Methods). **e** Hi-C maps for chromosome 14: 29-34 Mb. **f** Insulation profiles for the same region. **g** Aggregate Hi-C signal at TAD boundaries identified in DMSO-treated cells. *P(s)* plots (**h**) and derivatives (**i**), with arrows indicating cohesin loop signatures. **j** Aggregate Hi-C signal at 8334 loops^12^. **k** Loop strength quantified along the loop-line (diagonal from bottom-left to top-right); blue and red indicate DMSO and TSA, respectively. The bottom panel shows the TSA/DMSO ratio. **l** Average intra-TAD interactions, significantly reduced upon TSA treatment (one replicate with DMSO and TSA conditions, two-tailed Wilcoxon rank-sum test, *p* = 1.1 × 10^−87^). Effect of cohesin depletion on TSA-induced intra-TAD interaction changes. RAD21 degron cells were treated with 5′-Ph-IAA to deplete cohesin prior to TSA or DMSO treatment, followed by Hi-C analysis (**m**). Short-range intra-TAD interactions were quantified (**n**); significance was assessed using two-tailed Wilcoxon rank-sum tests (***p < 1 × 10^−10^). **o** Fold change of TSA-induced *P(s)* alterations with or without cohesin. **p-r**, ChIP-loop analysis using RAD21 antibody. **p** Derivative plots showing global cohesin loop signals. **q** CTCF-CTCF loop pileup and corresponding loop-line plots. **r** Average intra-TAD interactions, with significance assessed by two-tailed Wilcoxon rank-sum test (see Methods). All boxplots were generated using the R function boxplot() with default settings: boxes represent the interquartile range (25th−75th percentiles), the center line indicates the median, and whiskers extend to data within 1.5× the interquartile range. All data shown are from one of two independent replicates; the second replicate is provided in Supplementary Fig. 1. Source data are provided.

We next performed Hi-C analysis on HAP1-RAD21^TEV^ cells^18^ following TSA treatment. Hi-C contact maps revealed that TSA treatment increases the E1 eigenvector values at some loci (dark arrows, Fig. 1a, b; Supplementary Fig. 1d, e). The E1 eigenvector, derived from principal component analysis of the Hi-C interaction matrix, is a widely used metric to delineate A and B compartment positions^29^.

We next analyzed the distribution of E1 values and found that TSA-induced histone hyperacetylation increased the number of A compartment bins, consistent with observed elevation in E1 values and indicative of some changes in compartment patterns (Fig. 1c; Supplementary Fig. 1f). We calculated compartment strength, i.e., the strength of the preference of interactions between loci of the same compartment type, by ranking loci by their E1 value and plotting pairwise interactions normalized for their expected interaction frequency given their genomic distance. Compartment strength is then calculated as the ratio of A-A or B-B to A-B and B-A interactions (see Methods)^15^. Histone acetylation is known to promote chromatin decompaction, which could potentially influence compartment interactions. However, we observed minimal changes in compartmentalization strength in TSA-treated cells (Fig. 1d; Supplementary Fig. 1g). In summary, TSA treatment alters compartment patterns by increasing the number of A compartment bins but has minimal impact on overall compartment interaction strength.

### TSA-induced histone acetylation reduces local chromatin interactions

To investigate the local effects of TSA treatment on chromatin organization, we first analyzed Hi-C contact maps. TSA-treated cells displayed reduced interactions within chromatin domains (red arrows, Fig. 1e; Supplementary Fig. 1h). This can be quantified directly by plotting Hi-C interaction frequency (*P*) as a function of genomic distance (*s*) (Fig. 1h and Supplementary Fig. 1k). In TSA treated cells, interaction frequencies for loci separated by up to 2 Mb are reduced about 20%. At this length scale cohesin-mediated loops and TADs are dominant chromatin folding features. We therefore examined these features in more detail by calculating insulation profiles^30^ (Fig. 1f; Supplementary Fig. 1i) and observed a notable reduction in insulation scores at domain boundaries following TSA treatment. We next assessed TAD boundary insulation strength by aggregating interactions at and around boundaries. In DMSO-treated cells, the characteristic depletion of interactions across boundaries was evident, as shown by the depth of the valley in the insulation profile (blue line, Fig. 1g; Supplementary Fig. 1j), which reflects the strength of boundary insulation between adjacent TADs. In TSA-treated cells, the valley depth was reduced (red line, Fig. 1g; Supplementary Fig. 1j), indicating weakened TAD boundary insulation. This reduction likely stems from decreased interactions within flanking TADs, as reflected by the diminished shoulders of the valley (red arrows, Fig. 1g and Supplementary Fig. 1j). In summary, TSA treatment reduces local chromatin interactions and weakens TADs.

### Histone hyperacetylation reduces cohesin-mediated loops

The shape of *P(s)* reflects key features of chromatin organization, including cohesin-mediated loops^31,32^. In DMSO-treated cells, *P(s)* displayed a characteristic shoulder where interactions decayed more slowly for loci separated by ~100 kb (Fig. 1h; Supplementary Fig. 1k, blue arrow). The derivative of *P(s)* highlighted this feature as a local peak, corresponding to the average loop size, while the valley depth at ~2 Mb reflected loop densities (Fig. 1i and Supplementary Fig. 1l)^31,33^.

For TSA-treated cells, the derivative of *P(s)* showed two changes compared to controls: first, the local peak reflecting average loop size was slightly shifted to the right; and second, the depth of the valley at ~2 Mb was reduced (Fig. 1i; Supplementary Fig. 1l, red arrow). Together, this indicates a reduction of the number of cohesin-mediated loops, and coincident extension of their size. Cohesin mediates two major types of chromatin loops: loops within TADs generated by actively extruding cohesin and CTCF-CTCF loops stabilized by cohesin stalling at CTCF sites. The derivative plots represent signals from both types of cohesin-mediated loops^1,2,18,31,33^. The reduced peak depth at ~2 Mb in TSA-treated cells suggests a loss of loops, but does not provide information about which loops are lost. To determine whether loops generated by actively extruding cohesin, by cohesin stalled at pairs of CTCF sites, or by both mechanisms are affected, we next examined the impact on each loop type individually.

We first investigated the impact of TSA treatment on CTCF-CTCF looping interactions. In DMSO-treated cells, strong focal enrichment of interactions at loop anchors was readily observed, and this enrichment remained largely unchanged in TSA-treated cells (Fig. 1j and Supplementary Fig. 1m). To quantify loop strength, we plotted Hi-C interaction frequencies along the diagonal of loop aggregation heatmaps (Fig. 1j and Supplementary Fig. 1m, blue dashed line, top heatmap). These ‘loop-line’ plots revealed peaks corresponding to CTCF-CTCF loop interactions (Fig. 1k and Supplementary Fig. 1n, blue line)^18^, with TSA-treated cells represented by the red loop-line. The ratio of the blue (DMSO) loop-line to the red (TSA) loop-line showed minimal differences in loop strength (Fig. 1k and Supplementary Fig. 1n, bottom panel, dark line), indicating that TSA treatment has little impact on most CTCF-CTCF loop interactions. These results suggest that loops mediated by cohesin at pairs of CTCF sites are largely resistant to TSA-induced histone hyperacetylation.

We next quantified changes in intra-TAD interactions by calculating the average interaction frequency within TADs (see Methods). We observed that TSA treatment significantly reduced intra-TAD interactions across replicates. Notably, we also detected a reduction in TAD boundary insulation strength. Rather than implying a causal relationship, we interpret this reduction as a consequence of how insulation scores are calculated: a decrease in intra-TAD interactions results in an apparent weakening of boundary insulation. (Fig. 1l and Supplementary Fig. 1o).

Histone hyperacetylation reduces nucleosome interactions and decompacts chromatin fibers^25,34^, increasing its contour length, which may contribute to the reduction of intra-TAD interactions. To assess how much chromatin decompaction decreases intra-TAD interactions, we first depleted all cohesin using RAD21-degron cells^35^, followed by treatment with either DMSO or TSA. As cohesin loss eliminates TAD structures, TADs were defined using the DMSO-treated, cohesin-intact condition, and changes in short-range interactions were quantified within these regions (Fig. 1m). As expected, eliminating cohesin significantly reduces intra-TAD interactions (Fig. 1n and Supplementary Fig. 1p, two replicates, the first and third blue violin plots in each replicate). TSA treatment further decreases intra-TAD interactions in the chromosomes without cohesin (Fig. 1n and Supplementary Fig. 1p, the fourth red violin plots in each replicate). We then compare the difference between TSA and DMSO treatments with or without cohesin degradation. The decrease in intra-TAD interactions in cells without cohesin accounts for ~50% of the decrease of intra-TAD interactions in cells with cohesin. Therefore, our results showed that ~50% of the reduction of intra-TAD interactions reduced by TSA is due to reduced cohesin-mediated interactions within TADs. Further, the level of intra-TAD interactions in TSA-treated cells is comparable whether cells express cohesin or not. This is consistent with the model that TSA treatment leads to both chromatin decompaction and loss of cohesin-mediated intra-TAD loops. In summary, these results suggest that cohesin within TADs is selectively sensitive to TSA-induced histone hyperacetylation.

We next assessed the reduction of cohesin-mediated short-range interactions across genomic distances using *P(s)* analysis. TSA-induced changes in *P(s)* were compared in the absence and presence of cohesin, and their ratio was plotted (see Methods; Fig. 1o and Supplementary Fig. 1q for two independent replicates. The corresponding *P(s)* plots are shown in Supplementary Fig. 1r–s). Interestingly, the reduction in contact frequency resulting from chromatin contour length changes varied with genomic distance, showing minimal impact around 300 kb and maximal impact near 2 Mb. Since most cohesin-mediated intra-TAD interactions occur within 500 kb, our results suggest that the reduction of cohesin-mediated interactions accounts for approximately 40% of the observed reduction in interaction frequency. Notably, the *P(s)* ratio (absence vs. presence of cohesin) exceeds 1 in the >2 Mb range, indicating that loss of short-range loops leads to less decay at larger distances.

To further validate that cohesin-mediated interactions are altered by TSA-induced histone hyperacetylation, we performed RAD21-targeted ChIP-loop and PLAC-seq^36,37^, both of which specifically capture and enrich cohesin-mediated chromatin interactions (See Methods). These analyses revealed a marked reduction in cohesin loop signals following TSA treatment. Notably, TSA-induced histone hyperacetylation led to decreased intra-TAD interactions while having minimal impact on CTCF–CTCF loop interactions, consistent with our observations from Hi-C analysis (Fig. 1p–r and Supplementary Fig. 1t–v, two independent replicates ChIP-Loop, Supplementary Fig. 10e, another replicate of ChIP, Supplementary Fig. 10f. one replicate of PLAC-seq).

Taken together, our findings demonstrate that TSA-induced histone hyperacetylation reduces both nucleosome and cohesin-mediated looping interactions, resulting in significantly weakened intra-TAD interactions while having minimal impact on CTCF-CTCF loops.

SMC3 acetylation, which is regulated by HDAC8^38^, could also be modulated by TSA, potentially affecting cohesin-mediated interactions. To test this, we examined both the levels of acetylated SMC3 and the chromatin affinities of SMC3 and acetylated SMC3 following TSA treatment. We observed no significant changes in acetylated SMC3 levels or its chromatin affinities (Supplementary Fig. 2a). These results suggest that the loss of cohesin-mediated loops induced by TSA treatment is not attributable to changes in SMC3 acetylation.

In HAP1-RAD21^TEV^ cells, RAD21 contains a tandem TEV motif in the unstructured region^18^, which could potentially influence cohesin-mediated loops and contribute to the changes observed following TSA treatment. To assess the impact of TSA treatment on looping interactions, we examined HAP1 wild-type cells and found that TSA treatment led to similar effects on both compartmentalization and cohesin loops. These results indicate that the inserted TEV sequence in HAP1-RAD21TEV cells does not account for the observed changes following TSA treatment (Supplementary Figs. 2 and 3).

### Impact of histone hyperacetylation on chromatin binding of cohesin and CTCF

**Fig. 2:**
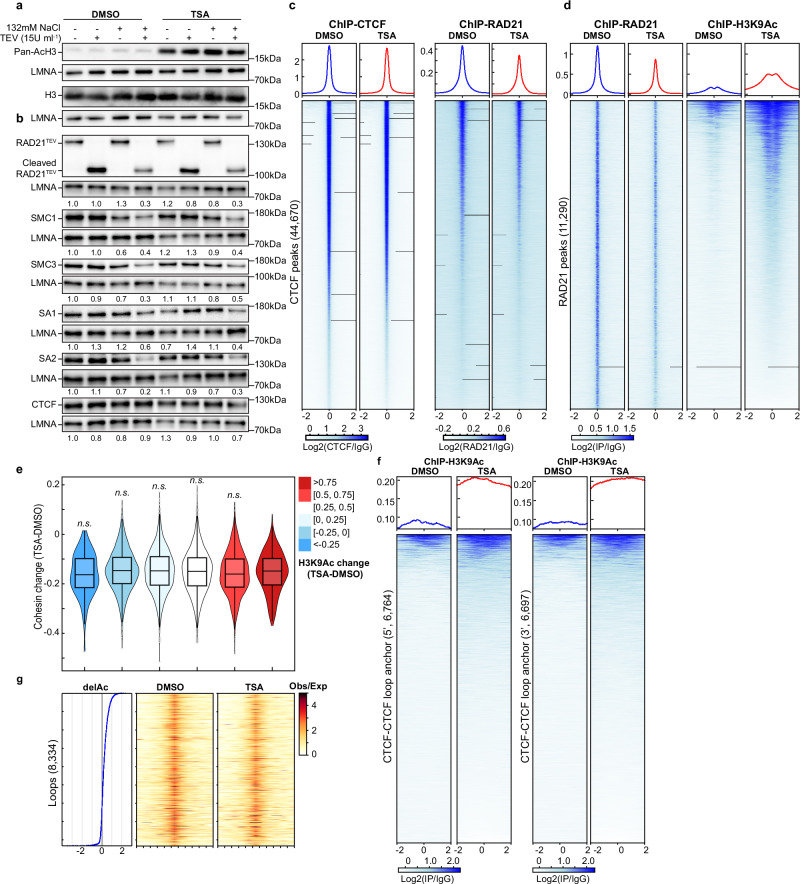
Impact of histone hyperacetylation on chromatin binding of CTCF and cohesin and on CTCF-CTCF loop anchors. **a** Western blot analysis of total histone H3 and acetylated histones using a pan-acetylated histone antibody. **b** Western blots of chromatin-associated cohesin subunits in DMSO- or TSA-treated HAP1-RAD21^TEV^ nuclei under low-salt (NB) or high-salt (NBS1; NB  +  132 mM NaCl) conditions, with or without TEV protease. LMNA was used as a loading control. Cohesin levels were normalized to DMSO-treated nuclei in NB buffer without TEV. Data shown are representative of one of three independent replicates; additional replicates are provided in Supplementary Fig. 4a and h. **c** CTCF (left) and RAD21 (right) ChIP-seq signals at 44,670 CTCF binding sites identified in DMSO-treated cells. Top: average profiles. Bottom: heatmaps across all sites. **d** RAD21 (left) and H3K9Ac (right) ChIP-seq signals at 11,290 RAD21 binding sites. Top: average profiles. Bottom: heatmaps. **e** Relationship between histone acetylation and RAD21 binding. RAD21 binding changes are plotted across bins of H3K9Ac signal changes (±2 kb from RAD21 peaks) using violin plots with embedded boxplots. Statistical significance was assessed by comparing each bin to the highest H3K9Ac bin using a two-tailed Wilcoxon rank-sum test. Boxplots represent the interquartile range (25th−75th percentiles) with the median indicated (see Methods). **f** H3K9Ac ChIP-seq signals at 5′ and 3′ loop anchors (6764 and 6697 unique anchors; 8334 loops^12^). **g** Relationship between histone acetylation and loop strength. Left: ranked changes in combined H3K9Ac signal at loop anchors. Right: heatmaps of loop strength (loop-lines) under each condition. Color scale indicates contact frequency normalized by expected cis-interactions. All ChIP-seq data in (**d**–**g**) are from replicate 1 (DMSO or TSA). Data shown are representative of two independent replicates; the second replicate is provided in Supplementary Fig. 4. Source data are provided.

Histone acetylation alters the charge properties of chromatin, it may thus alter cohesin and chromatin interactions and disrupt loop extrusion, leading to the loss of cohesin-mediated loops within TADs. We thus used an assay we developed previously that can examine how TSA treatment, likely histone hyperacetylation, affects interactions between cohesin and chromatin^18^ (see below). First, we investigated whether TSA affects chromatin binding of cohesin using purified nuclei from DMSO- or TSA-treated cells. Western blotting confirmed that histone acetylation induced by TSA treatment was preserved in the purified nuclei (Fig. 2a and Supplementary Fig. 4a bottom panels), indicating that chromatin states in purified nuclei are comparable to those in intact cells. We then added TEV protease to the purified nuclei and cleaved RAD21 under low or physiological salt concentrations and assessed the chromatin association of cohesin subunits. Surprisingly, western blotting indicated that TSA treatment had minimal effects on the chromatin retention of both CTCF and cohesin core subunits, including RAD21, SMC1, SMC3, SA1 and SA2, in all the various conditions (Fig. 2b and Supplementary Fig. 4a). These results suggest that, despite histone hyperacetylation reducing cohesin-mediated loops within TADs, the global chromatin binding of cohesin core components remains unchanged.

We next performed ChIP-seq analysis to investigate the effects of histone hyperacetylation on CTCF and cohesin binding to chromatin (Fig. 2c and Supplementary Fig. 4b). Unlike Western-based methods that assess global chromatin binding, ChIP-seq specifically profiles enriched binding sites, particularly cohesin occupancy at CTCF sites. While CTCF binding at CTCF sites was unaffected by TSA treatment, RAD21 ChIP signals at CTCF-bound sites showed some reduction. Intriguingly, this reduction occurred despite the relative stability of CTCF-CTCF looping interactions under conditions of histone hyperacetylation. Further, RAD21 ChIP-seq signals also showed a general decrease in cohesin-enriched sites following histone acetylation (Fig. 2d and Supplementary Fig. 4c, left panels), indicating a general reduction of cohesin binding at specific sites.

We next assessed histone hyperacetylation. We focused on H3K9Ac ChIP signals, which are enriched in promoter and open chromatin regions^39^. TSA treatment led to a ~50% increase in H3K9Ac peaks (Fig. 2d and Supplementary Fig. 4c, right panels). However, we did not detect a direct correlation between the increased H3K9Ac ChIP signals and the decrease in RAD21 ChIP signals (Fig. 2e and Supplementary Fig. 4d).

We then examined changes in H3K9Ac ChIP signals at CTCF-CTCF loop anchors. TSA treatment increased H3K9Ac levels at both 5’- and 3’- loop anchors (Fig. 2f and Supplementary Fig. 4e), although the majority of anchors showed minimal changes. We next ranked loop anchors by degree of H3K9Ac increases and compared loop line signals between DMSO- and TSA-treated cells (Fig. 2g and Supplementary Fig. 4f). A modest reduction in loop line signals was observed upon TSA treatment, as reflected by the loop-line pileup analysis (Fig. 1k, Supplementary Fig. 1n, Fig. 2g and Supplementary Fig. 4f). However, this reduction did not scale with H3K9Ac levels. Together, these results confirm that CTCF-anchored loops are largely maintained despite histone hyperacetylation.

Taken together, our results indicate that cohesin binding shows minimal changes within TADs, while cohesin-mediated loops within TADs are reduced. This suggests that although cohesin can still associate with acetylated chromatin within TADs, its ability to tether two loci and mediate loop formation is reduced. In contrast, cohesin binding at individual CTCF sites is reduced, while CTCF-CTCF looping interactions remain largely unchanged.

### Histone hyperacetylation increases the sensitivity of CTCF looping interactions to cohesin ring integrity

**Fig. 3:**
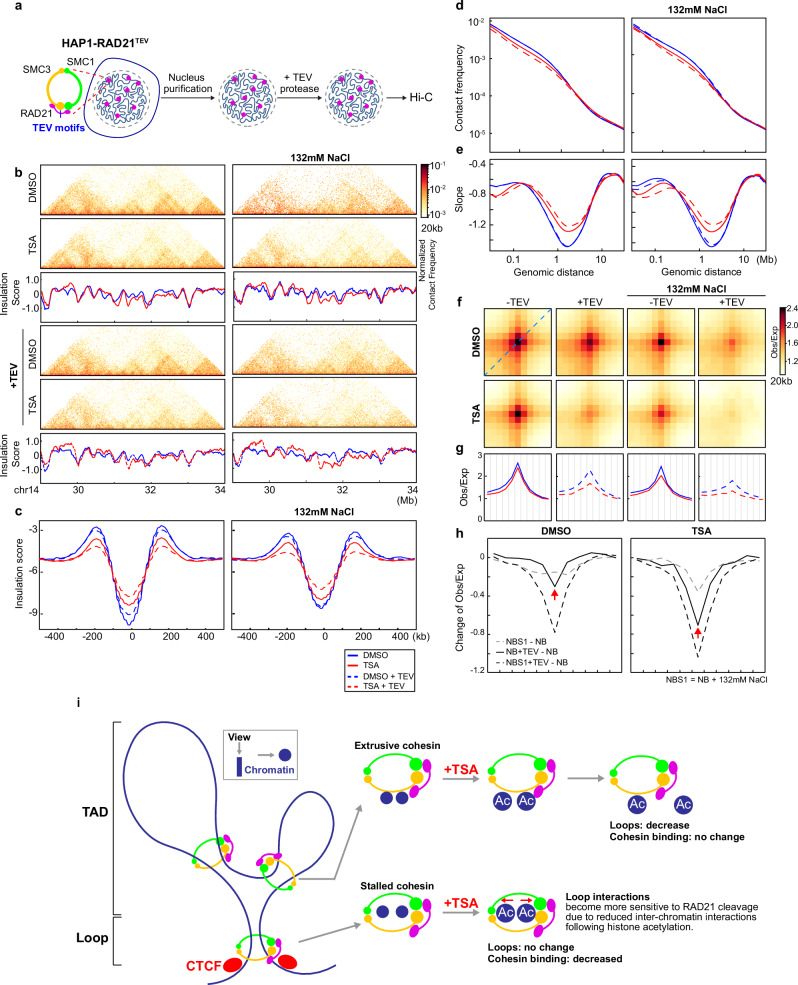
Histone hyperacetylation increases the sensitivity of cohesin at CTCF-CTCF loop anchors to RAD21 cleavage. **a** A schemetic of semi-in vitro assay. Tandam TEV motifs were inserted to RAD21 using CRISPR to generate HAP1-RAD21^TEV^ cells^18^. Nuclei were purified from HAP1-RAD21^TEV^ cells and treated with TEV protease in different salt concentrations before Hi-C or Western blotting analysis. **b** Examples of Hi-C maps obtained with DMSO- or TSA-treated HAP1-RAD21^TEV^ nuclei treated with or without TEV in NB (left) or NBS buffer (right). Insulation profiles (bottom) for the same region for each condition. **c** Aggregate Hi-C data at TAD boundaries identified in DMSO- or TSA-treated nuclei treated with or without TEV in NB (left) or NBS1 (right) buffer. **d**, **e** *P(s)* plots (**d**) and plots of their derivatives (**e**) for Hi-C data from DMSO- or TSA-treated nuclei with or without TEV treatment in NB (left) or NBS1 (right) buffers. The arrows indicate the signature of cohesin loops. **f** Aggregate Hi-C data at loops identified in HAP1 cells (as in Fig. 1j; top). **g** Loop-lines for each condition. In each panel, red and blue lines indicate DMSO or TSA treatments and dash lines indicate TEV protease treatment. **h** The difference of loop-lines (all other conditions versus NB buffer), left and right panels are DMSO versus TSA treatment. **i** A summary of distinct responses of cohesin-mediated loop interactions within TADs and at CTCF-CTCF sites. Cohesin-mediated looping interactions within TADs are reduced upon histone hyperacetylation, while cohesin-mediated loop interactions at CTCF-CTCF binding sites are resistant to histone hyperacetylation. Since chromatin with hyperacetylated histones exhibits weakened inter-chromatin interactions (red arrows), RAD21 cleavage results in a greater loss of loop interactions between pairs of CTCF sites. Dark blue dots indicate chromatin viewed from the top. Source data are provided as a Source Data file.

Others and we have shown that cohesin entraps chromatin at the base of CTCF-CTCF loops. This topological mode of chromatin binding may contribute to the stability of loop interactions between pairs of CTCF sites after TSA treatment. We thus sought to explore how histone hyperacetylation influences cohesin association with chromatin at CTCF-CTCF loop sites. The semi-in vitro nuclear system, established previously, enables controlled perturbations of cohesin interactions with chromatin^18^, making it ideal for measuring cohesin-chromatin interactions at CTCF-CTCF loop sites following TSA treatment (Fig. 3a). We purified nuclei from DMSO- or TSA-treated cells, then cleaved RAD21 under low or physiological salt conditions to assess cohesin engagement with chromatin at these sites.

Hi-C maps generated from purified nuclei revealed that TSA treatment did not cause significant alterations in compartment boundaries or compartment interaction strength, consistent with the Hi-C analysis performed on the intact cells from which the nuclei were purified (Supplementary Fig. 5a, b and Supplementary Fig. 6a, b). We then examined local features of chromosome structure following TSA treatment. Hi-C maps showed that RAD21 cleavage under both salt buffers led to minimal changes in local chromatin structures (Fig. 3b and Supplementary Fig. 6c). As anticipated, TSA treatment induced notable changes in certain local chromatin features (Fig. 3b and Supplementary Fig. 6c). To quantify the loss of local features, we calculated insulation profiles (Fig. 3b and Supplementary Fig. 6c, blue versus red lines, representing DMSO and TSA treatments, respectively). Consistent with our Hi-C analysis in cells, we observed significant changes in some domains, including changes in domain boundary strength following TSA treatment.

We then assessed the insulation strength by aggregating interactions at and around TAD boundaries. Consistent with previous studies^18^, both salt treatment and RAD21 cleavage weakened the insulation strength, as indicated by the blue plots in both panels (Fig. 3c and Supplementary Fig. 6d). Interestingly, TSA treatment also weakened insulation strength at TAD boundaries, as evidenced by the red plots in both panels (Fig. 3c and Supplementary Fig. 6d). These effects suggest that the impact of TSA on genome folding is distinct from the effects of salt or RAD21 cleavage, highlighting a unique mode of action for TSA in chromatin organization.

We then analyzed how interaction frequency decayed with genomic distance. Interestingly, short-range interactions (<1 Mb) in TSA-treated nuclei were more sensitive to RAD21 cleavage (Fig. 3d and Supplementary Fig. 6e, dashed red lines), while long-range interactions (>1 Mb) showed minimal changes across TSA, RAD21 cleavage, and salt treatments. These differences are further highlighted in the derivative of *P(s)*, where cleaving RAD21 in TSA-treated nuclei resulted in more pronounced changes to cohesin density peaks, as evidenced by the difference between the solid and dashed red lines compared to the blue lines (Fig. 3e and Supplementary Fig. 6f). These results indicated that TSA treatment mostly reduces cohesin-mediated loops within TADs, consistent with our living cell results, while with TSA and cleavage, all loops are reduced, including non-extruding loops at CTCF-CTCF sites.

In our previous studies, we showed that cohesin topologically engages chromatids at CTCF-CTCF loop sites, rendering these loops sensitive to RAD21 cleavage^18^. Based on our observation that histone hyperacetylation has minimal impact on CTCF-CTCF loop interactions, we hypothesize that histone hyperacetylation does not alter the mode by which cohesin engages chromatin at CTCF-CTCF loop sites, thereby preserving loop sensitivity to RAD21 cleavage. To test this, we examined the effect of TSA treatment on CTCF-CTCF loop formation. In DMSO-treated nuclei, we detected strong focal enrichment of interactions between loop anchors, whereas in TSA-treated nuclei, this enrichment remained largely unchanged (Fig. 3f and Supplementary Fig. 6g, left two heatmaps). In salt buffers, TSA treatment slightly weakened loop interactions (Fig. 3f and Supplementary Fig. 6g, third column of heatmaps), and cleaving RAD21 resulted in almost complete loss of loop interactions (Fig. 3f and Supplementary Fig. 6g, second and fourth columns of heatmaps).

**Fig. 4:**
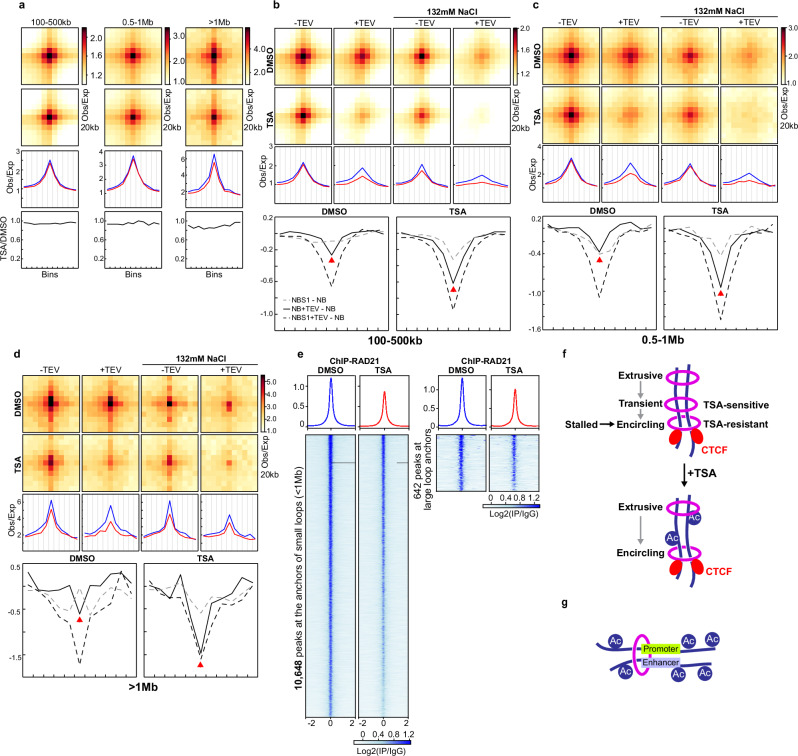
CTCF-CTCF loops tolerate cohesin loss induced by histone hyperacetylation, regardless of loop sizes. **a** Averaged Hi-C signals at chromatin loops of three different loop sizes identified in HAP1 cells^12^, and the associated loop-lines and ratios of loop lines as in Fig. 1j-k. The Hi-C results are from HAP1 cells treated with DMSO or TSA. **b**–**d** Aggregate Hi-C data at loops of different sizes identified in HAP1 cells^12^. Middle and bottom panels indicate loop-lines and differences between loop-lines as in Fig. 3f–h. **e** Average RAD21 ChIP–seq signals in each indicated condition for 642 binding sites that overlapped with the anchors of CTCF-CTCF large loops (1 Mb, right) and the remaining 10,648 binding sites (left). **f** Two distinct cohesin populations at CTCF-CTCF sites: a TSA-sensitive, transient form and a TSA-resistant form that encircles pairs of CTCF loop anchors. Histone hyperacetylation selectively dissociates the transient cohesin. **a**–**e** The red and blue lines represent samples treated with and without TSA, respectively. **g** Proposed model illustrating how cohesin stabilizes enhancer–promoter interactions by topologically encircling highly acetylated chromatin at transcription start sites. Source data are provided as a Source Data file.

To quantify CTCF-CTCF loop strength, we plotted the loop-line for each condition, as defined and calculated by the blue dashed line in the top heatmap of Fig. 1k. Loop-lines showed minimal changes following TSA treatment (Fig. 3g and Supplementary Fig. 6h, left panel). In salt buffers, TSA treatment resulted in slight changes to the loop lines (Fig. 3g and Supplementary Fig. 6h, third panel). Notably, cleaving RAD21 in TSA-treated nuclei caused a significant decrease in loop peaks (Fig. 3g and Supplementary Fig. 6h, second and fourth columns). We further quantified these changes by subtracting the loop lines in low-salt buffer without RAD21 cleavage to generate relative loop-lines (Fig. 3h and Supplementary Fig. 6i). The depth of the valley indicates a reduction in loop interaction strength. Cleaving RAD21 in TSA-treated nuclei resulted in a deeper valley (Fig. 3h and Supplementary Fig. 6i, dark solid and dashed lines). Interestingly, we found that all loops, regardless of size, were more sensitive to RAD21 cleavage after TSA treatment (Fig. 4b–d and Supplementary Fig. 7b–d). In summary, these results indicate that in TSA-treated nuclei, cohesin continues to entrap chromatin at CTCF-CTCF loops in the same pseudotopological manner as in DMSO-treated nuclei. Although histone hyperactylation reduces cohesin binding at CTCF sites and increases susceptibility of loops to RAD21 cleavage, the act of encircling chromatin enables cohesin loops to tolerate the chromatin hyperacetylation induced by TSA (Fig. 3i).

### Two populations of cohesin at CTCF-CTCF sites

Our analysis of reduced intra-TAD loop interactions indicates that loop extrusion within TADs is disrupted in TSA-treated cells. We initially hypothesized that the longer cohesin extrudes along chromatin, the greater the chance it is lost during TSA treatment, which would preferentially weaken large CTCF-CTCF loops. However, we observed only minimal reduction in interactions at large loops (>1 Mb; Fig. 4a and Supplementary Fig. 7a). One possible explanation is that these loops are held by cohesin that had already stalled at CTCF sites prior to TSA treatment and remained stably bound throughout the 3-hour incubation. Supporting this idea, loops of varying sizes exhibited similar increases in sensitivity to cohesin cleavage, suggesting that stalled cohesin accumulates at CTCF-CTCF loop anchors and encircles pairs of CTCF sites in a stable pseudotopological manner, regardless of loop size (Fig. 4b–d and Extended Data Fig. 7b–d).

We next examined cohesin occupancy at loop anchors of different loop sizes. Large loop anchors showed comparable reductions in cohesin binding relative to small and medium loops, and occupancy of cohesin is also similar (Fig. 4e and Supplementary Fig. 7e). These results indicated that similar amounts of cohesin are lost across loop sizes, while the remaining cohesin continues to associate with chromatin at CTCF sites and maintain looping interactions.

These findings suggest the existence of two distinct populations of cohesin at CTCF-sites (Fig. 4f). One population appears sensitive to histone hyperacetylation and likely corresponds to cohesin that has not yet encircled chromatin-possibly holding chromatids as an extrusive cohesin within TADs. The other, predominant population encircles the CTCF sites and remains resistant to TSA-induced chromatin hyperacetylation. We refer to the first population of cohesin as transient, TSA-sensitive cohesin, as it transitions toward topologically entrapping chromatin but still exhibits sensitivity to TSA, similar to extrusive cohesin.

This model suggests a two-stage process for cohesin stalling at CTCF sites: in the first stage, TSA-sensitive cohesin either arrives at CTCF sites while extruding or is newly loaded there, but remains in a non-encircling state that is susceptible to histone hyperacetylation; in the second stage, chromatids become fully entrapped within the cohesin ring, establishing a more stable, TSA-resistant loop structure. The second stage appears to occur only when both loop anchors are at CTCF sites.

## Discussion

TSA-induced histone hyperacetylation weakens intra-TAD interactions but leaves CTCF-anchored loops largely unaffected (Fig. 3i), revealing distinct sensitivities between two classes of cohesin-mediated chromatin loops. In our previous work, we showed that cohesin engages chromatin through mechanistically distinct modes: within TADs, cohesin extrudes loops via non-topological engagement, whereas at pairs of convergent CTCF sites, cohesin topologically entraps DNA to form stable, stalled loops^18^. These biophysical differences likely underlie the differential responses to chromatin hyperacetylation.

Extruding cohesin acts through a dynamic cycle of transient chromatin interactions^40–43^. For example, the SMC-RAD21 interface initially clamps chromatin, followed by projection of the SMC1-SMC3 hinge that promotes movement along the chromatin fiber^44^. These interacting surfaces may be susceptible to changes in chromatin charge and conformation. Histone hyperacetylation alters chromatin surface charge, which appears to result in impaired extrusion activity and weakened intra-TAD interactions, whereas chromatin binding by cohesin is largely unaffected (Supplementary Fig. 8g, two independent replicates). This suggests that cohesin has at least two interaction surfaces with chromatin that are both needed to hold the two anchors of a chromatin loop, and that chromatin binding of only one of these is TSA-sensitive.

By contrast, cohesin rings that topologically entrap chromatin at looped pairs of CTCF–CTCF sites form a more stable configuration, supporting persistent looping interactions that remain largely intact following TSA-induced chromatin hyperacetylation. This resistance highlights the functional and mechanistic distinction between non-topologically and topologically bound cohesin complexes.

Interestingly, despite preserved CTCF-CTCF loops, we observe a marked reduction in cohesin ChIP-seq signal at CTCF loop anchor sites after TSA treatment. This suggests the presence of two cohesin populations at loop anchors. We propose that a TSA-sensitive population—likely consisting of newly loaded or transiently extruding, non-encircling cohesin—is preferentially depleted following chromatin hyperacetylation, whereas a TSA-resistant population—composed of topologically bound cohesin—maintains loop interactions. Supporting this model, RAD21 cleavage in TSA-treated nuclei abolishes CTCF-CTCF loops, confirming that maintenance of this type of loop depends on the DNA encircling form of cohesin (Fig. 3f–h).

The TSA-sensitive population may reflect cohesin in the process of transitioning between dynamic and stable binding modes. We propose two non-mutually exclusive scenarios. First, at a given loop anchor, CTCF may engage in multiple loop interactions across cells: some stabilized by topologically-bound cohesin holding two CTCF sites, and others formed via non-encircling complexes with distal non-anchor CTCF sites. Consistent with this, we showed in our previous work that only CTCF-CTCF loops are mediated by the topologically-bound form of cohesin, whereas loops where only one anchor is at a CTCF site are held by the extruding non-topological form^18^. In this scenario, loss of the latter population upon TSA treatment would reduce cohesin ChIP signal, loss of the CTCF-nonCTCF loops, but will not alter aggregate CTCF-CTCF loop strength. Alternatively, paused extruding cohesin may transiently accumulate at CTCF-bound anchors already occupied by stable topologically-bound cohesin. TSA-induced depletion of the extruding population would again reduce cohesin ChIP signal, while preserving the underlying loop that are maintained by the stably-bound cohesin complex. Both scenarios account for reduced cohesin occupancy with CTCF-CTCF loop maintenance and highlight the biochemical heterogeneity of cohesin at loop bases.

Histone hyperacetylation reduces nucleosome interactions by altering the surface charge of nucleosomes, thereby weakening chromatin-chromatin interactions between neighboring chromatin regions^25^. At pairs of looping CTCF sites, cohesin topologically holds two chromatin fibers together. As a result, although histone hyperacetylation weakens interactions between the two acetylated chromatin fibers, CTCF-CTCF loop interactions remain largely intact due to cohesin-mediated tethering. Upon RAD21 cleavage, opening of the cohesin ring releases both chromatin fibers. Because acetylated chromatin exhibits weakened inter-fiber interactions, loop contacts at CTCF–CTCF sites become more sensitive to cohesin ring cleavage compared to non-acetylated chromatin. This explains the increased sensitivity of CTCF loops to RAD21 cleavage following TSA treatment (Fig. 3i, bottom)

Together, our findings support a model in which cohesin exists in at least two functionally and biochemically distinct states: a dynamic, TSA-sensitive extruding form that promotes intra-TAD contacts, and a stable, TSA-resistant, topologically-engaged form that anchors CTCF–CTCF loops. Histone hyperacetylation selectively disrupts the extruding population while sparing the CTCF-CTCF loop-anchoring form, revealing how chromatin state modulates cohesin engagement and activity. Finally, it is intriguing that while loops mediated by the extruding form of cohesin are sensitive to TSA, chromatin association of cohesin is not. This suggests that cohesin has at least two interaction surfaces with chromatin that are both needed to hold the two anchors of a chromatin loop, and that chromatin binding of only one of these is TSA-sensitive.

Our previous results also showed that cohesin at transcription start sites (TSSs) is sensitive to cohesin ring opening, suggesting topological engagement with chromatin at these loci^18^. Given that TSSs are characterized by high levels of histone acetylation, the increased susceptibility of cohesin to RAD21 cleavage likely reflects an open chromatin state that promotes cohesin destabilization. Despite this sensitivity, cohesin at TSSs may play a structural role in stabilizing the transcriptional machinery and facilitating gene regulation (Fig. 4g). These findings highlight the critical importance of cohesin ring integrity in supporting transcriptional activity at hyperacetylated regions and underscore how chromatin state shapes cohesin dynamics on chromatin.

## Methods

### Cell culture and chemicals

HCT-116-mAID-RAD21 degron2 cells were kindly provided by Yesbolatova, et al., 2020^35^. These cells were cultured in McCoy’s 5A medium, GlutaMAX supplement (Gibco, 36600021) supplemented with 10% FBS (Gibco, 16000044) and 1% penicillin-streptomycin (Gibco, 15140) at 37 °C in 5% CO2. HAP1 cells were originally purchased from Horizon Genomics (Cambridge, UK, C859), and HAP1-RAD21^TEV^ cells were generated as previously reported^18^. Both HAP1 and HAP1-RAD21^TEV^ cells were cultured in IMDM medium, GlutaMAX supplement (Gibco, 31980097) supplemented with 10% FBS (Gibco, 16000044) and 1% penicillin-streptomycin (Gibco, 15140) at 37 °C in 5% CO_2_. Trichostatin A (#T8552) and 5’-ph-IAA (SML3574) were purchased from Sigma-Aldrich, USA, and dissolved in DMSO (#D2650) before treating the cells.

### Cell cycle analysis

For cell cycle analysis, cells were washed using 1xDPBS once and fixed in 90% ethanol at −20 °C for at least 24 hours. Fixed cells were washed in 1xDPBS and then resuspended in DPBS containing 2 mM MgCl_2_, 0.5 mg/ml RNase A (Roche, 10109169001), propidium iodide (50ug/mL). The samples were incubated at 20 °C for 30 min and then analyzed using a BD Symphony A5 flow cytometry instrument with yellow channels to monitor DNA content. FACS data were processed and analyzed using FlowJo v.10. Viability gates using forward and side scatter were set on each sample. DNA content was plotted as a histogram of the PI channel.

### Nucleus purification and TEV cleavage

Nuclei were purified according to Liu et al.^18^ Briefly, DMSO- or TSA-treated HAP1-RAD21^TEV^ cells were trypsinized, collected in medium, and counted. Around 100 million cells were collected and washed in cold PBS twice, and once in NB buffer (10 mM PIPES pH 7.4, 2 mM MgCl_2_, 10 mM KCl, protease inhibitor (PI, ThermoFisher, #78438)). The cells were then re-suspended in NB buffer with 0.1% NP-40, 1 mM DTT, and protease inhibitors, and incubated on ice for 10 minutes. The cells were lysed using a Dounce homogenizer with pestle A and then loaded onto a sucrose cushion (NB buffer + 30% sucrose + 1 mM DTT and 5 ml of cell lysis for 20 ml sucrose cushion), and finally centrifuged at 800 g for 10 minutes. The nucleus pellets were washed once in cold NB buffer, then resuspended in NB and counted. Around 4 million nuclei were plated onto 60 mm poly-D-lysine culture dishes, respectively (Corning BioCoat, #356469), and incubated at 4 °C overnight. For the TEV cleavage assay, 60 units of TEV enzyme (AcTEV Protease, Thermo Scientific, 12575-015) were added to 4 million nuclei in 4 ml buffer (15U/ml TEV) before the nuclei were plated onto the dishes and kept at 4 °C overnight. Control plates contained no TEV, otherwise, TEV concentrations were as indicated in the figures.

### Hi-C experiments

Hi-C for fixed cells or the fixed nuclei from the semi-in vitro assay was performed as described in our previous study^18^. Briefly, the fixed cells were first lysed to obtain nuclei. After being washed twice with cold NEBuffer 3.1, both the nuclei from fixed cells or from the semi-in vitro assay were 342ul NEBuffer 3.1 with 0.1% SDS and the tube was gently mixed. The tube was then incubated at 65 °C for 10 min and then put on ice immediately. Before 400U DpnII was added, 43 µL of 10% Triton X-100 was added and gently mixed. DpnII digestion was performed at 37 °C overnight with gentle rocking. Once enzyme digestion was done, the reaction was incubated at 65 °C for 15 minutes to inactivate DpnII. After this, the DNA overhang ends were filled in with biotin-14-dATP for DpnII-digested chromatin at 23 °C for 4 hours and then ligated with T4 DNA ligase at 16 °C for 4 hours. DNA was treated with proteinase K at 65 °C overnight to remove cross-linked proteins. Ligation products were purified, fragmented by sonication to an average size of ~200 bp and size-selected to fragments of 100-350 bp. We then performed end repair and dA-tailing and selectively purified biotin-tagged DNA using streptavidin beads. Illumina TruSeq adapters were added to form the final Hi-C ligation products, samples were amplified, and the PCR primers were removed. Hi-C libraries were then sequenced using PE50 or PE150 bases on an Illumina HiSeq 4000 or an Illumina NovaSeq instrument.

### Hi-C for G1 sorted cells

As previously reported^18^ to sort G1 cells for Hi-C analysis, the cells were fixed following the Hi-C protocol using 1% formaldehyde. The fixed cells were washed in 1×PBS and resuspended in PBS containing 2 mM MgCl_2_, 0.1% saponin, 0.5 mg/ml RNase A, and PI (50ug/mL in dimethyl sulfoxide). The samples were incubated at 20 °C for 30 min and then analyzed using a BD Symphony S6 flow cytometry instrument using the yellow channel to monitor the DNA content. To avoid obtaining any cells in S phase, only the cells in the left part of the G1 peak were collected. The FACS data were processed and analyzed using FlowJo v.10. Viability gates using forward and side scatter were set on each sample. The sorted G1 cells were then used to generate Hi-C libraries as described above.

### Hi-C and data analysis

Sequencing data of PE100 or PE150 was first trimmed to PE50 using the in-house scripts.

All Hi-C PE50 fastq raw sequencing files were mapped onto the hg19 human reference genome using the distiller-nf mapping pipeline (https://github.com/mirnylab/distiller-nf). After mapping, aligned reads were further processed to remove duplicates (https://github.com/mirnylab/pairtools) to obtain a set of filtered reads defined as valid pairs. Valid pairs were then binned into contact matrices at 20 kb, 100 kb and 200 kb resolutions using cooler^50^. Intrinsic Hi-C biases were removed using the Iterative Correction and Eigenvector decomposition (ICE) procedure^45^, which was applied to all of the matrices, ignoring the first two diagonals to avoid short-range ligation artefacts at a given resolution, and bins with low coverage were removed using the MADmax filter with default parameters. Contact matrices were stored in ‘.mcool’ files and used in downstream analyses.

For aggregation of loop interactions, the previously identified sets of HAP1 looping interactions were used^1,12^. In total, 8334 looping interactions are on the structurally intact chromosomes of HAP1. To visualize the looping interactions, we aggregated 20 kb binned data at all loops using Cooltools. We also aggregated 20 kb binned data at different sizes of loops, 100 kb–500 kb, 500 kb–1 Mb and greater than 1 Mb. The size of a loop refers to the distance between the two loop anchors.

For *P*(s) plots and derivatives, the cis reads from the cooler files were used to calculate the contact frequency *(P)* as a function of genomic separation *(s)* (cooltools). All of the *P*(*s*) curves were normalized using expected cis interactions (cooltools compute-expected) in each dataset. Corresponding derivative plots were calculated from each *P*(s) plot.

For the ratio of TSA-induced *P(s)* changes in the absence versus the presence of cohesin, the difference of *P(s)* between DMSO and TSA treatments was first calculated, then the ratio of the difference in the absence versus the presence of cohesin was obtained.

To calculate the ratio of TSA-induced *P(s)* changes in the absence versus the presence of cohesin, the difference in *P(s)* between DMSO- and TSA-treated samples under each condition was determined. Then the ratio of these differences between cohesin-depleted and cohesin-intact samples was calculated and plotted along genomic distance.

For interaction aggregation at TAD boundaries, we first calculated observed/expected Hi-C matrices of each sample for 20 kb binned data, correcting for average distance decay as observed in the *P*(s) plots (cooltools compute-expected). We then aggregated the observed/expected Hi-C matrices of each sample at the TAD boundaries that were identified from the sample without any treatments, covering 600 kb up and downstream of each boundary, and then generated a pileup heatmap of TAD boundaries for each sample.

For average interactions within TADs, we first identified TAD boundaries by calculating the genome-wide contact insulation score using a 20 kb resolution and boundary calling using the Li threshold (https://github.com/open2c/cooltools, version 0.3.0). Each TAD region was determined by taking the end position from a TAD boundary and the start position for the subsequent boundary. For each TAD region, the diagonal signal is removed, and the number of additional bins removed from the diagonal and edges of the TAD is set by the TAD sizes. The intra-TAD intensity is calculated by taking the average contact score of the remaining bins. Outliers in the intra-TAD intensity were removed using the interquartile range. The intra-TAD intensity violin plot was visualized using matplotlib and seaborn.

For compartment analysis, compartment boundaries were identified in cis using eigenvector decomposition on 100 kb binned data with the cooltools call-compartments function. A and B compartment identities were assigned by gene density tracks such that the more gene-dense regions were labeled A compartments, and the PC1 sign was positive. Changes in compartment type therefore occur at locations where the value of PC1 changes sign. Compartment boundaries were defined at these locations, except for when the sign change occurred within 400 kb of another sign change.

To measure compartmentalization strength, we calculated observed/expected Hi-C matrices for 100 kb binned data, correcting for average distance decay as observed in the *P*(*s*) plots (cooltools compute-expected). We then arranged observed/expected matrix bins according to their PC1 values of the sample without any treatments in each replicate. We aggregated the ordered matrices for each chromosome within a dataset and then divided the aggregate matrix into 50 bins. Strength of compartmentalization was defined as the ratio of (A-A  +  B-B)/(A-B  +  B-A) interactions. Strength of A-A and B-B interactions was separately calculated using AA/AB and BB/BA, respectively. The values used for this ratio were determined by calculating the mean value of the 10 bins in each corner of the saddle plot.

### ChIP-Loop and PLAC-seq

ChIP-loop experiments were performed as previously described with some modifications^37^. Briefly, all Hi-C steps were carried out except for biotin-14-dATP fill-in, biotin removal, and enrichment using streptavidin beads. PLAC-seq experiments were performed as previously described^36^, with the modification that nuclei were digested using DpnII. Briefly, the protocol followed standard Hi-C procedures up to the nuclear ligation step. After ligation, instead of proteinase K treatment and overnight incubation at 65 °C, ChIP was performed directly on the ligated nuclei. Subsequent steps followed our standard ChIP-seq protocol until purification of ChIP DNA. The purified ChIP DNA was then enriched for biotin-labeled ligation products using streptavidin beads, following Hi-C procedures. Finally, sequencing libraries were prepared from the bead-bound DNA according to the Hi-C library preparation protocol.

All ChIP-loop and PLAC-seq datasets were processed using the same Hi-C analytical pipeline described above, including read mapping, generation of multi-resolution cooler (mcool) files, derivative plot analysis, calculation of intra-TAD interactions, and CTCF-CTCF loop pileup analysis.

### Western blot for cohesin components

For each condition, 4 million nuclei were plated on 60 mm poly-Lysine-coated plates in 4 ml buffer at 4 °C for overnight. To analyze cohesin proteins retained in nuclei, all buffers were completely removed from each plate, and 200 µL RIPA buffer (Thermo Fisher, #89900) containing protease inhibitor and TurboNuclease (Accelagen, #N0103M) was added to each plate. All the plates were then incubated at 4 °C for 10 minutes, and the lysed nuclei were scraped and collected. After being spun at 8000 *g* for 3 minutes, the supernatant was transferred to a new tube and 5x sample buffer was added. After mixing, the lysis was boiled at 100 °C for 3 minutes for western blot analysis.

The volume for approximately the same number of cells or nuclei for each sample was loaded into each lane of a protein gel for separation. Two types of protein gel and buffer were used. To separate small proteins (MW < 50KD), NuPAGE 4–12% Bis-Tris protein gels (Thermo Fisher, #NP0322BOX) were used with NuPAGE MOPS SDS Running Buffer (Thermo Fisher, #NP0001). For large proteins (MW > 100 kD), NuPAGE 3-8% Tris-Acetate protein gels (Thermo Fisher, #EA03752BOX) were used in NuPAGE Tris-Acetate SDS running buffer (Thermo Fisher, #LA0041). Proteins were transferred to nitrocellulose membranes (Bio-Rad, #1620112) at 30 V for 2 hours in 1× transfer buffer (Thermo Fisher, #35040) in the cold room. The membranes were blocked with 5% milk in TBST (20 mM Tris-HCl, pH 7.4, 150 mM NaCl, and 0.1% Tween-20) for 30 minutes at room temperature. The membranes were then incubated with the specified primary antibodies diluted 1:1000 in TBST overnight at 4 °C. The membranes were washed three times with TBST for 10 min at room temperature each, then incubated with secondary antibodies (anti-rabbit IgG HRP-linked, Cell Signaling, 7074) diluted 1:5000 in TBST for 2 hours at room temperature. The membranes were then washed three times with PBS-T for 10 min each. Then, the membranes were developed and imaged using SuperSignal West Dura Extended Duration Substrate (Thermo, #34076) and Bio-Rad ChemiDoc with Image Lab 6.1.0.

### ChIP-seq experiments

ChIP-seq experiments were based on the protocol in our recent work with some minor modifications^18^. Briefly, 30 million cells were used for each condition, among which 10 million cells were used for each antibody. Cells were first washed once using DPBS, then fixed with 10 mL DPBS with 1% FA. After rocking for 10 min at RT, 540 µL of 2.5 M glycine was added and continued to rock for 5 minutes before being put on ice for at least 15 minutes. The cells were then washed once using DPBS before being collected into 1.7 mL tubes from the plates using a cell scraper. The cells were spun down at 1000 g at 4 °C for 10 min and DPBS was then removed. The pellet was then resuspended in 1 ml lysis buffer (20 mM Tris-HCl pH 8.0, 85 mM KCl, 0.5%IGEPAL, PI) and incubated for 15 min. The lysis was then spun at 1000 g at 4 C for 5 min and the lysis buffer was removed. The pellet was then resuspended in 900 µL sonication buffer (20 mM Tris-HCl pH 8.0, 0.2% SDS, 0.5% sodium deoxycholate, and protease inhibitor). The chromosomes were sonicated to fragments around 200-500 bp using a QSonica Q800R3 Sonicator (8 min sonication time). After spinning at 16,000 g for 10 min, the fragmented chromatin in the supernatant was split into aliquots of 5 million nuclei per tube and diluted in 1200ul IP buffer with a final 0.1% SDS concentration (20 mM Tris-HCl pH 8.0, 150 mM NaCl, 2 mM EDTA, 0.1% SDS, 1% Triton-100, and protease inhibitor). For each tube, around 4 ug of antibody was added, and for each antibody, 10 million nuclei were used for ChIP. The primary antibodies used in this study included CTCF (CST, #3418), RAD21 (Abcam, #ab154769, recognizes N-terminal of RAD21), RAD21 (Abcam, #ab992, recognizes the C-terminus of RAD21), and Rabbit IgG (Sigma, #I-5006). Chromatin was incubated with primary antibodies on a rocker at 4 °C overnight. After rewashing with IP buffer, 20ul of Dynabeads Protein A (Thermo Fisher, #10001D) was added to each tube, followed by incubation on a rocker at 4 °C for 2 hours. After the beads were washed for three times, immunoprecipitated DNA was eluted and 5 ng ChIP DNA was used to prepare sequencing libraries in the same way as described for Hi-C above.

### ChIPseq data analysis

Sequencing reads were first trimmed to 50 bp long before they were mapped onto hg19 using Bowtie 2. HOMER 4.6 was used to clean mapped reads, examine quality of ChIP experiments, call peaks, and generate visualization files^46^. Both profile and cluster plots of ChIPseq signals were generated using Deeptools 3.0.2^47^.

### Boxplot

All the boxplots were drawn using the boxplot() function in R with default settings; the box starts in the first quartile (25%) and ends in the third quartile (75%), with the line in the box indicating the median. The outliers are the data points that are greater than Q3 + 1.5*IQR (upper outlier), or less than Q1-1.5*IQR (lower outlier). Q1 and Q3 refers to the first and third quartile, respectively. IQR refers to internal-quartile range.

### Statistics and Reproducibility

No statistical method was used to predetermine sample size. Unless specified in the legends, Western blot analyses have been performed on two independent replicates. All Hi-C and ChIPseq analyses have been performed for two biological replicates. Both biological replicates were processed, analyzed, and visualized independently and exhibited consistent patterns, as described in the main text.

### Reporting summary

Further information on research design is available in the Nature Portfolio Reporting Summary linked to this article.

## Supplementary information

Supplementary Information

Reporting Summary

Transparent Peer Review file

## Source data

Source Data

## Data Availability

Deep-sequencing data that support the findings of this study have been deposited in the Gene Expression Omnibus (GEO) under accession code GSE288762. Source data are provided with this paper. All data supporting the findings of this study are available from the corresponding author on request. Source data are provided with this paper.

## Code availability

All programs used in this study are from Open Chromosome Collective (Open2C) and are publicly available in GitHub: https://github.com/open2c. No other customized codes were developed for this study.
