# Supplementary material for: Histone Acetylation Differentially Modulates CTCF-CTCF Loops and Intra-TAD Interactions
Source: Nat Commun. 2026 Jul 20;17:6676. doi: 10.1038/s41467-026-75818-8 (PMC13385825; doi:10.1038/s41467-026-75818-8)
Supplement: Supplementary file 1 — Supplementary Information [file 41467_2026_75818_MOESM1_ESM.pdf]

Supplementary Table 1

Western					
**Antibody**	**Company**	**Catalog #**	**Lot #**	**Ratio**	**Notes**
anti-RAD21	Abcam	ab154769	GR3224138-28	1:2000	Recognizes N-terminus
anti-RAD21	Abcam	ab992	GR3310168-15	1:2000	Recognizes C-terminus
anti-Lamin A/C	Abcam	ab26300	1046291-5	1:2000	
anti-SMC3	Abcam	ab9263	1003294-8	1:2000	
anti-SMC1	Bethyl lab	A300-055A	6	1:2000	
anti-CTCF	CST	3418	6	1:2000	
anti-Pan-Acetylated-H3	Abcam	ab4791	GR3454335-1	1:2000	
Anti-Acetylated-SMC3	Millipore	MABE1073	3316674	1:2000	
anti-SA1	Bethyl lab	A302-579A	1	1:2000	
anti-SA2	Bethyl lab	A302-580A	1	1:2000	
anti-H3	Abcam	ab1791	GR8237728-1	1:2000	
anti-H2AK5Ac	CST	2576S	3	1:2000	
anti-H2BK5Ac	CST	12799S	1	1:2000	
anti-Beta-Tublin	Abcam	ab6046	GR3243627-1	1:2000	
anti-H3K9Ac	CST	9649	13	1:2000	
anti-H3K27Ac	CST	8173	1	1:2000	
anti-H4K5Ac	CST	8647	1	1:2000	
anti-H4K8Ac	CST	2594	11	1:2000	
anti-rabbit IgG, HRP-linked	CST	7074	34	1:5000	
anti-mouse IgG, HRP-linked	CST	7076	38	1:5000	

ChIP					
**Antibody**	**Company**	**Catalog #**	**Lot #**	**Amount**	**Notes**
anti-RAD21	Abcam	ab992	GR3310168-15	6uL	Recognizes C-terminus
anti-CTCF	CST	3418	6	12uL	
anti-H3K9Ac	Abcam	ab4441	1069505-3	5uL	
anti-H3K27Ac	Abcam	ab4729	1086546-1	5uL	
anti-IgG	Sigma	I5006	SLCB7084	8uL	From rabbit serum

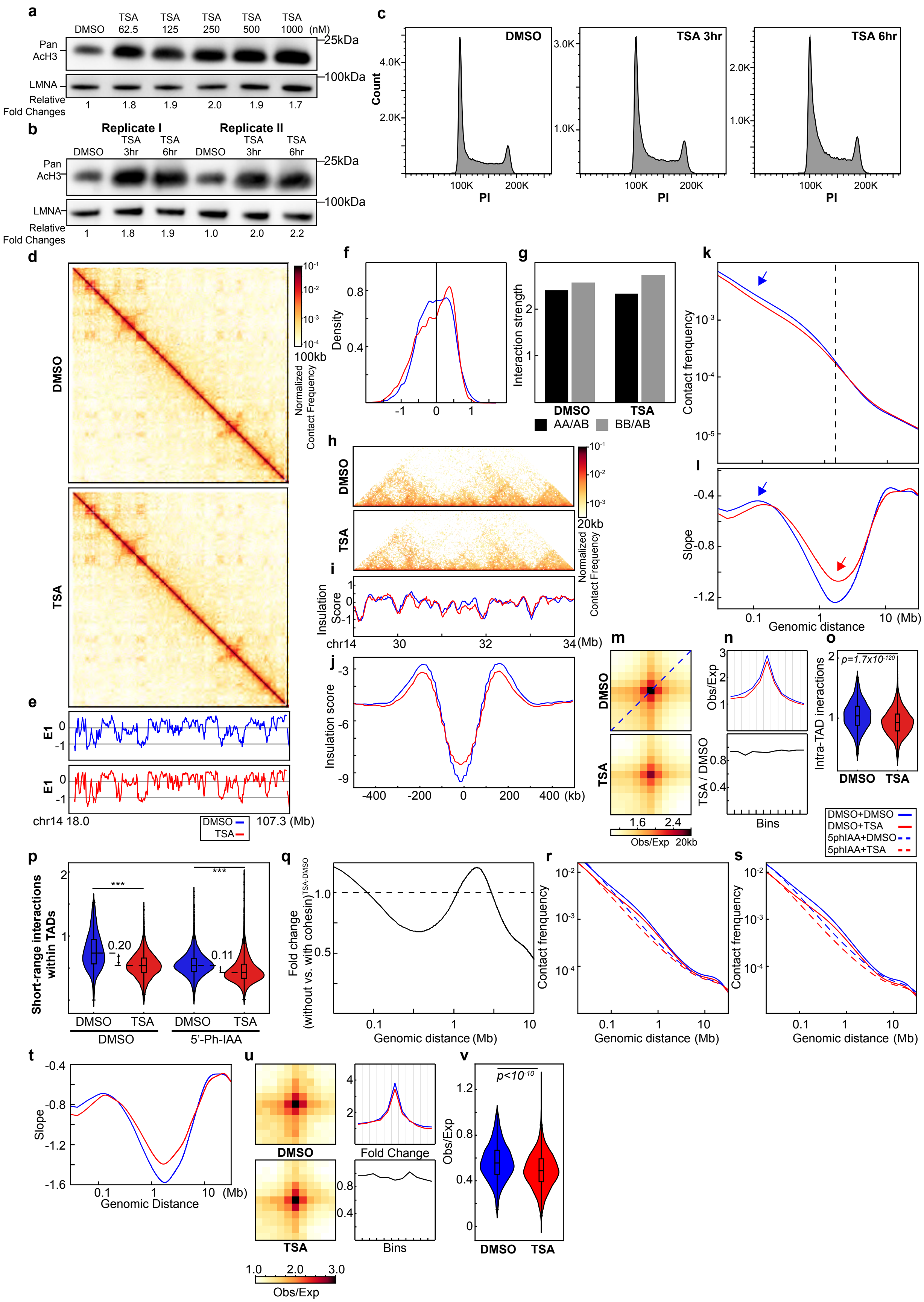
a
DMSO
TSA 62.5
TSA 125
TSA 250
TSA 500
TSA 1000 (nM)
Pan AcH3
25kDa
LMNA
100kDa
Relative Fold Changes
1 1.8 1.9 2.0 1.9 1.7
b
Replicate I
Replicate II
DMSO TSA 3hr TSA 6hr DMSO TSA 3hr TSA 6hr
Pan AcH3
25kDa
LMNA
100kDa
Relative Fold Changes
1 1.8 1.9 1.0 2.0 2.2
c
DMSO
TSA 3hr
TSA 6hr
Count
PI
d
DMSO
TSA
100kb
Normalized Contact Frequency
e
E1
chr14 18.0
107.3 (Mb)
DMSO
TSA
f
Density
g
Interaction strength
DMSO
TSA
AA/AB
BB/AB
h
20kb
Normalized Contact Frequency
i
Insulation Score
chr14
34 (Mb)
j
Insulation score
400 (kb)
k
Contact frenquency
l
Slope
Genomic distance
10 (Mb)
m
Obs/Exp 20kb
n
Obs/Exp
TSA / DMSO
Bins
o
p=1.7x10^-120
Intra-TAD ineractions
DMSO+DMSO
DMSO+TSA
5phIAA+DMSO
5phIAA+TSA
p
Short-range interactions within TADs
0.20
0.11
***
5'-Ph-IAA
q
Fold change (without vs. with cohesin)^TSA-DMSO
Genomic distance (Mb)
r
s
t
Genomic Distance
(Mb)
u
Fold Change
Bins
Obs/Exp
v
p<10^-10
Obs/Exp

Supplementary Fig. 1. TSA increases histone acetylation and alters chromatin interactions (replicate of Fig. 1). **a**, Levels of histone acetylation following TSA treatment at different concentrations for 3h. **b**, Histone acetylation after 500 nM TSA treatment for 3h and 6h; two replicates are shown. **c**, Cell cycle profiles following TSA treatment for 3h or 6h; DMSO (6h) serves as control. **d**, Hi-C interaction maps for HAP1 cells treated with DMSO or TSA (chromosome 14: 18.0-107.3 Mb). **e**, Eigenvector (E1) profiles across the same region. **f**, Distribution of E1 values, showing increased A-compartment signals after TSA treatment. **g**, Compartment interaction strength (A-A and B-B; see Methods). **h**, Hi-C maps for chromosome 14: 29-34 Mb. **i**, Insulation profiles for the same region. **j**, Aggregate Hi-C signal at TAD boundaries identified in DMSO-treated cells. **k-l**, P(s) plots (**k**) and derivatives (**l**), with arrows indicating cohesin loop signatures. **m**, Aggregate Hi-C signal at 8,334 loops[12]. **n**, Loop strength quantified along the loop-line (diagonal from bottom-left to top-right); blue and red indicate DMSO and TSA, respectively, with the bottom panel showing the TSA/DMSO ratio. **o**, Average intra-TAD interactions, significantly reduced upon TSA treatment (Two-tailed Wilcoxon rank-sum test, ***$p < 0.001$). **p**, TSA-induced reduction of intra-TAD interactions in the presence or absence of cohesin. **q**, Fold change of TSA-induced P(s) alterations with or without cohesin. **r-s**, P(s) plots from two replicates used for fold-change calculations (blue: DMSO; red: TSA). t-v, Second biological replicate of RAD21 ChIP-loop. **t**, Derivative plots showing global cohesin loop signals. **u**, CTCF-CTCF loop pileup and loop-line plots. **v**, Average intra-TAD interactions with statistical significance assessed by Wilcoxon rank-sum test (two-tailed, see Methods). All data were processed using Hi-C pipelines as described in Methods. Source data are provided.

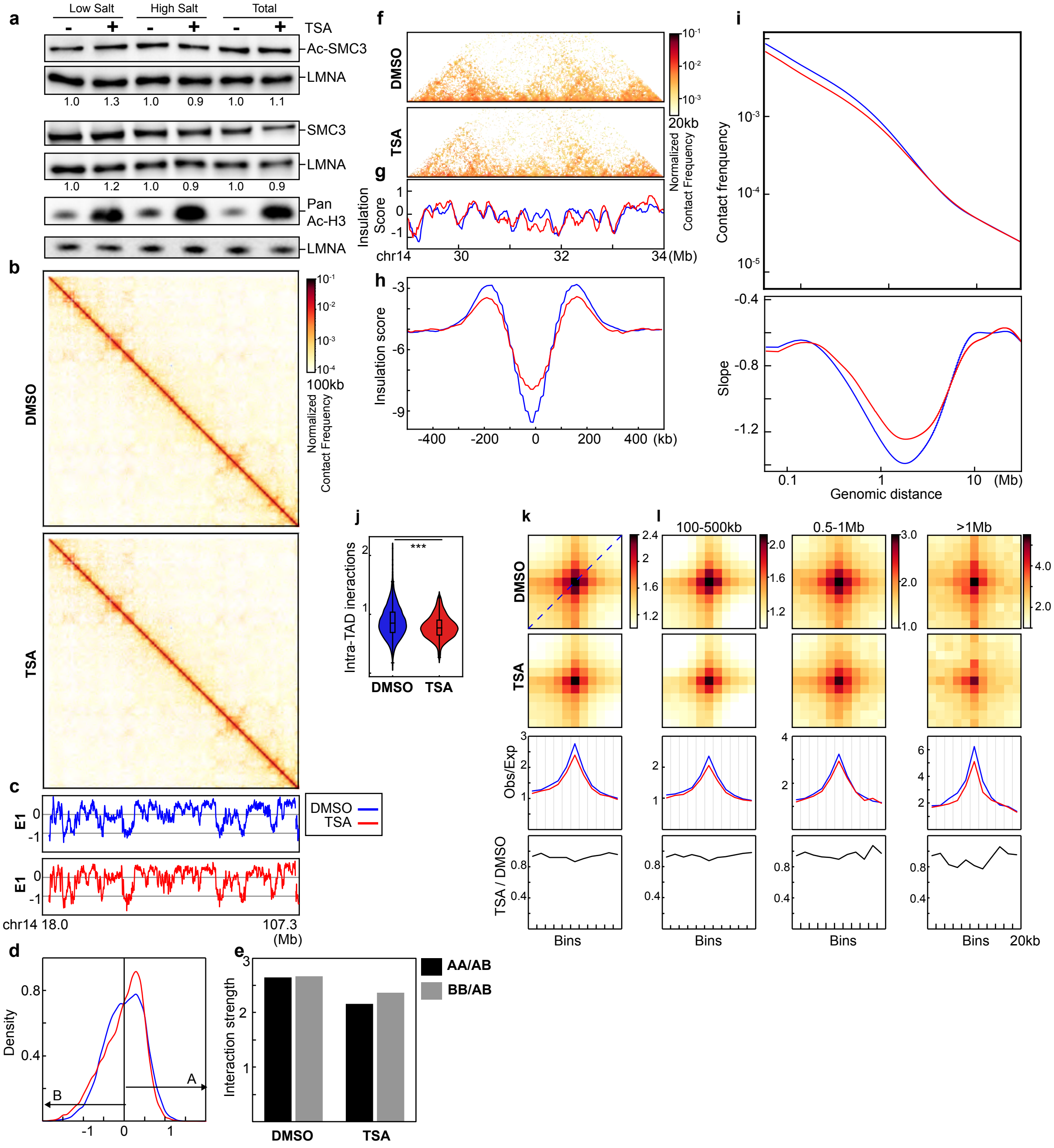
a
Low Salt
High Salt
Total
TSA
Ac-SMC3
LMNA
1.0 1.3 1.0 0.9 1.0 1.1
SMC3
LMNA
1.0 1.2 1.0 0.9 1.0 0.9
Pan Ac-H3
LMNA
b
DMSO
TSA
100kb
Normalized Contact Frequency
c
E1
DMSO
TSA
chr14 18.0
107.3 (Mb)
d
Density
e
Interaction strength
AA/AB
BB/AB
DMSO
TSA
f
20kb
g
Insulation Score
chr14
(Mb)
h
Insulation score
(kb)
i
Contact frenquency
Slope
Genomic distance
(Mb)
j
Intra-TAD ineractions
***
DMSO
TSA
k
l
100-500kb
0.5-1Mb
>1Mb
Obs/Exp
TSA / DMSO
Bins
20kb

Supplementary Fig. 2. Impact of histone hyperacetylation on wild-type HAP1 cells.
a, Western blot analysis of SMC3, acetylated SMC3, and acetylated histone H3 in HAP1-$RAD21^{TEV}$ nuclei under low- and high-salt conditions (100 mM and 200 mM NaCl) following 3h DMSO or TSA treatment. LMNA was used as a loading control. SMC3 and acetylated SMC3 levels were normalized to DMSO-treated nuclei. **b**, Hi-C interaction maps for HAP1 cells treated with DMSO or TSA (chromosome 14: 18.0-107.3 Mb). **c**, Eigenvector (E1) profiles across the same region. **d**, Distribution of E1 values, showing increased A-compartment signals after TSA treatment. **e**, Compartment interaction strength (A-A and B-B; see Methods). **f**, Hi-C maps for chromosome 14: 29-34 Mb. **g**, Insulation profiles for the same region. **h**, Aggregate Hi-C signal at TAD boundaries identified in DMSO-treated cells. **i**, P(s) plots (top) and derivatives (bottom), with arrows indicating cohesin loop signatures. **j**, Average intra-TAD interactions, significantly reduced upon TSA treatment (two-tailed Wilcoxon rank-sum test, ***$p < 0.0001$). **k**, Aggregate Hi-C signal at 8,334 loops[12], with loop strength quantified along the loop-line (diagonal from bottom-left to top-right). Blue and red indicate DMSO and TSA, respectively; the bottom panel shows the TSA/DMSO ratio. **l**, Averaged Hi-C signals for loops of different sizes, with corresponding loop-lines and differential loop-lines. In **c**, **d**, **f-l**, blue and red represent DMSO and TSA, respectively. Source data are provided.

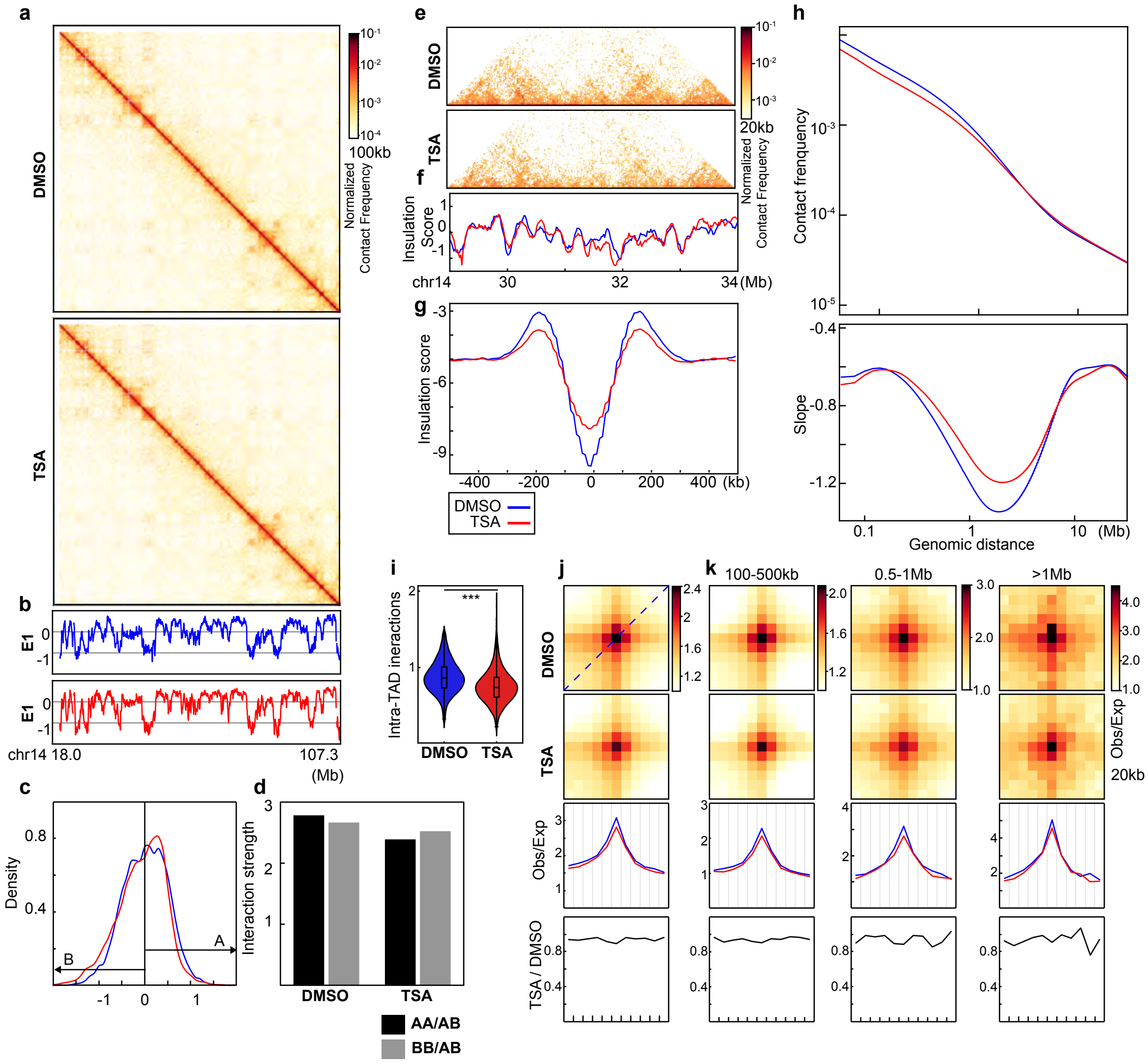

Supplementary Fig. 3. A replicate for impact of histone hyperacetylation on wild-type HAP1 cells. **a**, Hi-C interaction maps for HAP1 cells treated with/without TSA (top). Data are for the 18.0–107.3 Mb region of chromosome 14. **b**, Eigenvector E1 across the same region as in **a**. **c**, distribution of E1. The red arrow pointed the increased E1 bins after TSA treatment. **d**, Interaction strength of compartments. Dark and grey bars indicate the strength of the A-A and B-B interactions, respectively (see Methods). **e**, Hi-C interaction maps for HAP1 cells treated with/without TSA. Data for the 29–34 Mb region of chromosome 14 are shown. **f**, Insulation profiles for the same region as in **e**. **g**, Aggregate Hi-C data at TAD boundaries identified in the sample treated with DMSO. **h**, P(s) plots (top) and plots of their derivatives (bottom) for Hi-C data from cells treated with/without TSA. The arrow indicates the signature of cohesin loops. **i**, Average intra-TAD interactions across all TADs (see methods). Blue and red represent DMSO and TSA, respectively. Wilcoxon sum rank test, two-tailed, ***p<0.001. **j**, Aggregated Hi-C data at 8,334 loops identified in HAP1 cells according to[12] (upper heatmap). The average Hi-C signals from the bottom-left corner to the top-right corner of the respective loop aggregation heatmaps (top), as illustrated by the blue dashed line in the leftmost Hi-C panel in **j**. This line is defined as the loop-line. The blue and red loop-lines represent loop strength in DMSO and TSA treated samples, respectively. The dark line in the bottom plot indicates the ratio between TSA (red) and DMSO (blue) loop-lines. **k**, Averaged Hi-C signals at chromatin loops of three different loop sizes, and the associated loop-lines and differential loop lines as in **j**. **b, c, e-k**, The red and blue lines represent samples treated with and without TSA, respectively. Source data are provided as a Source Data file.

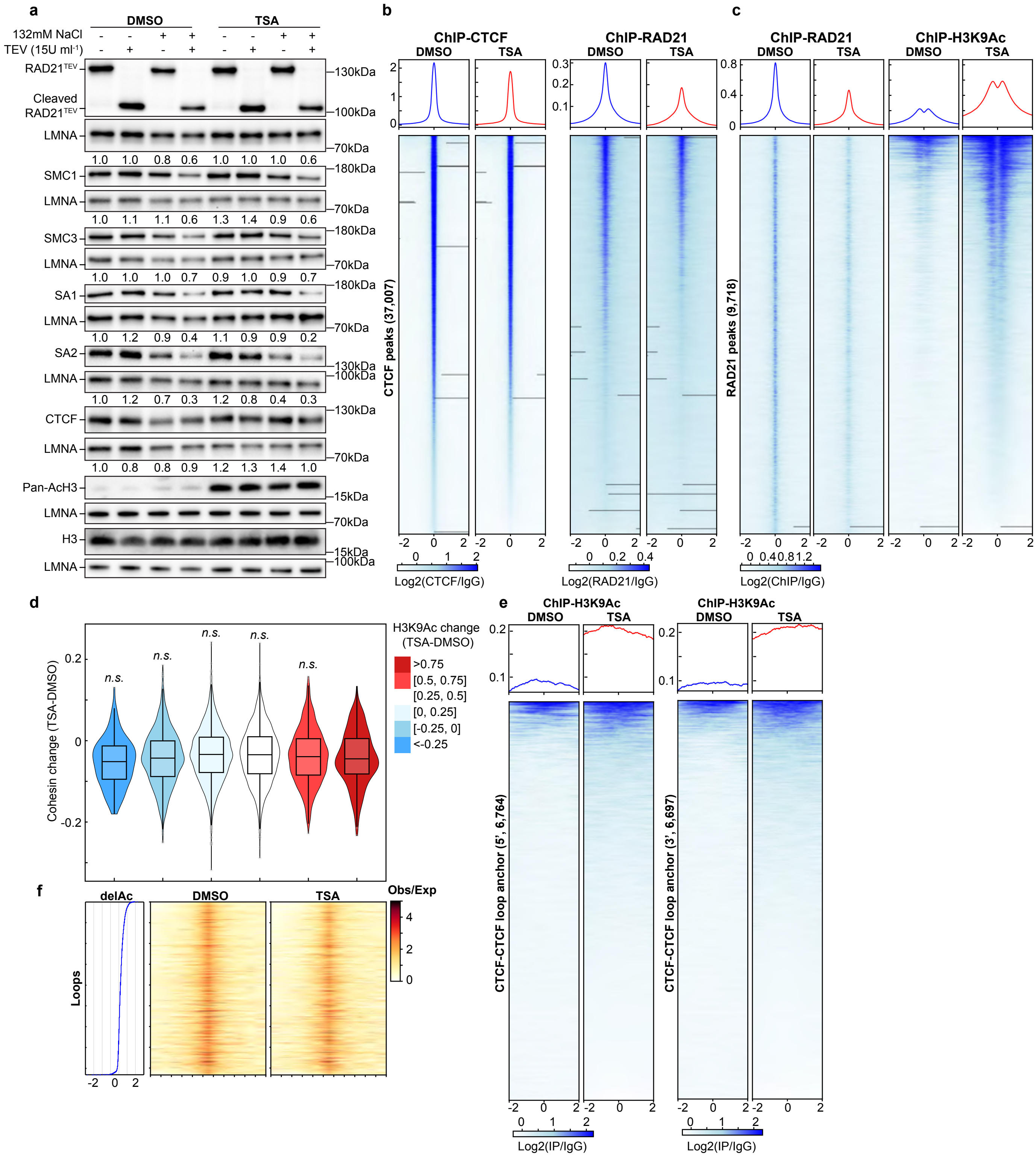

a
DMSO
TSA
132mM NaCl
TEV (15U ml-1)
RAD21TEV
Cleaved RAD21TEV
LMNA
SMC1
SMC3
SA1
SA2
CTCF
Pan-AcH3
H3
130kDa
100kDa
70kDa
180kDa
15kDa
1.0 1.0 0.8 0.6 1.0 1.0 1.0 0.6
1.0 1.1 1.1 0.6 1.3 1.4 0.9 0.6
1.0 1.0 1.0 0.7 0.9 1.0 0.9 0.7
1.0 1.2 0.9 0.4 1.1 0.9 0.9 0.2
1.0 1.2 0.7 0.3 1.2 0.8 0.4 0.3
1.0 0.8 0.8 0.9 1.2 1.3 1.4 1.0
b
ChIP-CTCF
ChIP-RAD21
DMSO
TSA
CTCF peaks (37,007)
Log2(CTCF/IgG)
Log2(RAD21/IgG)
c
ChIP-RAD21
ChIP-H3K9Ac
RAD21 peaks (9,718)
Log2(ChIP/IgG)
d
Cohesin change (TSA-DMSO)
n.s.
H3K9Ac change (TSA-DMSO)
>0.75
[0.5, 0.75]
[0.25, 0.5]
[0, 0.25]
[-0.25, 0]
<-0.25
e
ChIP-H3K9Ac
CTCF-CTCF loop anchor (5', 6,764)
CTCF-CTCF loop anchor (3', 6,697)
Log2(IP/IgG)
f
delAc
DMSO
TSA
Obs/Exp
Loops

Supplementary Fig. 4. A replicate for the **impact of histone hyperacetylation on chromatin binding of CTCF and cohesin, and CTCF-CTCF loop sites.**
a, A replicate of Western blot of chromatin retention of cohesin subunits in DMSO- or TSA-treated HAP1-RAD21^{TEV} nuclei in NB or NBS1 buffer (NB buffer + 132 mM NaCl) treated with/without TEV. LMNA was used as a loading control. Cohesin levels were normalized to DMOS-treated nuclei in NB buffer without TEV. Bottom panels show total histone H3 and acetylated histones by a pan-acetylated histone antibody. **b**, CTCF (left) and RAD21 (right) ChIP-seq signals at 37,007 CTCF binding sites identified in DMSO-treated cells. Top: average signal profile. Bottom: heatmaps across all sites. **c**, RAD21 (left) and H3K9Ac (right) ChIP-seq signals at 9,718 RAD21 binding sites. Top: average profiles. Bottom: heatmaps. **d**, Relationship between histone acetylation and RAD21 binding. RAD21 binding changes are plotted across bins of H3K9Ac signal changes (±2 kb from RAD21 peaks) using violin plots with embedded boxplots. Statistical significance was assessed using the two-tailed Wilcoxon rank-sum test. **e**, H3K9Ac ChIP-seq signals at 5′ and 3′ loop anchors (6,784 and 6,697 sites; 8,334 loops). **f**, Relationship between histone acetylation and loop strength. Left: ranked changes in combined H3K9Ac signal at loop anchors. Right: heatmaps of loop strength (loop-lines) under each condition. Color scale indicates contact frequency normalized by expected cis-interactions. ChIP-seq data in **c-f** are from replicate 2 (DMSO or TSA). n.s., not significant (two-tailed Wilcoxon rank-sum test). Source data are provided.

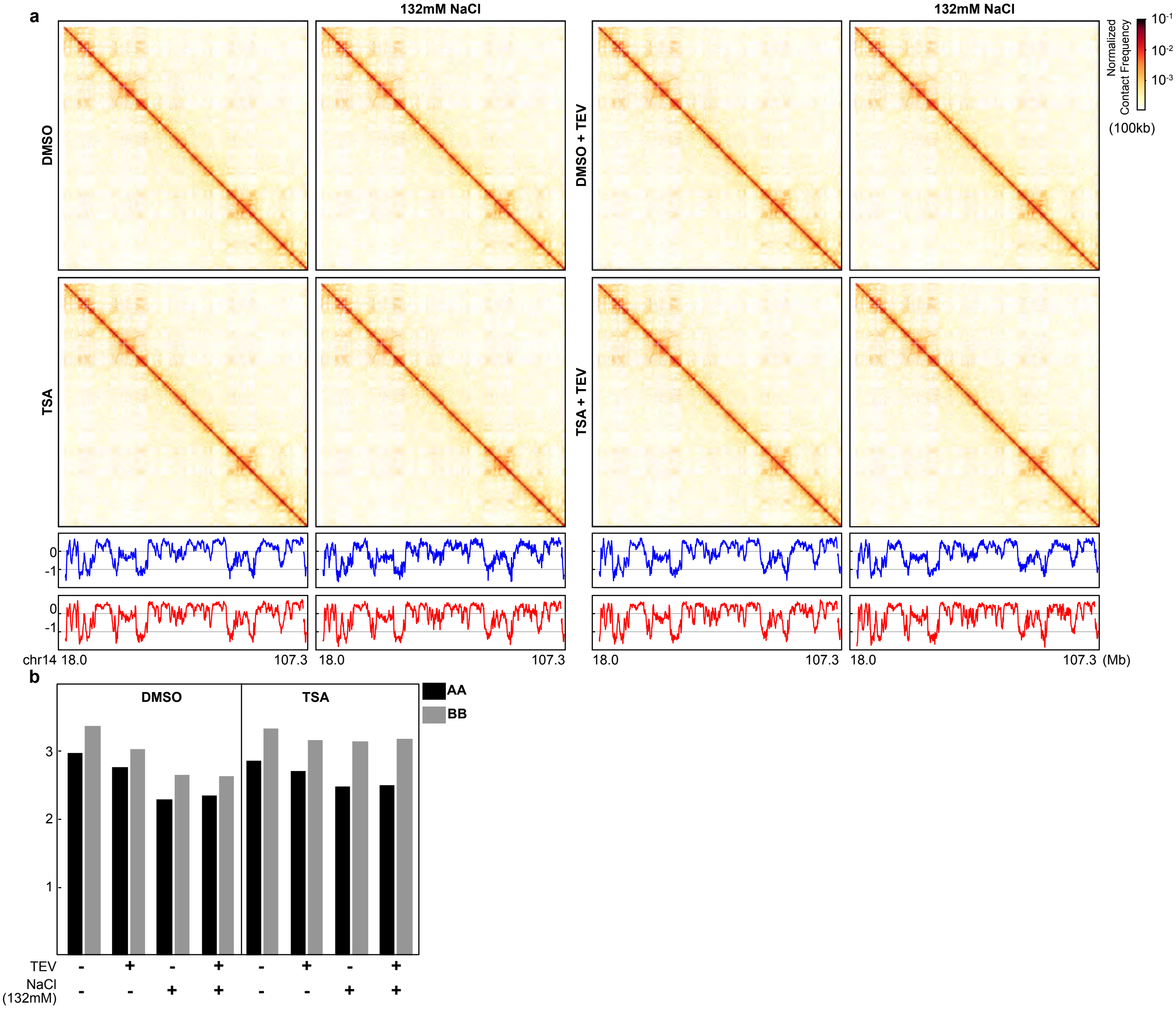

Supplementary Fig. 5. Impact of histone hyperacetylation on compartmentalization of purified nuclei. **a**, Hi-C interaction maps for DMSO- or TAS-treated HAP1-RAD21TEV nuclei treated with/without TEV in NB or NBS buffers. Data are for the 18.0–107.3 Mb region of chromosome 14. Plots with red or blue lines: Eigenvector E1 across the same region as in heatmaps. **b**, Interaction strength of compartments. Dark and grey bars indicate the strength of the A-A and B-B interactions, respectively (see Methods). Source data are provided as a Source Data file.

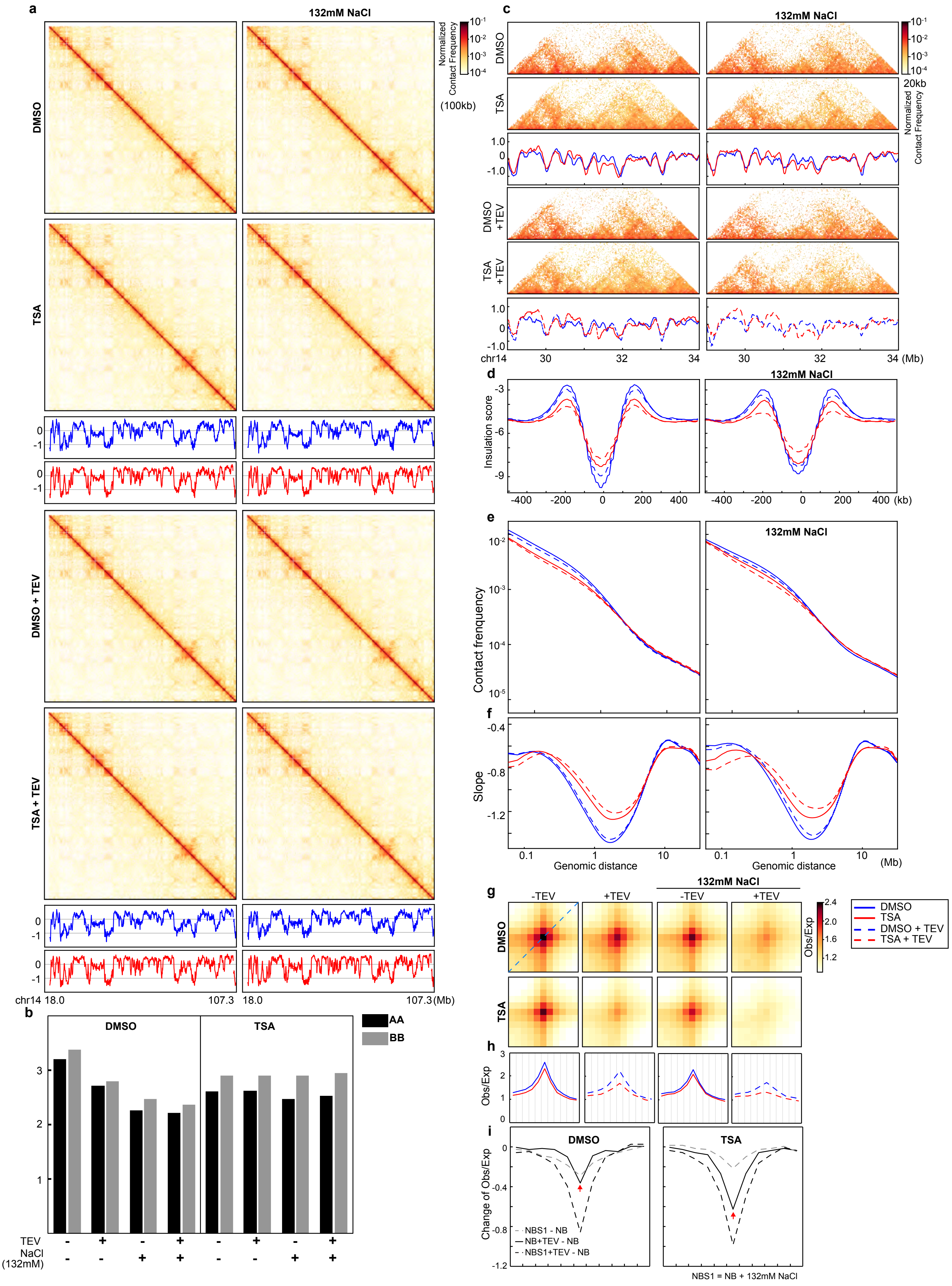

a
132mM NaCl
Normalized Contact Frequency
(100kb)
DMSO
TSA
DMSO + TEV
TSA + TEV
chr14 18.0
107.3
18.0
107.3 (Mb)
b
DMSO
TSA
AA
BB
TEV
NaCl (132mM)
c
132mM NaCl
DMSO
TSA
DMSO +TEV
TSA +TEV
20kb
Normalized Contact Frequency
chr14 30 32 34 (Mb)
d
132mM NaCl
Insulation score
-400 -200 0 200 400 (kb)
e
132mM NaCl
Contact frenquency
f
Slope
Genomic distance
(Mb)
g
132mM NaCl
-TEV
+TEV
Obs/Exp
DMSO
TSA
DMSO + TEV
TSA + TEV
h
Obs/Exp
i
DMSO
TSA
Change of Obs/Exp
NBS1 - NB
NB+TEV - NB
NBS1+TEV - NB
NBS1 = NB + 132mM NaCl

Supplementary Fig. 6. A replicate for histone hyperacetylation increases the sensitivity of cohesin at CTCF-CTCF loop anchors to RAD21 cleavage.

a, Hi-C interaction maps for DMSO- or TAS-treated HAP1-RAD21^{TEV} nuclei treated with/without TEV in NB or NBS buffers. Data are for the 18.0–107.3 Mb region of chromosome 14. Plots with red or blue lines: Eigenvector E1 across the same region as in heatmaps. **b,** Interaction strength of compartments. Dark and grey bars indicate the strength of the A-A and B-B interactions, respectively (see Methods). **c**, Examples of Hi-C maps obtained with DMSO- or TSA-treated HAP1-RAD21^{TEV} nuclei treated with or without TEV in NB (left) or NBS buffer (right). Insulation profiles (bottom) for the same region for each condition. **d**, Aggregate Hi-C data at TAD boundaries identified in DMSO- or TSA-treated nuclei treated with or without TEV in NB (left) or NBS1 (right) buffer. **e** and **f**, P(s) plots (top) and plots of their derivatives (bottom) for Hi-C data from DMSO- or TSA-treated nuclei with or without TEV treatment in NB (left) or NBS1 (right) buffers. The arrows indicate the signature of cohesin loops. **g**. Aggregate Hi-C data at loops identified in HAP1 cells (as in Fig. 1i; top). The average Hi-C signals from the bottom-left corner to the top-right corner of the respective loop-aggregated heatmaps (top), illustrated by the blue dashed line in the leftmost Hi-C panel in g, are shown (bottom). **h**, The differential loop lines (all other conditions versus NB buffer), left and right panels are DMSO versus TSA treatment. Source data are provided as a Source Data file.

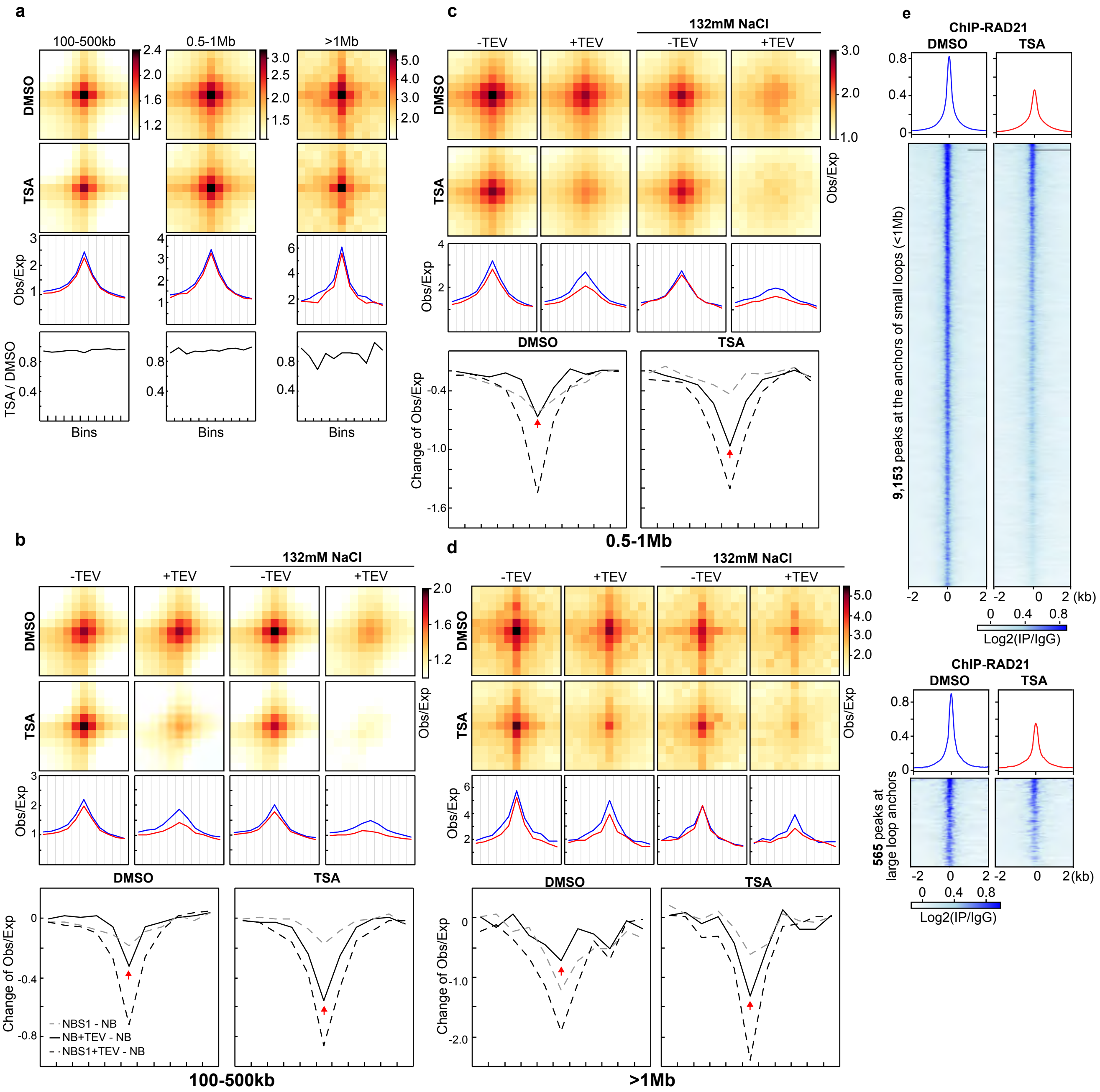

Supplementary Fig. 7. A replicate for CTCF-CTCF loops tolerate cohesin loss induced by histone hyperacetylation, regardless of loop sizes.

a, A replicate of aggregate Hi-C data at chromatin loops of different sizes identified in HAP1 cells (as in **Fig. 4a**). For each panel, two upper heatmaps: aggregate Hi-C data at chromatin loops of different sizes; middle: CTCF-CTCF loop lines at chromatin loops of different sizes; bottom: the ratio of loop lines (TSA versus DMSO). **b-d**, A replicate of aggregate Hi-C data at chromatin loops of different sizes identified in HAP1 cells (as in Fig. **4b-d**). For each panel, upper two heatmpas: aggregate Hi-C data at chromatin loops of different sizes; middle: loop lines at chromatin loops of different sizes; bottom: the differential loop lines (all other conditions versus NB buffer), left and right panels are DMSO versus TSA treatment. **e**, Average RAD21 ChIP–seq signals in each indicated condition for 565 binding sites that overlapped with the anchors of CTCF-CTCF large loops (1Mb, right) and the rest 9,153 binding sites (left). **a-e**, The red and blue lines represent samples treated with and without TSA, respectively. Source data are provided as a Source Data file.

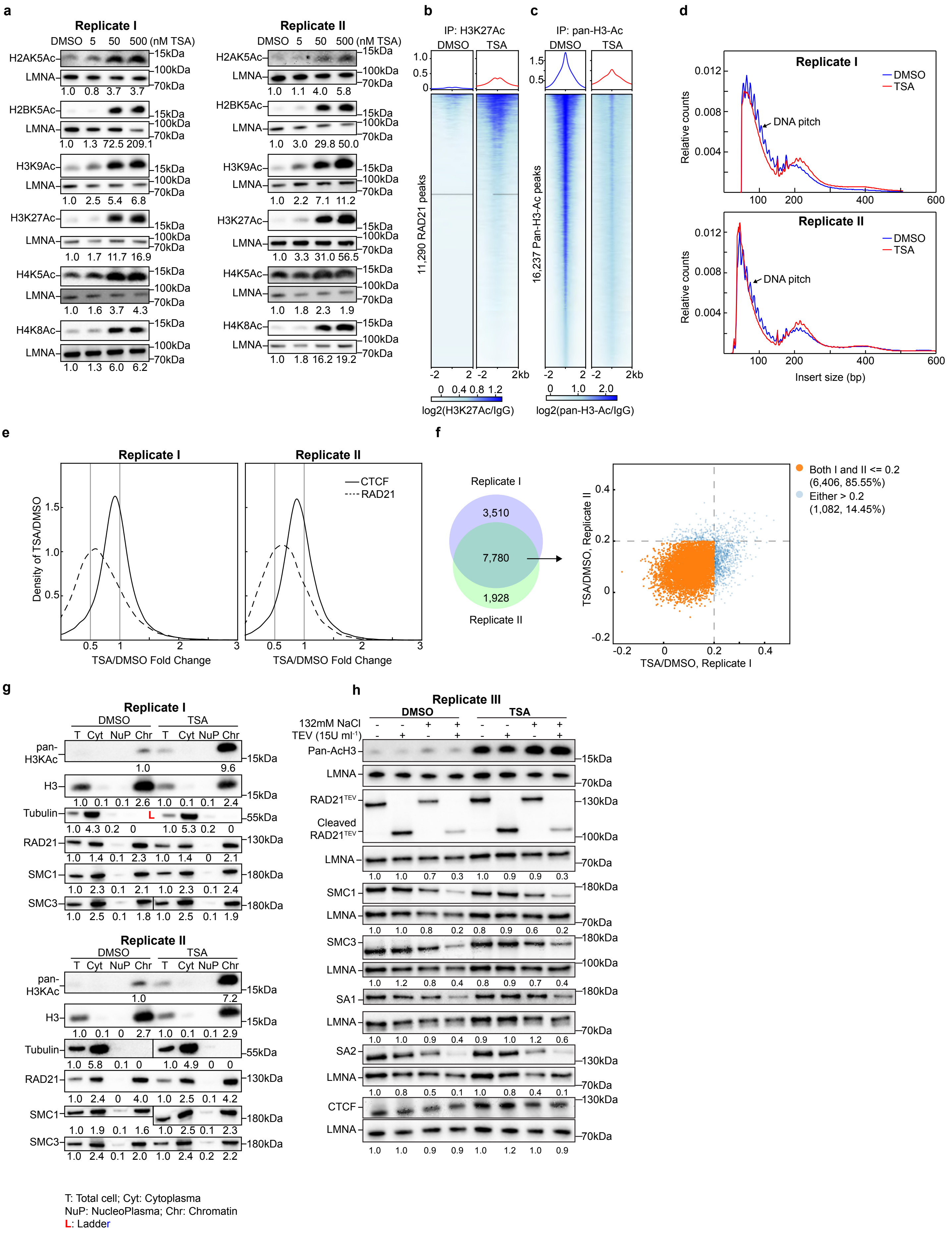

a
Replicate I
DMSO 5 50 500 (nM TSA)
H2AK5Ac
LMNA
1.0 0.8 3.7 3.7
H2BK5Ac
LMNA
1.0 1.3 72.5 209.1
H3K9Ac
LMNA
1.0 2.5 5.4 6.8
H3K27Ac
LMNA
1.0 1.7 11.7 16.9
H4K5Ac
LMNA
1.0 1.6 3.7 4.3
H4K8Ac
LMNA
1.0 1.3 6.0 6.2
15kDa
100kDa
70kDa
Replicate II
DMSO 5 50 500 (nM TSA)
1.0 1.1 4.0 5.8
1.0 3.0 29.8 50.0
1.0 2.2 7.1 11.2
1.0 3.3 31.0 56.5
1.0 1.8 2.3 1.9
1.0 1.8 16.2 19.2
b
IP: H3K27Ac
DMSO TSA
11,290 RAD21 peaks
-2 2 -2 2kb
0 0.4 0.8 1.2
log2(H3K27Ac/IgG)
c
IP: pan-H3-Ac
DMSO TSA
16,237 Pan-H3-Ac peaks
0 1.0 2.0
log2(pan-H3-Ac/IgG)
d
Replicate I
Replicate II
DMSO
TSA
DNA pitch
Relative counts
Insert size (bp)
e
Replicate I
Replicate II
CTCF
RAD21
Density of TSA/DMSO
TSA/DMSO Fold Change
f
Replicate I
3,510
7,780
1,928
Replicate II
Both I and II <= 0.2 (6,406, 85.55%)
Either > 0.2 (1,082, 14.45%)
TSA/DMSO, Replicate I
TSA/DMSO, Replicate II
g
Replicate I
DMSO TSA
T Cyt NuP Chr T Cyt NuP Chr
pan-H3KAc
1.0 9.6
H3
1.0 0.1 0.1 2.6 1.0 0.1 0.1 2.4
Tubulin
1.0 4.3 0.2 0 1.0 5.3 0.2 0
RAD21
1.0 1.4 0.1 2.3 1.0 1.4 0 2.1
SMC1
1.0 2.3 0.1 2.1 1.0 2.3 0.1 2.4
SMC3
1.0 2.5 0.1 1.8 1.0 2.5 0.1 1.9
15kDa
55kDa
130kDa
180kDa
Replicate II
DMSO TSA
T Cyt NuP Chr T Cyt NuP Chr
pan-H3KAc
1.0 7.2
H3
1.0 0.1 0 2.7 1.0 0.1 0.1 2.9
Tubulin
1.0 5.8 0.1 0 1.0 4.9 0 0
RAD21
1.0 2.4 0 4.0 1.0 2.5 0.1 4.2
SMC1
1.0 1.9 0.1 1.6 1.0 2.5 0.1 2.3
SMC3
1.0 2.4 0.1 2.0 1.0 2.4 0.2 2.2
T: Total cell; Cyt: Cytoplasma
NuP: NucleoPlasma; Chr: Chromatin
L: Ladder
h
Replicate III
DMSO TSA
132mM NaCl - - + + - - + +
TEV (15U ml-1) - + - + - + - +
Pan-AcH3
LMNA
RAD21TEV
Cleaved RAD21TEV
LMNA
1.0 1.0 0.7 0.3 1.0 0.9 0.9 0.3
SMC1
LMNA
1.0 1.0 0.8 0.2 0.8 0.9 0.6 0.2
SMC3
LMNA
1.0 1.2 0.8 0.4 0.8 0.9 0.7 0.4
SA1
LMNA
1.0 1.0 0.9 0.4 0.9 1.0 1.2 0.6
SA2
LMNA
1.0 0.8 0.5 0.1 1.0 0.8 0.4 0.1
CTCF
LMNA
1.0 1.0 0.9 0.9 1.0 1.2 1.0 0.9

Supplementary Fig. 8. Additional validation of histone acetylation, chromatin accessibility, and cohesin binding.
a, Western blot analysis (two biological replicates) of histone acetylation levels following DMSO or TSA treatment (5, 50, and 500 nM for 3 h), using antibodies against H2AK5Ac, H2BK5Ac, H3K9Ac, H3K27Ac, H4K5Ac, and H4K8Ac. **b**, Replicate H3K27Ac ChIP-seq signals at 11,290 RAD21 peaks identified from DMSO-treated cells (replicate 2). **c**, Replicate ChIP–seq analysis using a pan-H3-acetylation antibody, showing signals at 16,237 peaks identified in DMSO-treated cells. **d**, ATAC–seq fragment size distributions from two biological replicates of DMSO- and TSA-treated cells (blue: DMSO; red: TSA). ATAC-seq was performed using a Motif Active kit. **e**, Distribution of fold changes (TSA/DMSO) in CTCF and RAD21 ChIP-seq signals. Solid lines represent CTCF, and dashed lines represent RAD21. Left and right panels correspond to biological replicates 1 and 2, respectively. **f**, Reproducibility of TSA-induced RAD21 changes between replicates. Left: overlap of RAD21 peaks identified in DMSO-treated cells, yielding 7,488 shared peaks. Right: comparison of TSA/DMSO fold changes at these shared sites, demonstrating strong concordance. **g**, Cell fractionation analysis (two biological replicates) following DMSO or TSA treatment for 3 h. Total (T), cytoplasmic (Cyt), nucleoplasmic (NuP), and chromatin (Chr) fractions were analyzed for histone acetylation and chromatin-associated proteins (H3, RAD21, SMC1, SMC3, CTCF, and Tubulin) using specific antibodies. **h**, Replicate semi-in vitro chromatin-binding assay, as in Fig. 2**a-b** and Supplementary Fig. 4**a**. Source data are provided.

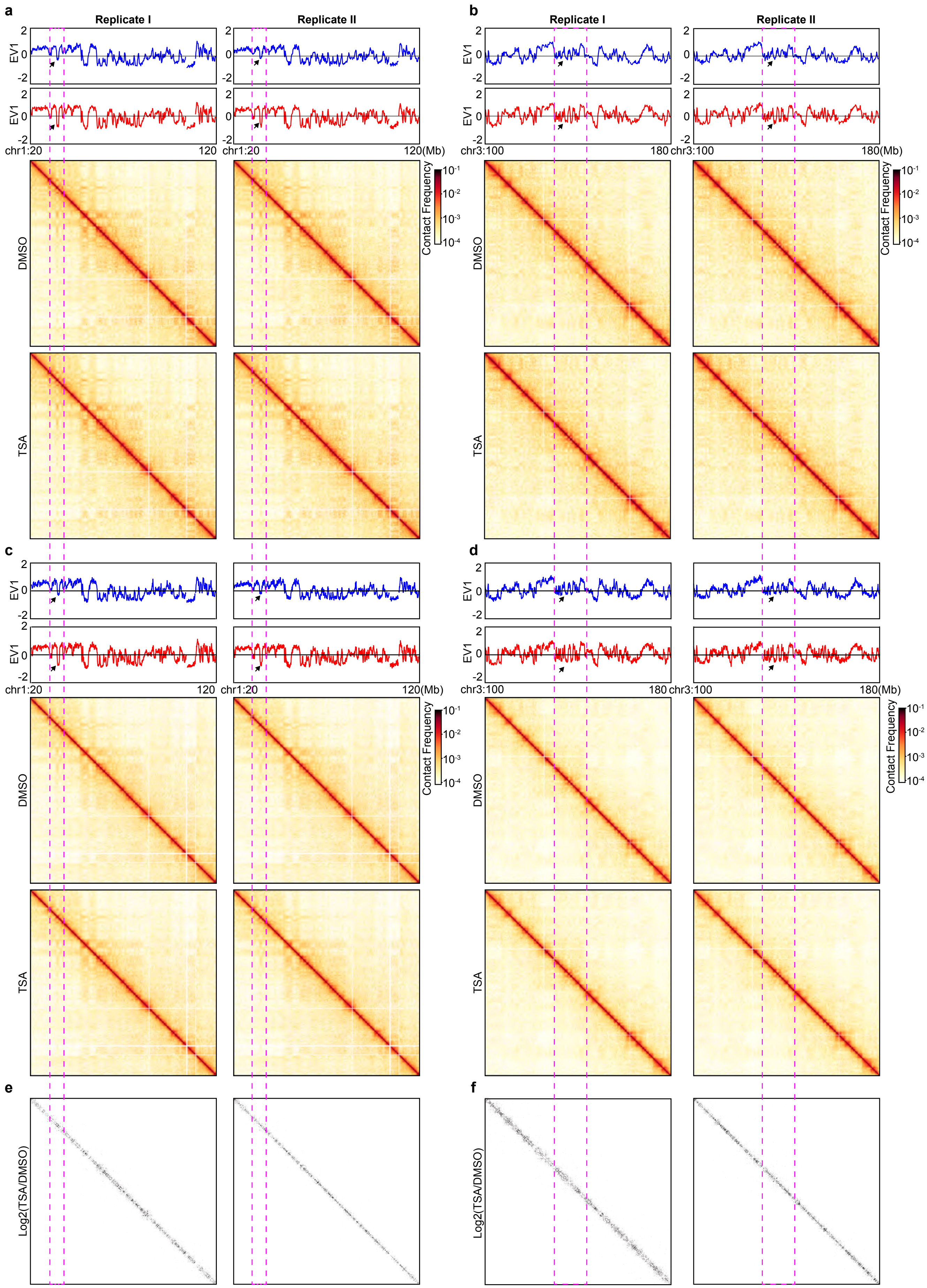
a
b
c
d
e
f
Replicate I
Replicate II
EV1
chr1:20
120
120(Mb)
chr3:100
180
180(Mb)
DMSO
TSA
Contact Frequency
10^{-1}
10^{-2}
10^{-3}
10^{-4}
Log2(TSA/DMSO)

Supplementary Fig. 9

a-b, Hi-C interaction maps for HAP1-RAD^{TEV} cells treated with DMSO or TSA. Shown are the 20-120 Mb region of chromosome 1 **(a)** and the 100-120 Mb region of chromosome 3 **(b)**. Top panels, eigenvector E1 (EV1) values across the same genomic regions. Dark arrows indicate loci exhibiting altered EV1 values. These regions were selected based on enriched EV1 changes or compartment switching. Bottom panels, normalized Hi-C contact maps. **c-d**, EV1 profiles and Hi-C contact maps for the same genomic regions shown in a and b, respectively, generated with increased sequencing depth. Samples in **c** and **d** were sequenced to **an additional ~200 million reads per sample**. **e-f**, Differential Hi-C contact maps showing fold changes (log_2[TSA/DMSO]) after observed/expected (Obs/Exp) normalization, plotted at 100-kb resolution using the deeper sequencing data shown in **c-d**. Source data are provided as a Source Data file.

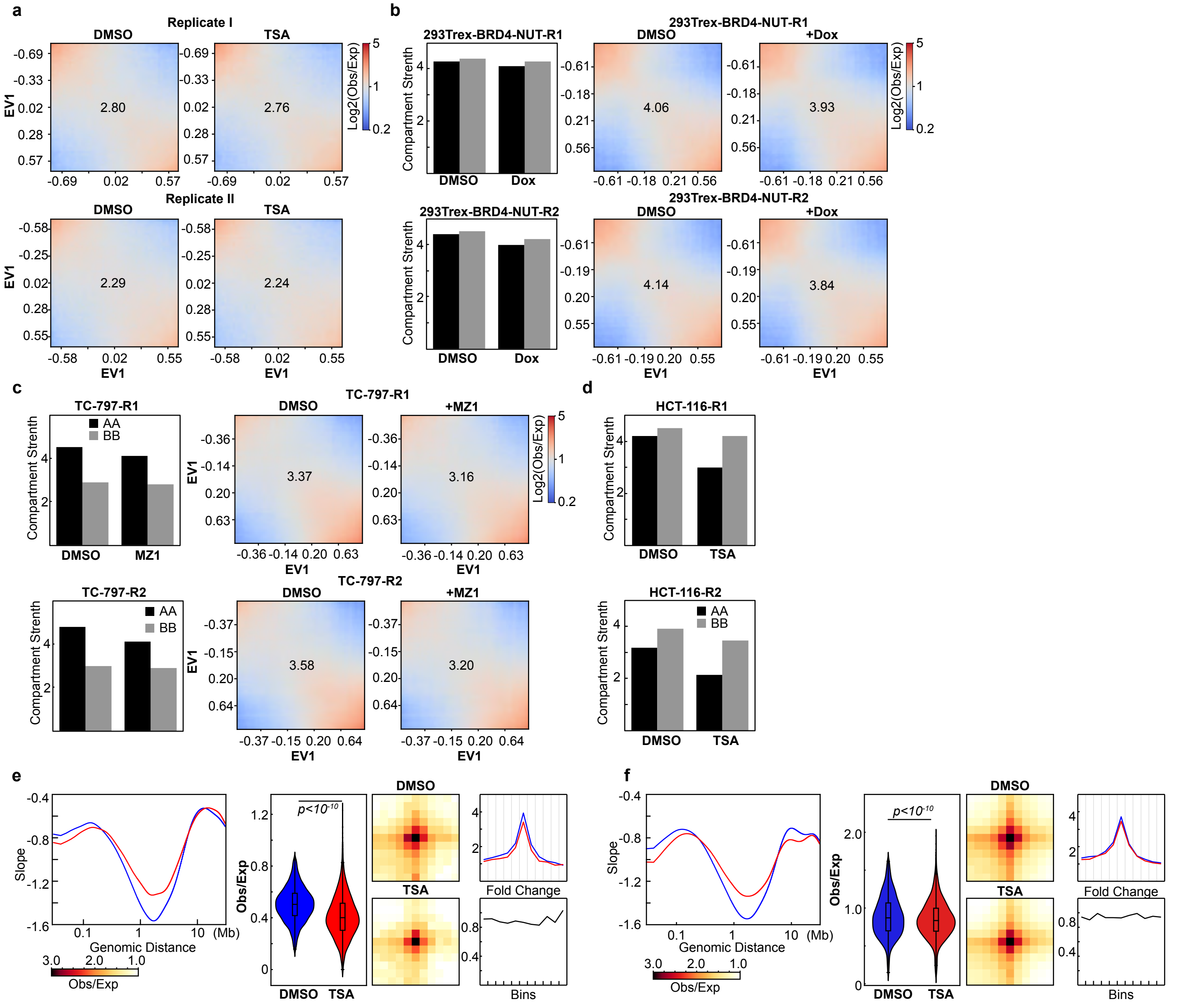

Supplementary Fig. 10

a, Saddle plots from two biological replicates of Hi-C analysis on DMSO- and TSA-treated HAP1-RAD21TEV cells. The number at the center of each panel indicates compartment strength, calculated as (AA+BB)/(AB+BA) using the Open2C pipeline, as described in the Methods. **b**, Compartment strength analysis from two biological replicates of DMSO- or Doxcicline-treated 293Trex-BRD4-NUT cells. The left column of panels shows AA and BB compartment strength, and the saddle plots indicate compartment strength. Raw Hi-C fastq data were obtained from the published dataset GSE133163. **c**, Compartment strength analysis from two biological replicates of DMSO- or MZ1-treated TC-797 cells. The left column of panels shows AA and BB compartment strength, and the saddle plots indicate compartment strength. Raw Hi-C fastq data were obtained from the published dataset GSE133165. **d**, Two biological replicates of AA and BB compartment strength from DMSO- or TSA- treated HCT-116 cells. AA and BB compartment strength was calculated as described in the Methods. **e-f**, Cohesin loop analysis using ChIP-loop and PLAC-seq methods. **e**, One replicate of ChIP-loop performed using an antibody recognizing the C-terminus of RAD21 (Abcam, ab992). **f**, One replicate of PLAC-seq performed using an antibody recognizing the C-terminus of RAD21. For each panel, all data were processed using the Hi-C analysis pipeline as described in Methods. Left, derivative plots showing global cohesin loop signals. Middle, average intra-TAD interactions, with statistical significance assessed using the two-tailed Wilcoxon rank-sum test (see Methods). Right, CTCF-CTCF loop pileup analysis and corresponding loop lin plots. Blue and red indicate DMSO and TSA treatment, respectively. Source data are provided as a Source Data file.

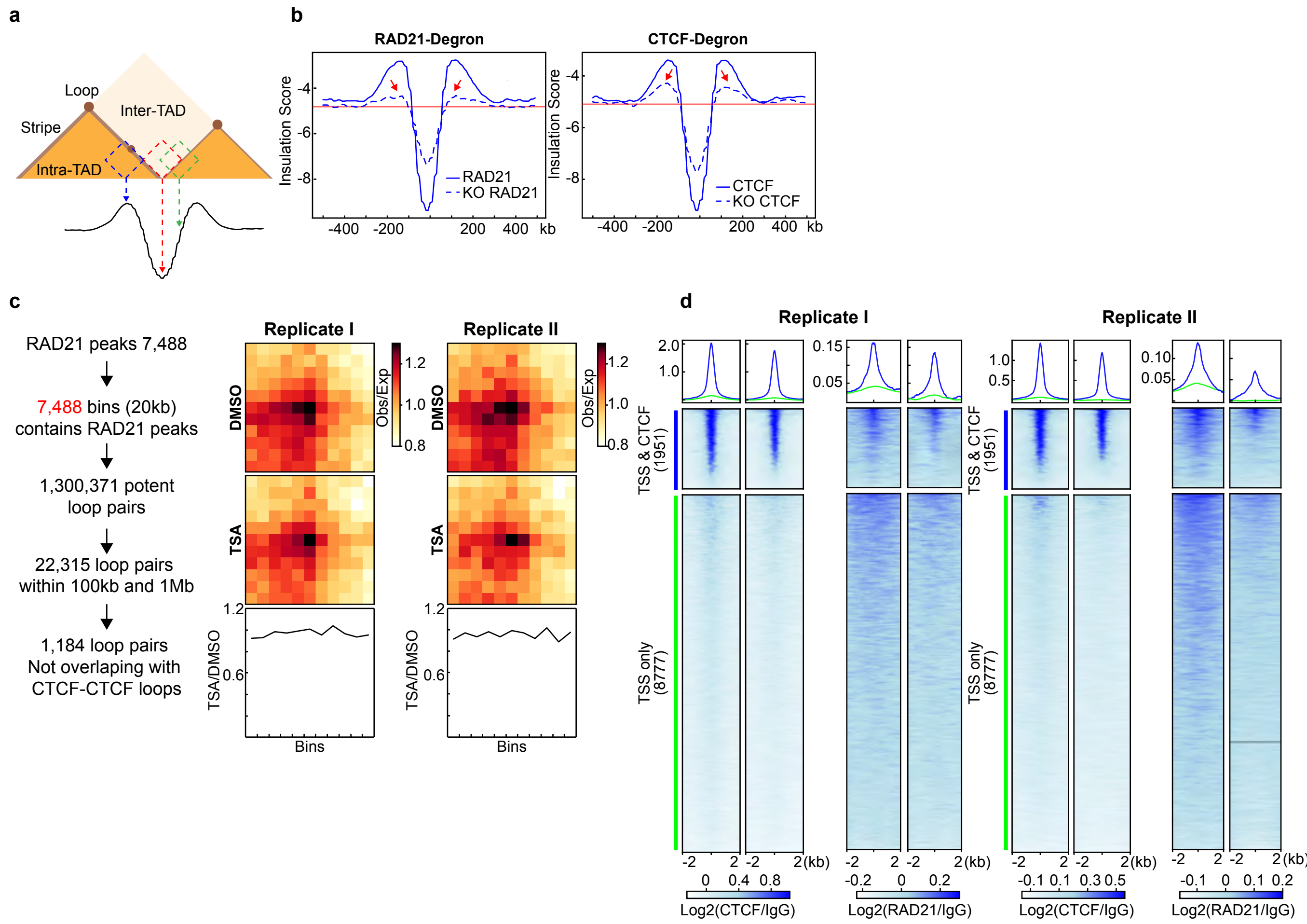

Supplementary Fig. 11.
a, A schematic cartoon illustrating the calculation of the insulation score and the interpretation of shoulder and valley signals in the boundary pile-up plot. **b**, Impact of cohesin (upper panel) and CTCF (lower panel) depletion on shoulder and valley signals in TAD boundary pile-up plots. Blue solid and dashed lines indicate conditions without and with depletion, respectively. TAD boundaries were identified in samples without RAD21 or CTCF depletion. **c**, An integrated analysis to identify cohesin loops that do not overlap with CTCF–CTCF loops. The left panel illustrates the analysis strategy, and the right panels show pile-up analyses of Hi-C contacts at these cohesin loops. The bottom-right panels indicate fold changes in loop signal between DMSO- and TSA-treated cells. **d**, Profiles of CTCF and RAD21 ChIP-seq signals at active transcription start sites (TSSs) in cells treated with DMSO or TSA are shown. Of the 13,412 active TSSs in HAP1 cells, 1,951 overlap with CTCF binding sites, whereas 8,777 do not overlap with CTCF binding sites (defined as >2 kb away from CTCF sites). Both the average ChIP-seq signal profiles (upper panels) and heatmaps of CTCF and RAD21 ChIP-seq signals for these two groups of TSSs are shown. The left and right panels correspond to two biological replicates. Active TSSs were defined as described in Liu and Dekker, Nature Cell Biology, 2022. Source data are provided as a Source Data file.

Unprocessed blots for Supplementary Fig. 1a

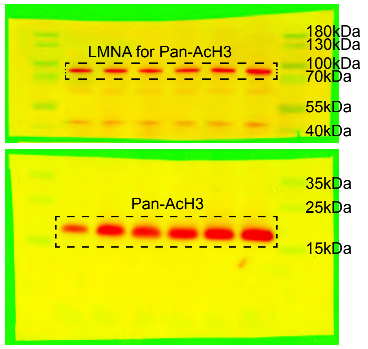

Unprocessed blots for Supplementary Fig. 1b

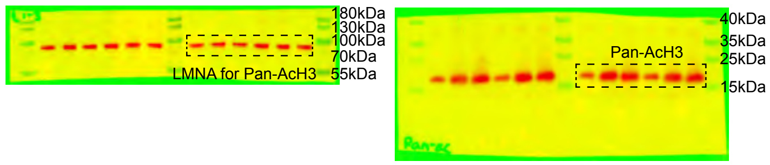

Unprocessed blots for Supplementary Fig. 2a

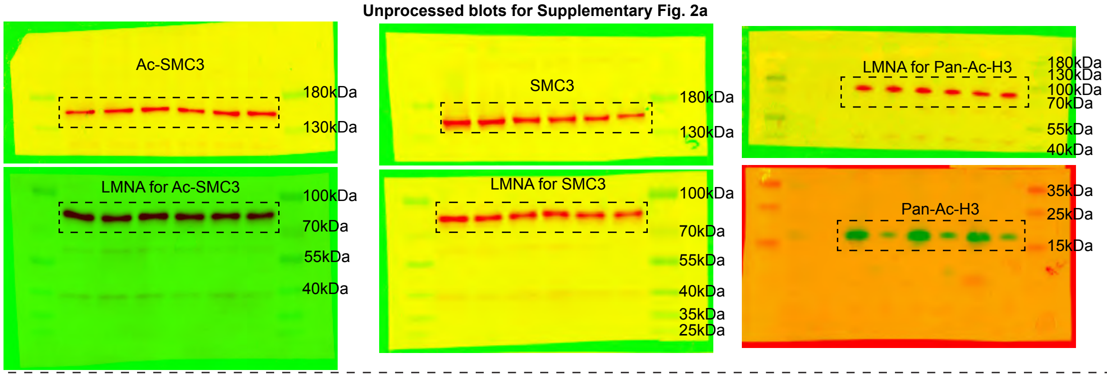

Unprocessed blots for Supplementary Fig. 4a

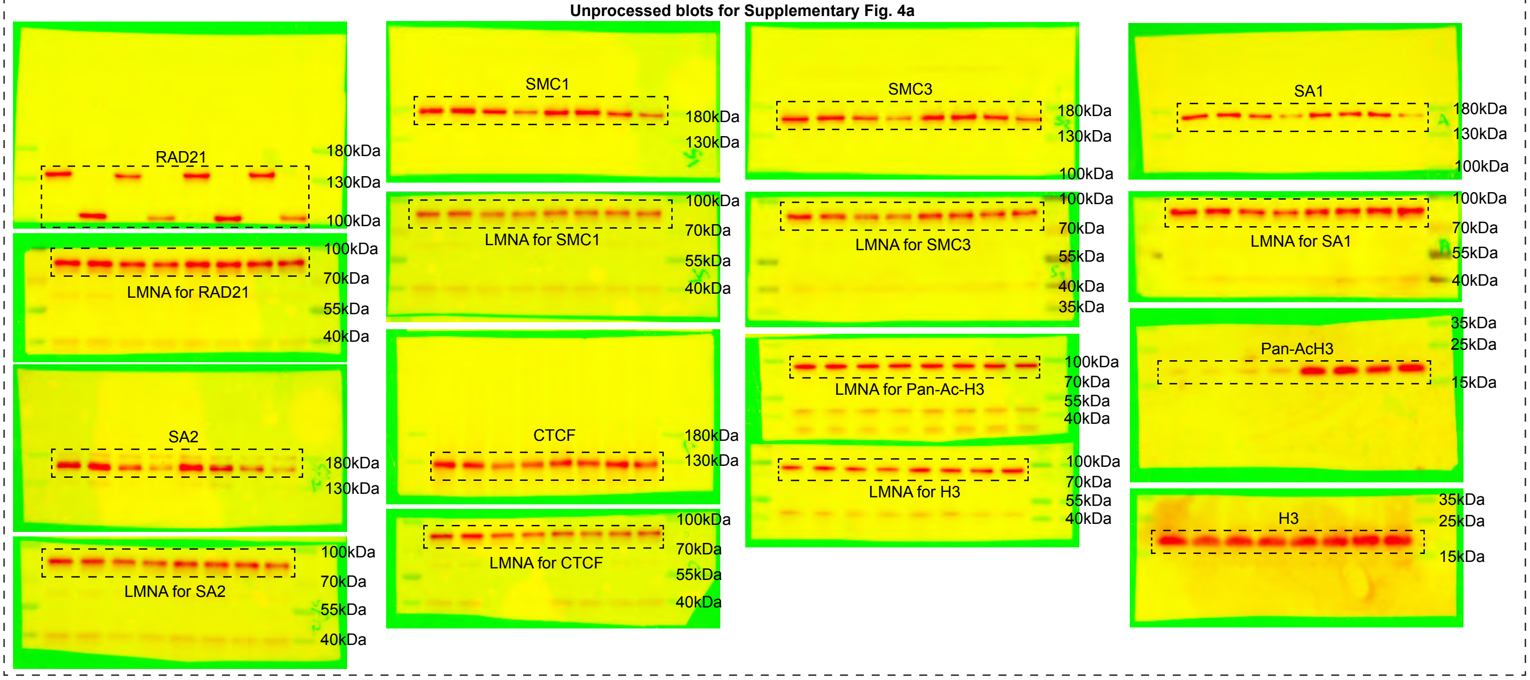

Unprocessed blots for Supplementary Fig. 11a

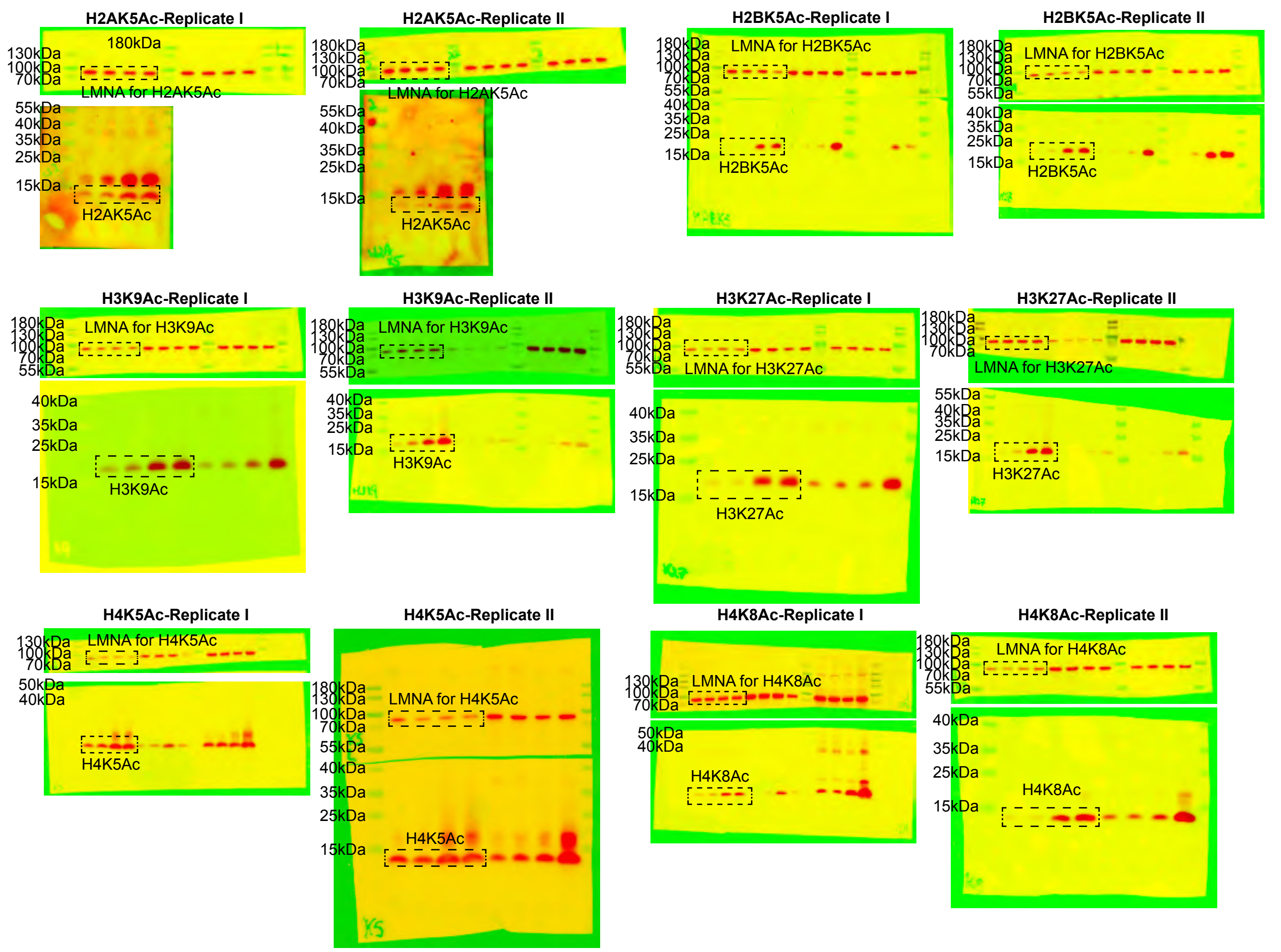

Unprocessed blots for Supplementary Fig. 11g

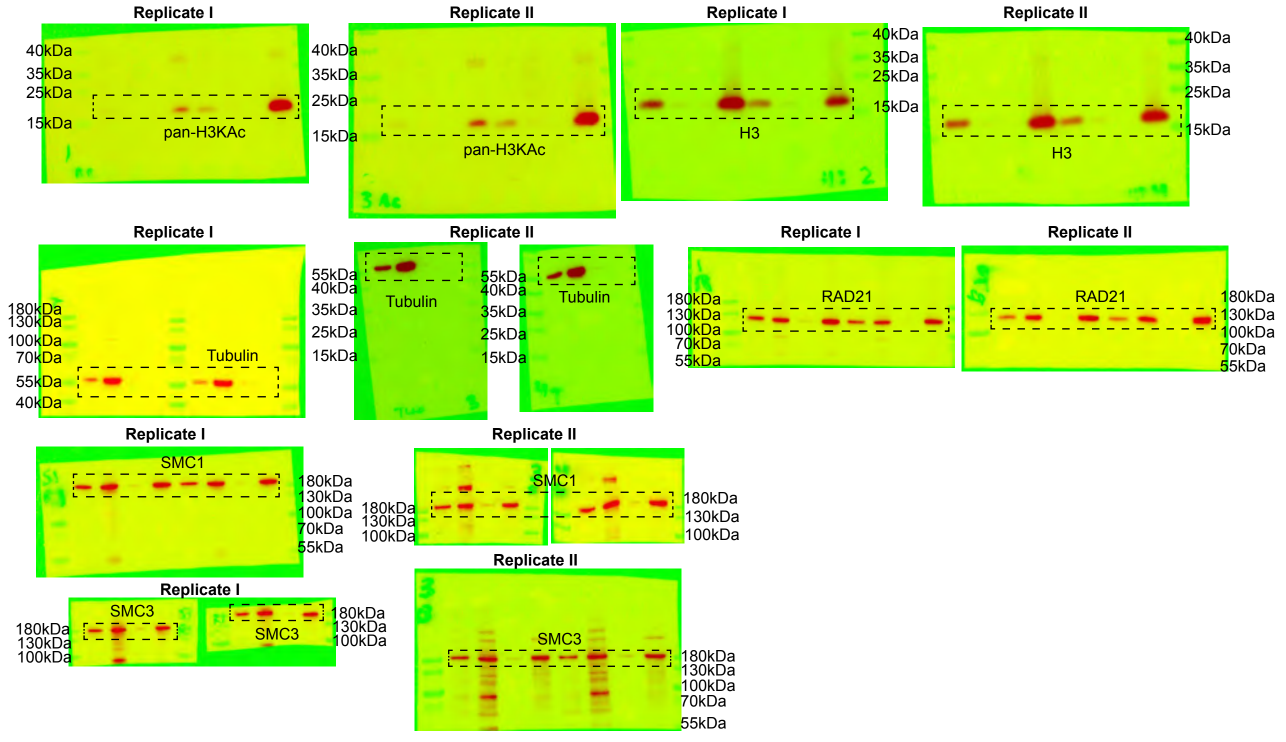

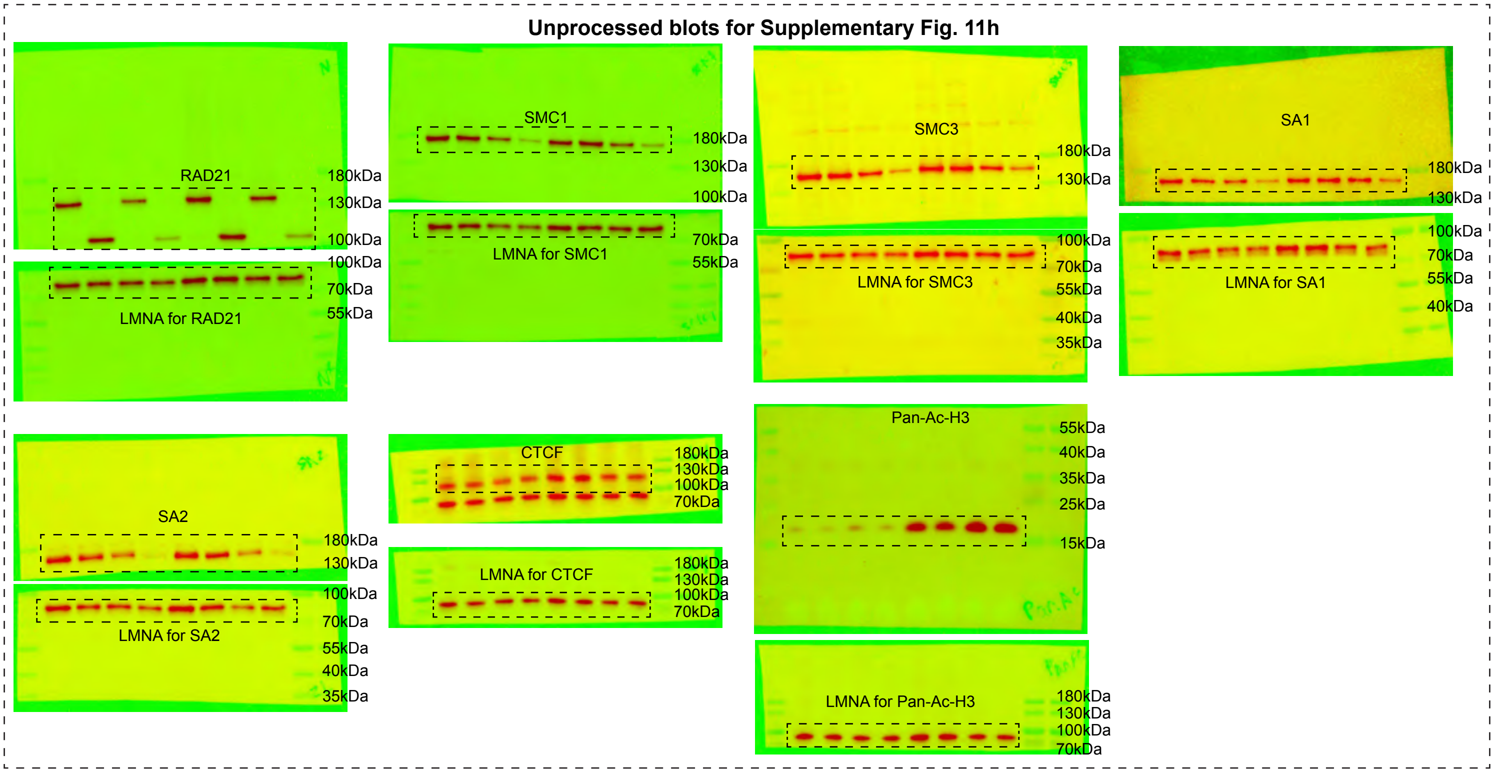

Unprocessed blots for Supplementary Fig. 11h
RAD21
180kDa
130kDa
100kDa
100kDa
70kDa
55kDa
LMNA for RAD21
SMC1
180kDa
130kDa
100kDa
70kDa
55kDa
LMNA for SMC1
SMC3
180kDa
130kDa
100kDa
70kDa
55kDa
40kDa
35kDa
LMNA for SMC3
SA1
180kDa
130kDa
100kDa
70kDa
55kDa
40kDa
LMNA for SA1
SA2
180kDa
130kDa
100kDa
70kDa
55kDa
40kDa
35kDa
LMNA for SA2
CTCF
180kDa
130kDa
100kDa
70kDa
LMNA for CTCF
180kDa
130kDa
100kDa
70kDa
Pan-Ac-H3
55kDa
40kDa
35kDa
25kDa
15kDa
LMNA for Pan-Ac-H3
180kDa
130kDa
100kDa
70kDa
