# Supplementary material for: Histone Acetylation Differentially Modulates CTCF-CTCF Loops and Intra-TAD Interactions
Source: Nat Commun. 2026 Jul 20;17:6676. doi: 10.1038/s41467-026-75818-8 (PMC13385825; doi:10.1038/s41467-026-75818-8)
Supplement: Supplementary file 2 — Reporting Summary [file 41467_2026_75818_MOESM2_ESM.pdf]

nature portfolio

Corresponding author(s): Yu Liu and Job Dekker

Last updated by author(s): Feb 26, 2026

Reporting Summary

Nature Portfolio wishes to improve the reproducibility of the work that we publish. This form provides structure for consistency and transparency in reporting. For further information on Nature Portfolio policies, see our Editorial Policies and the Editorial Policy Checklist.

Statistics

For all statistical analyses, confirm that the following items are present in the figure legend, table legend, main text, or Methods section.

n/a	Confirmed	
☐	☒	The exact sample size (*n*) for each experimental group/condition, given as a discrete number and unit of measurement
☒	☐	A statement on whether measurements were taken from distinct samples or whether the same sample was measured repeatedly
☐	☒	The statistical test(s) used AND whether they are one- or two-sided<br>*Only common tests should be described solely by name; describe more complex techniques in the Methods section.*
☒	☐	A description of all covariates tested
☒	☐	A description of any assumptions or corrections, such as tests of normality and adjustment for multiple comparisons
☐	☒	A full description of the statistical parameters including central tendency (e.g. means) or other basic estimates (e.g. regression coefficient) AND variation (e.g. standard deviation) or associated estimates of uncertainty (e.g. confidence intervals)
☐	☒	For null hypothesis testing, the test statistic (e.g. *F*, *t*, *r*) with confidence intervals, effect sizes, degrees of freedom and *P* value noted<br>*Give P values as exact values whenever suitable.*
☒	☐	For Bayesian analysis, information on the choice of priors and Markov chain Monte Carlo settings
☒	☐	For hierarchical and complex designs, identification of the appropriate level for tests and full reporting of outcomes
☒	☐	Estimates of effect sizes (e.g. Cohen's *d*, Pearson's *r*), indicating how they were calculated

Our web collection on statistics for biologists contains articles on many of the points above.

Software and code

Policy information about availability of computer code

Data collection	Hi-C and ChIPseq sequencing data was collected using HCS v3.4.0
Data analysis	Hi-C fastq files were mapped to hg19 human reference genome using distiller -nf mapping pipeline (http://github.com/mirnylab/distiller-nf). Binning and balancing was done according to Abdennur & Mirny, Bioinformatics 2020 and Imakaev et al., Nature Methods 2012. Downstream analyses were done using cooltools 0.3.0 (https://github.com/mirnylab/cooltools). ChIPseq fastq files were mapped onto hg19 human reference genome using Bowtie 2(Langmead et al., Genome Biology 2009). The subsequent analysis was done using HOMER 4.6 (Heinz et al. Mol Cell 2010) and Deeptools 3.0.2 (Ramirez et al. Nucleic Acids Research 2016). Cell cycle analysis of flow cytometry data was done using FlowJo v10. Western blot images were analyzed using Image Lab 6.1.0 Builder 7.

For manuscripts utilizing custom algorithms or software that are central to the research but not yet described in published literature, software must be made available to editors and reviewers. We strongly encourage code deposition in a community repository (e.g. GitHub). See the Nature Portfolio guidelines for submitting code & software for further information.

Data

Policy information about availability of data

All manuscripts must include a data availability statement. This statement should provide the following information, where applicable:

- Accession codes, unique identifiers, or web links for publicly available datasets
- A description of any restrictions on data availability
- For clinical datasets or third party data, please ensure that the statement adheres to our policy

Both raw and processed data generated in this study have been deposited in the GEO repository under accession GSE288762.

Research involving human participants, their data, or biological material

Policy information about studies with human participants or human data. See also policy information about sex, gender (identity/presentation), and sexual orientation and race, ethnicity and racism.

Reporting on sex and gender	na
Reporting on race, ethnicity, or other socially relevant groupings	na
Population characteristics	na
Recruitment	na
Ethics oversight	na

Note that full information on the approval of the study protocol must also be provided in the manuscript.

Field-specific reporting

Please select the one below that is the best fit for your research. If you are not sure, read the appropriate sections before making your selection.

[x] Life sciences [ ] Behavioural & social sciences [ ] Ecological, evolutionary & environmental sciences

For a reference copy of the document with all sections, see nature.com/documents/nr-reporting-summary-flat.pdf

Life sciences study design

All studies must disclose on these points even when the disclosure is negative.

Sample size	No statistical methods were used to predetermine sample size. The use of two replicates is common practice in molecular biology and genomics, given the cost and statistics are needed to be well balanced. (PMID: 25317452)
Data exclusions	No data were excluded from these analyses since all the data generated in this study passed quality control.
Replication	All experiments have at least two independent biological replicates. All findings described in the manuscript were confirmed in all individual replicates.
Randomization	Randomization of this study was not necessary as we did not allocate datasets into experimental groups.
Blinding	All analyses did not require blinding because results were directly linked with the data and this is neither a clinical study with large cohorts nor a genetics study with large numbers of samples.

Reporting for specific materials, systems and methods

We require information from authors about some types of materials, experimental systems and methods used in many studies. Here, indicate whether each material, system or method listed is relevant to your study. If you are not sure if a list item applies to your research, read the appropriate section before selecting a response.

Materials & experimental systems

n/a	Involved in the study
☐	☒ Antibodies
☐	☒ Eukaryotic cell lines
☒	☐ Palaeontology and archaeology
☒	☐ Animals and other organisms
☒	☐ Clinical data
☒	☐ Dual use research of concern
☒	☐ Plants

Methods

n/a	Involved in the study
☐	☒ ChIP-seq
☐	☒ Flow cytometry
☒	☐ MRI-based neuroimaging

Antibodies

Antibodies used

The following antibodies were used for Western blot: Anti-RAD21(Abcam ab154769, lot#GR3224138-28, 1:2000, recognizing N-terminus); Anti-RAD21 (Abcam ab992, lot#GR3310168-15, 1:2000, recognizing C-terminus); Anti-Lamin A/C (Abcam ab26300, lot# 1046291-5, 1:2000); Anti-SMC3 (Abcam ab9263, lot 1003294-8), 1:2000); Anti-SMC1 (Bethyl lab A300-055A, lot#6, 1:2000); Anti-CTCF (CST #3418L, lot#6, 1:2000); Anti-Pan-Acetylated-H3 (Abcam ab4791, lot#GR3454335-1), Anti-Acetylated SMC3(Millipore, lot# 3316674); Anti-SA1 (Bethyl lab A302-579A, lot#1); Anti-SA2(Bethyl lab A302-580A, lot#1); Anti-H3(Abcam ab1791, lot #GR8237728-1). H2AK5Ac (CST, #2576S, lot#3); H2BK5Ac (CST, #12799S, lot#1); Beta Tubulin (Abcam ab6046, lot# GR3243627-1); H3K9Ac (CST, #9649, lot#13); H3K27Ac (CST, #8173, lot#1); H4K5Ac (CST, #8647, lot#1); H4K8Ac (CST, #2594S, lot#11);Anti-rabbit IgG, HRP-linked (Cell Signaling #7074, 1:5000); Anti-Mouse IgG, HRP-linked (Cell Signaling, #7076,1:5000)
Antibody for ChIP, the same antibodies for CTCF and RAD21(C) were used for Western;H3K9Ac (Abcam ab4441, lot#1069505-3); H3K27Ac (Abcam, ab4729, lot#1086546-1); Rabbit IgG is from Sigma, I5005-10mg, Lot#SLCB7084.

Validation

All primary antibodies were shown to react with the appropriate human proteins on the manufacture's websites using Western blotting or other assays. All the Western results showed the target protein in predicted molecular weights.

Eukaryotic cell lines

Policy information about cell lines and Sex and Gender in Research

Cell line source(s)

HCT116-RAD21-mAID, degron2, a gift from Dr. Kanemaki's lab (Yesbolatova, et al Nat Commun, 2020); HAP1 parental cell line, purchased from Horizon Discoery (C859)

Authentication

For HCT116-RAD21-mAC, authentication was performed by detecting RAD21 using Western Blot after 5'-Ph-IAA treatments, which showed degradation of RAD21. For HAP1 cells, authentication was performed by inspection of Hi-C data obtained from HAP1 nuclei and detection of the appropriate translocations known for this cell line.

Mycoplasma contamination

Cell lines were tested for mycoplasma and found to be negative.

Commonly misidentified lines
(See ICLAC register)

No commonly misidentified cell lines were used in this study.

Plants

Seed stocks

na

Novel plant genotypes

na

Authentication

na

ChIP-seq

Data deposition

☒ Confirm that both raw and final processed data have been deposited in a public database such as GEO.

☒ Confirm that you have deposited or provided access to graph files (e.g. BED files) for the called peaks.

Data access links
May remain private before publication.

To review GEO accession GSE288762
https://www.ncbi.nlm.nih.gov/geo/query/acc.cgi?acc=GSE288762
qxmzuwgapnwtpsv

Files in database submission

Raw fastq files as below:

Replicate 1:
DMSO-IgG-R1: AS61_R1, AS61_R2
DMSO-CTCF-R1: AS62_R1, AS62_R2
DMSO-RAD21-R1: AS63_R1, AS63_R2
TSA-IgG-R1: AS65_R1, AS65_R2
TSA-CTCF-R1: AS66_R1, AS66_R2
TSA-RAD21-R1: AS67_R1, AS67_R2
DMSO-IgG-R4: AS167_R1, AS167_R2
DMSO-H3K9Ac-R4: AS168_R1, AS168_R2
TSA-IgG-R4: AS170_R1, AS170_R2
TSA-H3K9Ac-R4: AS171_R1, AS171_R2

Replicate 2
DMSO-IgG-R2: AS69_R1, AS69_R2
DMSO-CTCF-R2: AS70_R1, AS70_R2
DMSO-RAD21-R2: AS71_R1, AS71_R2
TSA-IgG-R2: AS73_R1, AS73_R2
TSA-CTCF-R2: AS74_R1, AS74_R2
TSA-RAD21-R2: AS75_R1, AS75_R2
DMSO-IgG-R3: AS161_R1, AS161_R2
DMSO-H3K9Ac-R3: AS162_R1, AS162_R2
TSA-IgG-R3: AS164_R1 AS164_R2
TSA-H3K9Ac-R3: AS165_R1, AS165_R2

Processed Files:
Bigwig files (Log2(CTCF or RAD21 or H3K9Ac)/IgG):
Replicate 1:
AS61.bw
AS62.bw
AS63.bw
AS65.bw
AS66.bw
AS67.bw
AS167.bw
AS168.bw
AS170.bw
AS171.bw

Replicate 2:
AS69.bw
AS70.bw
AS71.bw
AS73.bw
AS74.bw
AS75.bw
AS161.bw
AS162.bw
AS164.bw
AS165.bw

Genome browser session
(e.g. UCSC)

https://genome.ucsc.edu/cgi-bin/hgTracks?db=hg19&lastVirtModeType=default&lastVirtModeExtraState=&virtModeType=default&virtMode=0&nonVirtPosition=&position=chr5%3A142166429%2D146478058&hgsid=3734162217_weUvclH9g2i2v1dMBhrSt6UJG4zV
The tracks from top to bottom:
AS62: DMSO-CTCF-R1
AS66: TSA-CTCF-R1
AS63: DMSO-RAD21-R1
AS67: TSA-RAD21-R1
AS168: DMSO-H3K9Ac-R4
AS171: TSA-H3K9Ac-R4

Methodology

Replicates

Two biological replicates for ChIPseq analysis using antibody CTCF, RAD21, and H3K9Ac.

Sequencing depth	For each sample, we obtained unique reads and mapped reads as summarized in TagInfor.txt and listed as below with the format: Sample Condition Unique positions Total tags. Unique positions refers to uniquely mapped reads while total tags = total mapped reads /2.<br>Replicate 1:<br>AS61:DMSO-IgG-R1: 28353354, 22337160.5<br>AS62:DMSO-CTCF-R1: 43083532, 31055889<br>AS63:DMSO-RAD21-R1: 34099945, 23665699<br>AS65:TSA-IgG-R1: 43109520, 28170759.5<br>AS66:TSA-CTCF-R1: 32826924, 22871705<br>AS67:TSA-RAD21-R1: 37945218, 24749548.5<br>AS167:DMSO-IgG-R4: 44920582, 27595072.5<br>AS168:DMSO-H3K9Ac-R4: 44596559, 29578939<br><br>Replicate 2:<br>AS69:DMSO-IgG-R2: 18841767, 13671844.5<br>AS70:DMSO-CTCF-R2: 20288749, 13882739<br>AS71:DMSO-RAD21-R2: 25096022, 16976237.5<br>AS73:TSA-IgG-R2: 17716741, 12118752<br>AS74:TSA-CTCF-R2: 28020734, 19140419<br>AS75:TSA-RAD21-R2: 26643435, 17432405<br>AS170:TSA-IgG-R4: 45038185, 26624226.5<br>AS171:TSA-H3K9Ac-R4: 45211713, 29269901
Antibodies	CTCF (CST #3418L, lot#6).<br>RAD21 (Abcam ab992, lot#GR3310168-15, recognizing C-terminus)<br>H3K9Ac (Abcam ab4441, lot#1069505-3)<br>IgG: IgG from rabbit serum (Sigma I5006, lot #SLCB7084).
Peak calling parameters	Mapping: bowtie2 -x <directory to> hg19 -p 10 -k 5 -N 1 -1 Sample_R1.fastq.gz -2 Sample fastq.gz -S sample.sam<br>Processing: makeTagDirectory Sample/ Sample.sam -format sam (with default in HOMER)<br>findPeaks Sample/ -style factor -o auto -i Sample-IgG (Using IgG as control and default settings: FDR <0.001; Against IgG control, Fold change over IgG = 4, Poisson p-value over IgG = 0.0001)
Data quality	ChIPseq data quality was examined using HOMER software. Approximate IP efficiency in peak calling report (>1%) is another key factor to evaluate ChIP quality.<br>Peaks were called against IgG of each condition and the HOMER default settings for peak calling were used (FDR < 0.001, fold changes against IgG =4), peak numbers for each library was listed as below:<br>Replicate 1:<br>AS62: DMSO-CTCF-R1: 44670<br>AS63: DMSO-RAD21-R1: 11290<br>AS66: TSA-CTCF-R1: 42582<br>AS67: TSA-RAD21-R1: 7235<br>AS168: DMSO-H3K9Ac-R4: 20839<br>AS171: TSA-H3K9Ac-R4: 34248<br><br>Replicate 2:<br>AS70: DMSO-CTCF-R2: 37007<br>AS71: DMSO-RAD21-R2: 9718<br>AS74: TSA-CTCF-R2: 37255<br>AS75: TSA-RAD21-R2: 8818<br>AS162: DMSO-H3K9Ac-R3: 21462<br>AS165: TSA-H3K9Ac-R3: 33082
Software	For mapping, we used Bowtie2 (Langmead et al., Genome Biology 2009) and mapped onto hg19 human genome reference; For peak calling and data processing, we used HOMER 4.6 (Heinz et al. Mol Cell 2010) and Deeptools 3.0.2 (Ramirez et al. Nucleic Acids Research 2016).

Flow Cytometry

Plots

Confirm that:

- [x] The axis labels state the marker and fluorochrome used (e.g. CD4-FITC).
- [x] The axis scales are clearly visible. Include numbers along axes only for bottom left plot of group (a 'group' is an analysis of identical markers).
- [x] All plots are contour plots with outliers or pseudocolor plots.
- [ ] A numerical value for number of cells or percentage (with statistics) is provided.

Methodology

Sample preparation	For cell cycle analysis, around 1million HAP1-RAD21-TEV cells with DMSO or TSA treatment (TSA 500nM, 3 and 6 hours as indicated) were fixed using 90% ethanol in -20C for at least 24 hours. Fixed cells were washed in 1xPBS and then resuspended in PBS containing 2mM MgCl2, 0.5mg/mL RNase (Roche, 10109169001), 50 ug/mL propidium iodide. The samples were incubated at 20°C for 30min before analysis.<br>For sorting and collecting G1 cells, around 20 million HCT116-RAD21-mAC degron 2 nonsynchronous cells treated with 1) DMSO for 6 hours; 2) 5'-Ph-IAA (1uM) for 6 hours, 3) DMSO for 6 hours, TSA (500nM) for 3 hours; 4) 5'-Ph-IAA (1uM) for 6 hours, TSA (500nM) for 3 hours were fixed using 1% FA for 10mins, then quenched with 135mM glycine (final concentrations) for 5 mins then on ice for 15 mins. Fixed cells were washed in 1xPBS and then resuspended in PBS containing 2mM MgCl2, 0.1% Saponin, 0.5mg/ml RNase A, 50 ug/mL propidium iodide. The samples were incubated at 20°C for 30min before sorting.
Instrument	Cell cycle analysis was performed on BD Symphony A5 and G1 sorting was performed on BD Symphony S6.
Software	Data was collected using software for each machine. Data was analyzed using FlowJo v10
Cell population abundance	The relevant cell population includes all "live" gated cells which was >90% of each sample.
Gating strategy	Preliminary FSC/SSC gates were used to define the population of "live" cells which represented >90% of each sample. Live cells were then represented on a histogram showing propidium iodide intercalaction. Gates for G1, S, and G2/M populations were determined on the nonsynchronous sample within each data set.

☐ Tick this box to confirm that a figure exemplifying the gating strategy is provided in the Supplementary Information.
