# Supplementary figures and images for: Histone Acetylation Differentially Modulates CTCF-CTCF Loops and Intra-TAD Interactions
Source: Nat Commun. 2026 Jul 20;17:6676. doi: 10.1038/s41467-026-75818-8 (PMC13385825; doi:10.1038/s41467-026-75818-8)

Unprocessed blots for Fig. 3a

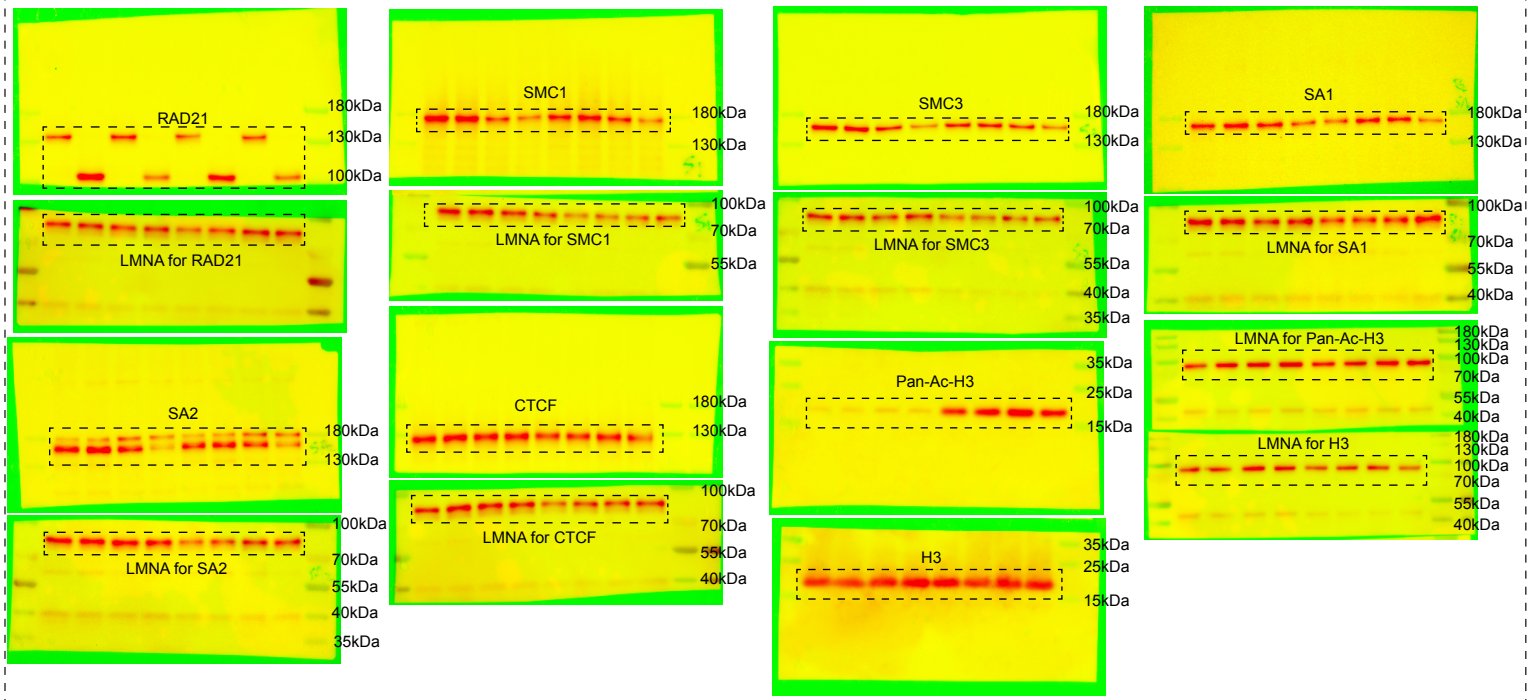

Supplement: Supplementary file 4 — Source Data [file 41467_2026_75818_MOESM4_ESM.zip › 641221_2_data_set_12720975_tm7f9b.pdf]
