# Supplementary material for: Histone Acetylation Differentially Modulates CTCF-CTCF Loops and Intra-TAD Interactions
Source: Nat Commun. 2026 Jul 20;17:6676. doi: 10.1038/s41467-026-75818-8 (PMC13385825; doi:10.1038/s41467-026-75818-8)
Supplement: Supplementary file 3 — Transparent Peer Review file [file 41467_2026_75818_MOESM3_ESM.pdf]

nature portfolio

Peer Review File

Histone Acetylation Differentially Modulates CTCF-CTCF Loops and Intra-TAD Interactions

Corresponding Author: Dr Yu Liu

This file contains all reviewer reports in order by version, followed by all author rebuttals in order by version.

Version 0:

Reviewer comments:

Reviewer #1

(Remarks to the Author)
In this manuscript, Smith and colleagues report a role for histone hyperacetylation in regulation of a subset of cohesin-mediated chromatin interactions. Histone hyperacetylation, induced by relatively short (3 hour) Triochstatin A treatment, and cohesin cleavage (in HAP1-RAD21TEV cells) were employed to characterize two functionally and biochemically distinct pools of cohesin molecules inside cells. The first pool of cohesin molecules are actively engaged in DNA loop extrusion, are non-topologically bound to chromatin, and are sensitive to TSA treatment (and thus hyperacetylation of the chromatin fiber). The second pool of cohesin molecules anchored at CTCF-CTCF loops are topologically engaged with DNA (lost to a greater extent after rapid RAD21 cleavage) and are resistant to TSA-mediated hyperacetylation of chromatin. While these results provide important insights into the various modes by which distinct populations of cohesin molecules engage with chromatin templates during loop extrusion versus during stable cohesin binding at CTCF-CTCF loops or non-CTCF anchored loops, the molecular mechanism(s) remains unclear.

Overall, this is a well-designed, challenging study that addresses an important biological question about how chromosome structure is linked to the state of the underlying chromatin. The manuscript contains excellent quality data, relatively short drug treatments to minimize indirect effects, and compelling conclusions. Unfortunately, there are many problems with data presentation related to incorrect figure callouts that make it difficult for this reviewer to find the data supporting the authors' claims. Nevertheless, the authors should be able to address all issues related to data presentation and the work will be of interest to the field of chromatin biology, transcriptional regulation and epigenetics. Some suggestions are made to enhance the data presentation/interpretation and provide a deeper connection between histone hyperacetylation and cohesin binding and loop formation.

Major comments:
1. Histone hyperacetylation. Trichostatin A is an HDAC inhibitor employed in this study to induce to hyperacetylation. The authors shows that different doses and times of TSA treatment lead to hyperacetylation with a Pan AcH3 antibody. The increase is around 2-fold, according to the quantification in Extended Data Fig 1. It would be useful to show which individual histone residues acquire increased acetylation upon TSA treatment. Is this mediated by H3K27ac? H3K9ac? Other H3 tail residues? Additional western blots should be done and if possible additional ChIP-seq. If additional ChIP-seq is not possible, the authors should explain how histone hyper acetylation may impact how cohesin is engaged with DNA (topo vs non-topo)? Is there increased histone acetylation at the anchors of loops/cohesin binding sites? Or all over chromatin? Knowledge of the extent to which individual residues display altered acetylation upon TSA treatment would help understand potential molecular mechanisms that underlie the authors' observations.
o The authors speculate that extruding cohesin molecules are sensitive to the altered surface charge of chromatin during histone hyperacetylation. Therefore, any additional information about the locations and extent to which chromatin is altered by histone acetylation is important for understanding possible mechanism.

2. Numerous incorrect figure call outs make it difficult to review and find the data that support the authors' claims. A few of the most challenging instances are:
• On pages 9 and 10, there are many references to Extended Data Figure 5 appear to be meant for Extended Data Figure 4. In many of these same sentences, references to Figure 2b appear to be meant for Figure 2c. Also, missing a reference to Figure 2g.
• On page 12 there are numerous references to Extended Figure 8, which does not seem to exist.

• On page 12, “We further quantified these changes by subtracting the loop lines in low salt buffer without RAD21 cleavage to generate relative loop-lines (Fig. 3i and Extended Data Fig. 8i).” Figure 3i is a model and Extend Figure 8i does not exist as a figure or figure legend.
The authors should carefully check and correct all figure call outs.

3. The methodological details of the genomics data analysis are not sufficiently described for others to replicate the results. Please add additional detail and explanation. For example, the ChIP-seq experiments were performed in duplicate, however it does not appear that the data were ever merged and presented in a way that utilizes the variation between duplicates of a condition versus another condition. Or perhaps the data was analyzed this way, but it is not explained. For example, were the sites that showed decreased RAD21 upon TSA treatment in REPLICATE 1 also showing decreased RAD21 signal up on TSA treatment in REPLICATE 2? Or were they different? Was a statistical test used to identify ChIP-seq binding sites that changed significantly? This speaks to the robustness of the decrease in RAD21 signal upon TSA treatment and this point applies broadly to all ChIP-seq data in the study.
• Related, on page 10 the authors state “We next ranked loop anchors by degree of H3K9Ac increases”… was this based on the H3K9Ac signal from a single replicate or a merge of replicates for the TSA treatment condition (and relative to the DMSO condition rep1, rep2 , or a merge of both reps) for the plot in Fig 2e?
• The Hi-C data from each replicate also appear to be kept separate for all analyses, and the authors should explain whether or not replicates are ever merged and also the extent to which changes detected in one replicate (vs control) are also detected in other replicates.

4. Missing unprocessed western blot images. The author guidelines state “All life science papers published in Nature Portfolio journals require submission of unprocessed original images of gels and western blots to be submitted with the final accepted version. These unprocessed images are published in the Supplementary Information.” While the authors state in their “Statistics and Reproducibility Statement” that all western blots were performed at least three times… often only two replicates (not three replicates) are shown and only a single replicate is shown for Figure 1A.

5. What is the biological significance of a pool of cohesin molecules being sensitive to histone hyperacetylation? Do the authors think that hyperacetylation occurs normally and is relevant for the behavior of cohesin molecules inside of cells? How might cohesin molecules be differentially sensitive to hyperacetylation versus normal strong acetylation at active enhancers/promoters? The authors should comment on how their finding about TSA-sensitive cohesin molecules could be relevant for understanding the behaviors and functions of cohesins on the genome under physiological conditions.

Minor comments
1. Need to add a statistic in the figure, text, or figure legend to support this statement “as expected, eliminating cohesin significantly reduces intra-TAD interactions (Fig. 1n and Extended Data Fig. 1o, two replicates, the first and third blue violin plots in each replicate).
2. Figure 2g and Extended Data Figure 4g would benefit from additional labeling in the figure panel and legend to explain what the white-to-brown signal represents.
3. Western blots are missing molecular weight markers.
4. Numerous typos/grammar issues. Just a few are listed here:
• On page 7. “To determine whether loops generated by cohesin that is actively extruding ohesin…”
• On page 7. “Histone hyperacetylation reduces nucleosome interactions and decompact”.
• On page 7. “As cohesin loss eliminate TAD structures,”

Reviewer #2

(Remarks to the Author)
In this study, Smith et al. investigate the influence of chromatin context, specifically hyperacetylation induced by the HDAC inhibitor TSA, on cohesin-mediated loop extrusion and genome organization. They report that TSA treatment alters Eigenvector (EV1) distribution and attenuates TAD strength by reducing intra-TAD contacts, while notably preserving compartment strength and CTCF-anchored loop peaks. A key conclusion is the proposed existence of two cohesin populations: TSA-sensitive, non-CTCF-anchored cohesin and TSA-resistant, boundary-associated cohesin. Finally, they find that TSA sensitizes CTCF loops to cohesin cleavage. While the study addresses a significant question in the field, the data interpretation and evidence supporting the central claims require substantial strengthening.

Major Points:
1. The resolution of the presented Hi-C contact maps is insufficient to robustly evaluate the claimed structural changes. This issue of low data quality also applies to the provided ChIP-seq profiles and Western blots.

2. In Fig. 1c and 1d, the observed global shift in EV1 values following TSA treatment is expected. However, the conclusion that A-A and B-B compartmentalization strength remains unperturbed is surprising and appears to contradict previous findings (e.g., Rosencrance et al. Mol Cell. 2020), which reported that hyperacetylation can induce A-A micro-compartments. The authors should: a. Provide saddle plot analysis to quantitatively assess the interaction strength within A and B compartments. b. Include high-resolution contact maps of representative genomic loci, particularly those exhibiting

significant EV1 shifts, to visually support the claims regarding compartmentalization.

3. The proposed causal relationship between the loss of intra-TAD contacts and the loss of insulation is not justified. These two observations are correlative and could both be parallel consequences of a single upstream effect, such as altered cohesin extrusion dynamics.

4. The experiment in Fig. 1n, which uses cohesin-deficient cells to argue for cohesin's direct involvement, is indirect and has several caveats: a. Acute cohesin loss itself could cause cell cycle redistribution within the 3-hour treatment window, introducing a confounding variable. b. Quantifying cohesin's contribution by a direct comparison of the effect size (0.12 vs. 0.24) is an oversimplification and not biologically meaningful. c. Direct evidence is lacking. To firmly establish the impact, techniques such as cohesin Hi-ChIP should be employed to specifically examine cohesin-mediated contacts.

5. In Fig. 2c, the claim that CTCF binding is unchanged is not fully supported without spike-in controls. The lack of such controls makes it difficult to rule out the possibility of a global attenuation of ChIP signals, a caveat that also applies to the Rad21 data.

6. Fig. 2d shows a reduction in cohesin at CTCF binding sites, yet the authors claim CTCF-CTCF loops are largely unaffected. These two findings appear contradictory and require reconciliation, as stable loop structures would presumably require cohesin retention at boundaries.

7. Is the observed reduction in cohesin a global phenomenon, or does it differ between cohesin peaks at CTCF sites versus those co-localized with cis-regulatory elements (e.g., promoters/enhancers), which may represent cohesin loading sites?

8. The cohesin quantification in Fig. 2a was performed on isolated nuclei, which does not distinguish between nucleoplasmic and chromatin-bound pools. A chromatin fractionation experiment is needed to determine if TSA treatment specifically reduces chromatin-associated cohesin.

9. A decrease in RAD21 ChIP-seq signal does not directly indicate "reduced chromatin association." ChIP-seq measures relative enrichment at specific genomic regions and cannot assess global chromatin binding levels.

10. Numerous figures are mis-referenced throughout the manuscript, particularly those related to Figure 3.

11. The finding that TSA treatment sensitizes CTCF loops to Rad21 cleavage is interesting. What potential underlying mechanism(s) do the authors propose for this observed sensitization?

Minor Points:

1. For Western blot analysis, Histone H3 is a more appropriate loading control than Lamin A for these experiments.

2. Several sentences are confusing. For example, the statement "Hi-C maps revealed that TSA treatment did not cause significant alterations in compartment boundaries or compartment interaction strength, consistent with the Hi-C analysis in cells" is ambiguous. It is unclear which "cells" are being referred to for comparison.

3. Describing the combined effect of TSA and TEV protease as "synergistic" is premature without formal statistical testing for synergy. The effect could simply be additive, and the language should be revised accordingly.

Version 1:

Reviewer comments:

Reviewer #1

(Remarks to the Author)
The authors have sufficiently addressed my concerns by 1) addressing problems with data presentation/interpretation and 2) providing a deeper connection between histone hyperacetylation and cohesin binding and loop formation with a) new data, b) new analyses, and c) new explanations in the text.

Reviewer #2

(Remarks to the Author)
The authors fully addressed all of my comments.

I do not have further comments.

Open Access This Peer Review File is licensed under a Creative Commons Attribution 4.0 International License, which permits use, sharing, adaptation, distribution and reproduction in any medium or format, as long as you give appropriate credit to the original author(s) and the source, provide a link to the Creative Commons license, and indicate if changes were made.
In cases where reviewers are anonymous, credit should be given to 'Anonymous Referee' and the source.
The images or other third party material in this Peer Review File are included in the article’s Creative Commons license, unless indicated otherwise in a credit line to the material. If material is not included in the article’s Creative Commons license and your intended use is not permitted by statutory regulation or exceeds the permitted use, you will need to obtain permission directly from the copyright holder.
To view a copy of this license, visit https://creativecommons.org/licenses/by/4.0/

RESPONSE TO REVIEWERS' COMMENTS

We were very pleased that both reviewers found our work to be of significant interest and recognized its contribution to understanding the relationship between chromosome organization and epigenetic states. We are also grateful for the reviewers' thoughtful and constructive comments, which prompted substantial additional experimental and analytical work to further strengthen the manuscript. We have now fully addressed all reviewer comments by performing extensive new experiments, generating large-scale genomic datasets, and implementing new analytical frameworks. All original conclusions remain fully supported, and we believe that these revisions and additions have significantly strengthened the rigor, depth, and clarity of the study.

Specifically, in response to the reviewers' requests, we added the following new data and analyses:

1. We performed two biological replicates of western blot experiments to systematically assess acetylation changes at representative lysine residues across histones H2A, H2B, H3, and H4.
2. We added two independent ChIP-seq experiments to evaluate the sensitivity and limitations of ChIP-seq in detecting global changes in histone acetylation.
3. We performed two additional ATAC-seq experiments to characterize genome-wide changes in chromatin accessibility and DNA-histone interactions following histone hyperacetylation.
4. We included two biological replicates to quantitatively assess changes in cohesin-chromatin affinity upon chromatin hyperacetylation.
5. We added two independent Hi-C replicates in TSA-treated HCT116 cells, demonstrating that histone hyperacetylation-induced changes in compartment strength are cell-type specific.
6. We performed two additional Hi-C replicates in CTCF-degron cells, which, together with the RAD21 G1-Hi-C data, enabled us to more accurately interpret boundary pile-up signals.
7. To directly address the reviewers' request for cohesin-centered interaction analyses, we performed three additional replicates of ChIP-Loop and one replicate of PLAC-seq targeting RAD21 to measure cohesin-mediated chromatin interactions within TADs upon histone hyperacetylation. This assay is similar with Hi-ChIP.

REVIEWER COMMENTS

Reviewer #1 (Remarks to the Author):

In this manuscript, Smith and colleagues report a role for histone hyperacetylation in regulation of a subset of cohesin-mediated chromatin interactions. Histone hyperacetylation, induced by relatively short (3 hour) Triochstatin A treatment, and cohesin cleavage (in HAP1-RAD21TEV cells) were employed to characterize two functionally and biochemically distinct pools of cohesin molecules inside cells. The first pool of cohesin molecules are actively engaged in DNA loop extrusion, are non-topologically bound to chromatin, and are sensitive to TSA treatment (and thus hyperacetylation of the chromatin fiber). The second pool of cohesin molecules anchored at CTCF-CTCF loops are topologically engaged with DNA (lost to a greater extent after rapid RAD21 cleavage) and are resistant to TSA-mediated hyperacetylation of chromatin. While these results provide important insights into the various modes by which distinct populations of cohesin molecules engage with chromatin templates during loop extrusion versus during stable cohesin binding at CTCF-CTCF loops or non-CTCF anchored loops, the molecular mechanism(s) remains unclear.

Overall, this is a well-designed, challenging study that addresses an important biological question about how chromosome structure is linked to the state of the underlying chromatin. The manuscript contains excellent quality data, relatively short drug treatments to minimize indirect effects, and compelling conclusions. Unfortunately, there are many problems with data presentation related to incorrect figure callouts that make it difficult for this reviewer to find the data supporting the authors' claims. Nevertheless, the authors should be able to address all issues related to data presentation and the work will be of interest to the field of chromatin biology, transcriptional regulation and epigenetics. Some suggestions are made to enhance the data presentation/interpretation and provide a deeper connection between histone hyperacetylation and cohesin binding and loop formation.

We appreciate the reviewers' supportive comments recognizing the relevance and significance of our work. We apologize for any inconvenience caused by unclear or inconsistent data presentation. In the revised manuscript, we have improved data visualization and organization in accordance with the reviewers' suggestions. We sincerely thank the reviewers for their valuable feedback, which has helped us strengthen the clarity and impact of our study. Our detailed responses to each comment are provided below.

Major comments:
1. Histone hyperacetylation. Trichostatin A is an HDAC inhibitor employed in this study to induce to hyperacetylation. The authors shows that different doses and times of TSA treatment lead to hyperacetylation with a Pan AcH3 antibody. The increase is around 2-fold, according to the quantification in Extended Data Fig 1. It would be useful to show which individual histone residues acquire increased acetylation upon TSA treatment. Is this mediated by H3K27ac? H3K9ac? Other H3 tail residues? Additional western blots should be done and if possible additional ChIP-seq.

We thank the reviewer for this helpful suggestion. In the revised manuscript, we have included two biological replicates of Western blot analyses examining acetylation of **H2AK5ac, H2BK5ac, H3K9ac, H3K27ac, H4K5ac, and H4K8ac** following TSA treatment at three concentrations (5, 50, and 500 nM for 3 hours; **Supplementary Fig. 1a**). In addition, we included one replicate of ChIP-seq targeting H3K27ac with and without TSA treatment (**Supplementary Fig. 1b**).

We further examined acetylation changes at RAD21 peaks and found that H3K27ac signal changes upon TSA treatment exhibited a pattern similar to that observed for H3K9ac, supporting the use of H3K9ac as a representative acetylation mark. Together, these results demonstrate that multiple representative lysine residues are robustly acetylated upon 50 or 500 nM TSA treatment, and that these marks show a stronger response to TSA than signals detected using a pan-H3-acetylation antibody.

Because pan-H3-acetylation antibodies recognize multiple acetylated lysine residues on H3 including K9, K14, K18, K23 and K27, they inherently exhibit higher background signal, resulting in more limited apparent fold changes. Consistent with this, when we performed ChIP-seq using a pan-H3-acetylation antibody, we found that it did not effectively capture acetylation signal genome-wide after TSA treatment (**Supplementary Fig. 1c)**. We thus think that TSA-induced global histone hyperacetylation may reduce the detection sensitivity of pan-H3-acetylation antibodies, thereby limiting their utility in this context.

In summary, our new Western blot and ChIP-seq data support the conclusion that TSA, as a pan-HDAC inhibitor, induces robust acetylation across multiple major histone lysine residues at the concentrations used in this study. We agree with the reviewer that mapping additional acetylation sites would further enhance our understanding of histone acetylation dynamics following TSA treatment.

If additional ChIP-seq is not possible, the authors should explain how histone hyper acetylation may impact how cohesin is engaged with DNA (topo vs non-topo)?

We thank the reviewer for this thoughtful comment. Based on our Hi-C and Western blot results, we found that histone hyperacetylation impairs chromatin interactions mediated by extrusive cohesin, while having minimal impact on cohesin-mediated loop formation at CTCF-CTCF sites.

Using our semi-in vitro assay in combination with Hi-C, ChIP-seq, and biochemical readouts, we examined how TSA-induced hyperacetylation affects cohesin-chromatin engagement at CTCF-CTCF sites. Our results support two key conclusions. First, cohesin at CTCF-CTCF loop anchors remains TEV-cleavable after TSA treatment, indicating that cohesin continues to maintain a topological mode of engagement with chromatin at these sites after chromatin is hyperacetylated. Second, TSA treatment increases the TEV sensitivity of cohesin-mediated loop interactions at CTCF-CTCF sites, suggesting that hyperacetylation-induced changes in chromatin properties alter inter-chromatin interactions at these loci. We speculate that histone hyperacetylation weakens histone-DNA contacts and reduces interactions between paired chromatin fibers at CTCF-CTCF sites, thereby increasing their susceptibility to TEV cleavage. Importantly, this enhanced TEV sensitivity does not result in a loss of CTCF-CTCF loop interactions, consistent with topologically engaged cohesin maintaining stable loop anchors even in a modified chromatin environment (**Fig. 3i**).

In contrast, extrusive cohesin is not engaged with chromatin in a topological manner in our model. Accordingly, chromatin-chromatin interactions mediated by actively extruding cohesin are more sensitive to global alterations in chromatin properties induced by histone hyperacetylation, even though overall cohesin association with chromatin (as assessed by chromatin fractionation assays, **Supplementary Fig. 1g**) is not markedly changed. Consistent with this, we observed a reduction in chromatin-chromatin interactions mediated by extrusive cohesin within TADs (**Fig. 1l-o, Extended Data Fig.1n-r, and Fig. 3i**).

Is there increased histone acetylation at the anchors of loops/cohesin binding sites? Or all over chromatin? Knowledge of the extent to which individual residues display altered acetylation upon TSA treatment would help understand potential molecular mechanisms that underlie the authors' observations. o The authors speculate that extruding cohesin molecules are sensitive to the altered surface charge of chromatin during histone hyperacetylation. Therefore, any additional information about the locations and extent to which chromatin is altered by histone acetylation is important for understanding possible mechanism.

We thank the reviewer for these valuable comments. In our manuscript, we performed H3K9ac ChIP–seq analysis and observed no clear correlation between H3K9ac signal intensity and either cohesin binding strength or loop interaction frequency (**Fig. 2d-f and Extended Fig. 4c-e**). We believe this likely reflects inherent limitations of ChIP-seq, which preferentially detects regions with strong enrichment and may therefore fail to capture subtle yet widespread increases in histone acetylation that nonetheless influence higher-order chromatin organization.

To further assess whether this observation is specific to H3K9ac, we included an additional ChIP-seq experiment targeting H3K27ac with and without TSA treatment (**Supplementary Fig. 1b**). Notably, the H3K27ac profile exhibits a pattern similar to that of H3K9ac at RAD21 binding sites, with no preferential enrichment correlated with cohesin occupancy or loop interaction strength. These results suggest that TSA-induced hyperacetylation is not selectively localized to cohesin binding sites or loop anchors.

To evaluate the extent of acetylation across individual histone residues, we performed Western blot analyses examining H2AK5ac, H2BK5ac, H3K9ac, H3K27ac, H4K5ac, and H4K8ac following TSA treatment at three concentrations (5, 50, and 500 nM for 3 hours; **Supplementary Fig. 1a**). At 50 and 500 nM TSA, all examined residues exhibited robust increases in acetylation levels, indicating that TSA induces broad, multi-residue histone hyperacetylation rather than selective modification of specific histones or lysines.

Because ChIP-seq has limited ability to capture global chromatin alterations, we further performed ATAC-seq analysis to probe genome-wide changes in chromatin structure induced by histone hyperacetylation (**Supplementary Fig. 1d**). DNA pitch in ATAC-seq reflects the periodic exposure of DNA major grooves to Tn5 transposase when DNA is wrapped around nucleosomes. Following TSA treatment, this DNA pitch signal is lost, consistent with weakened histone-DNA interactions that increase DNA accessibility along the nucleosome, allowing Tn5 to access DNA more uniformly. The disappearance of DNA pitch therefore indicates a global impact of TSA-induced histone hyperacetylation on chromatin organization.

We appreciate the reviewer for pointing out the importance of acetylated chromatin alternations. In addition to our ATAC-seq results, previous work (*Nozaki et al., Molecular Cell, 2017*) demonstrated that TSA-induced histone acetylation globally reduces nucleosome-nucleosome interactions in living cells. Together, these results support a model in which TSA induces widespread chromatin alterations that may affect cohesin-chromatin interactions. However, our studies indicated that the ways cohesin engaged with chromatin influent their interactions: at CTCF-CTCF sites, cohesin engages chromatin in a topological manner, thus stabilizing the acetylated chromatin interactions, whereas within TADs acetylated chromatin interactions mediated by cohesin are reduced.

2. Numerous incorrect figure call outs make it difficult to review and find the data that support the authors' claims. A few of the most challenging instances are:

• On pages 9 and 10, there are many references to Extended Data Figure 5 appear to be meant for Extended Data Figure 4. In many of these same sentences, references to Figure 2b appear to be meant for Figure 2c. Also, missing a reference to Figure 2g.

We apologize for the mislabeled figures in the manuscript and appreciate the reviewer's time and patience in pointing out these errors. We have corrected these mistakes in the revised manuscript.

• On page 12 there are numerous references to Extended Figure 8, which does not seem to exist.

We apologize for the mislabeled figures in the manuscript and appreciate the reviewer's time and patience in pointing out these errors. We have corrected these mistakes in the revised manuscript.

• On page 12, "We further quantified these changes by subtracting the loop lines in low salt buffer without RAD21 cleavage to generate relative loop-lines (Fig. 3i and Extended Data Fig. 8i)." Figure 3i is a model and Extend Figure 8i does not exist as a figure or figure legend. The authors should carefully check and correct all figure call outs.

We apologize for the mislabeled figures in the manuscript and appreciate the reviewer's time and patience in pointing out these errors. We have corrected these mistakes in the revised manuscript.

3. The methodological details of the genomics data analysis are not sufficiently described for others to replicate the results. Please add additional detail and explanation. For example, the ChIP-seq experiments were performed in duplicate, however it does not appear that the data were ever merged and presented in a way that utilizes the variation between duplicates of a condition versus another condition. Or perhaps the data was analyzed this way, but it is not explained. For example, were the sites that showed decreased RAD21 upon TSA treatment in REPLICATE 1 also showing decreased RAD21 signal up on TSA treatment in REPLICATE 2? Or were they different?

We thank the reviewer for raising this important point. For all omics datasets in this study, we initially analyzed biological replicates independently, as we consider each replicate to represent an independent experiment, and merging replicates can in some cases obscure biological variability and complicate interpretation. We have now clarified and expanded the description of our analysis strategy in the revised Methods section to ensure full transparency and reproducibility.

We fully agree with the reviewer that replicate concordance is critical. In response to this comment, we explicitly compared RAD21 ChIP-seq signals between the two biological replicates. As shown in **Supplementary Fig. 1f**, 7,488 RAD21 peaks were shared between the two DMSO-treated replicates. Importantly, among these common peaks, ~85% showed an ~80% reduction in RAD21 ChIP-seq signal upon TSA treatment (defined as a fold change of TSA versus DMSO < 0.2) in both replicates (**Supplementary Fig. 1f**).

These results demonstrate strong reproducibility between biological replicates and further support our conclusion that TSA treatment leads to a robust and reproducible reduction in RAD21 ChIP-seq signal. We have incorporated these analyses and their interpretation into the revised manuscript.

Was a statistical test used to identify ChIP-seq binding sites that changed significantly? This speaks to the robustness of the decrease in RAD21 signal upon TSA treatment and this point applies broadly to all ChIP-seq data in the study.

We thank the reviewer for pointing this out. Rather than applying a statistical test to define significantly changing ChIP-seq binding sites, we examined the distribution of fold changes for both RAD21 and CTCF signals, as shown in **Supplementary Fig. 1e**. TSA treatment causes a notable reduction in cohesin (RAD21) signals, with the peak of the fold-change distribution close to 0.5, whereas a more modest reduction is observed for CTCF signals, with the peak close to ~0.8. Importantly, both biological replicates show consistent patterns in these fold-change distributions, supporting the robustness of the observed effects.

• Related, on page 10 the authors state “We next ranked loop anchors by degree of H3K9Ac increases”… was this based on the H3K9Ac signal from a single replicate or a merge of replicates for the TSA treatment condition (and relative to the DMSO condition rep1, rep2 , or a merge of both reps) for the plot in Fig 2e?

We thank the reviewer for this comment. For this correlation analysis, we used the H3K9ac signal from a single biological replicate rather than a merged dataset. We apologize that this was not stated clearly in the original manuscript. Following the reviewer’s suggestion, we have clarified this in the **Fig. 2 and Extended Data Fig. 4** legends in the revised manuscript. Specifically, the analysis shown in **Fig. 2e and Extended Data Fig. 4** were performed relative to the corresponding DMSO control from the same biological replicate (Replicate 1 for **Fig. 2e** and Replicate 2 for **Extended Data Fig. 4**).

• The Hi-C data from each replicate also appear to be kept separate for all analyses, and the authors should explain whether or not replicates are ever merged and also the extent to which changes detected in one replicate (vs control) are also detected in other replicates.

We thank the reviewer for this comment. All Hi-C analyses were performed using independent biological replicates, and replicates were never merged for any analyses. Changes induced by TSA treatment were assessed separately in each replicate, and the same qualitative and quantitative trends were consistently observed across replicates, as illustrated in the manuscript. We have clarified this point in the revised section of Statistics and Reproducibility.

4. Missing unprocessed western blot images. The author guidelines state “All life science papers published in Nature Portfolio journals require submission of unprocessed original images of gels and western blots to be submitted with the final accepted version. These unprocessed images are published in the Supplementary Information.” While the authors state in their “Statistics and Reproducibility Statement” that all western blots were performed at least three times… often only two replicates (not three replicates) are shown and only a single replicate is shown for Figure 1A.

We thank the reviewer for pointing this out. In the revised manuscript, we have included all unprocessed Western blot images, including all new Western analysis for this revision, in the Supplementary Information **(Supplementary Fig. 5-7)**, in accordance with Nature Portfolio author guidelines, and corrected the Statistics and Reproducibility Statement accordingly.

We originally performed two biological replicates for the Western blot experiments; however, the manuscript incorrectly stated that at least three replicates were performed. This has now been corrected. For the key semi-in vitro assay, we performed an additional biological replicate (the third replicate) and included these data in **Supplementary Fig. 1h**. Consistent with the other two replicates, this additional experiment shows no change in cohesin ring integrity upon TSA-induced hyperacetylation.

5. What is the biological significance of a pool of cohesin molecules being sensitive to histone hyperacetylation? Do the authors think that hyperacetylation occurs normally and is relevant for the behavior of cohesin molecules inside of cells? How might cohesin molecules be differentially sensitive to hyperacetylation versus normal strong acetylation at active enhancers/promoters? The authors should comment on how their finding about TSA-sensitive cohesin molecules could be relevant for understanding the behaviors and functions of cohesins on the genome under physiological conditions.

We thank the reviewer for highlighting the biological relevance of our work. A key conclusion of this study is that cohesin engages chromatin through distinct physical modes that differentially determine its sensitivity to histone acetylation.

Within TAD interiors, extruding cohesin within TAD is highly dynamic and chromatin is not threaded through cohesin rings, cohesin-mediated looping interactions in these regions are therefore highly sensitive to local chromatin state. Note that while loops mediated by this form of extruding cohesin are TSA sensitive, chromatin binding by cohesin is not (See **Supplementary Fig. 1g**). This suggests that cohesin has at least two interaction surfaces with chromatin that are both needed to hold the two anchors of a chromatin loop, and that chromatin binding of only one of these is TSA sensitive.

We appreciate the reviewer's insightful comment and have revised the Discussion accordingly to better address this point (Page 15):

Extruding cohesin acts through a dynamic cycle of transient chromatin interactions. For example, the SMC-RAD21 interface initially clamps chromatin, followed by projection of the SMC1-SMC3 hinge that promotes movement along the chromatin fiber. These interacting surfaces may be susceptible to changes in chromatin charge and conformation. Histone hyperacetylation alters chromatin surface charge, which appears to result in impaired extrusion activity and weakened intra-TAD interactions, whereas chromatin binding by cohesin is largely unaffected (Supplementary Fig. 1g). This suggests that cohesin has at least two interaction surfaces with chromatin that are both needed to hold the two anchors of a chromatin loop, and that chromatin binding of only one of these is TSA sensitive.

By contrast, cohesin anchored at convergent CTCF-CTCF sites topologically encircles chromatin, with DNA passing through the cohesin ring. This topological configuration stabilizes cohesin-chromatin interactions and renders the loops they mediate substantially more resistant to global changes in chromatin acetylation.

Importantly, chromatin states differ systematically across genomic contexts under physiological conditions. At transcription start sites and associated regulatory elements, enhancer-promoter interactions are mediated by transcription factors and Mediator, and chromatin is typically hyperacetylated and more accessible. Our previous work demonstrated that cohesin-mediated interactions at these regions are particularly sensitive to cohesin ring opening, suggesting that cohesin engages chromatin at these sites in a topological manner. In the context of the present findings, this mode of engagement is well suited to support enhancer-promoter communication, providing both structural stability and regulatory plasticity.

Together, these observations suggest that differential sensitivity of cohesin to histone acetylation reflects the diverse physical modes by which cohesin interacts with chromatin across the genome, and that modulation of chromatin acetylation may contribute to fine-tuning cohesin function under

physiological conditions. These concepts have been incorporated into the Discussion of the revised manuscript and are further illustrated with a schematic cartoon highlighting their functional consequences (**Fig. 4g**).

Minor comments

1. Need to add a statistic in the figure, text, or figure legend to support this statement “as expected, eliminating cohesin significantly reduces intra-TAD interactions (Fig. 1n and Extended Data Fig. 1o, two replicates, the first and third blue violin plots in each replicate).

We thank the reviewer for this comment. We performed statistical analysis using the Wilcoxon rank-sum test, which yielded a p value < 0.001. This statistical result has now been included in the figure legend in the revised manuscript.

2. Figure 2g and Extended Data Figure 4g would benefit from additional labeling in the figure panel and legend to explain what the white-to-brown signal represents.

We thank the reviewer for this comment. The white-to-brown signal represents relative contact frequency normalized by cis-expected interactions. We have added additional labeling in both the figure panels and figure legends to clarify this in the revised manuscript.

3. Western blots are missing molecular weight markers.

We thank the reviewer for pointing this out. Following the reviewer’s comment, we have added molecular weight markers to all Western blot panels in the revised manuscript.

4. Numerous typos/grammar issues. Just a few are listed here:
• On page 7. “To determine whether loops generated by cohesin that is actively extruding ohesin…”
• On page 7. “Histone hyperacetylation reduces nucleosome interactions and decompact”.
• On page 7. “As cohesin loss eliminate TAD structures,”

We thank the reviewer for pointing out these typographical and grammatical errors. We have carefully corrected these issues throughout the manuscript in the revised version. Examples include the following corrections:

“To determine whether loops generated by actively extruding cohesin, by cohesin stalled at pairs of CTCF sites, or by both mechanisms are affected, we next examined the impact on each loop type individually.”

“Histone hyperacetylation reduces nucleosome interactions and decompacts chromatin fibers...”

“As cohesin loss eliminates TAD structures, …”

Reviewer #2 (Remarks to the Author):

In this study, Smith et al. investigate the influence of chromatin context, specifically hyperacetylation induced by the HDAC inhibitor TSA, on cohesin-mediated loop extrusion and genome organization. They report that TSA treatment alters Eigenvector (EV1) distribution and

attenuates TAD strength by reducing intra-TAD contacts, while notably preserving compartment strength and CTCF-anchored loop peaks. A key conclusion is the proposed existence of two cohesin populations: TSA-sensitive, non-CTCF-anchored cohesin and TSA-resistant, boundary-associated cohesin. Finally, they find that TSA sensitizes CTCF loops to cohesin cleavage. While the study addresses a significant question in the field, the data interpretation and evidence supporting the central claims require substantial strengthening.

Major Points:
1. The resolution of the presented Hi-C contact maps is insufficient to robustly evaluate the claimed structural changes. This issue of low data quality also applies to the provided ChIP-seq profiles and Western blots.

We thank the reviewer for this comment. We believe that the apparent reduction in figure quality likely resulted from PDF compression applied to meet the file size requirements for submission. For each Hi-C experiment, we generated more than 150 million paired-end reads, and for each ChIP-seq experiment, more than 30 million paired-end reads. All Hi-C contact maps and Western blot images were originally exported at 600 dpi.

We apologize for any inconvenience caused by this compression and have now included representative panels with minimal compression to improve clarity. In addition, for Hi-C contact maps derived from cell lines, we increased the sequencing depth by more than 200 million reads per sample. As a result, local contact changes are now more clearly resolved in **Supplementary Fig. 2**, which also addresses the second point raised by Reviewer #2.

2. In Fig. 1c and 1d, the observed global shift in EV1 values following TSA treatment is expected. However, the conclusion that A-A and B-B compartmentalization strength remains unperturbed is surprising and appears to contradict previous findings (e.g., *Rosencrance et al. Mol Cell. 2020*), which reported that hyperacetylation can induce A-A micro-compartments. The authors should:
a. Provide saddle plot analysis to quantitatively assess the interaction strength within A and B compartments. b. Include high-resolution contact maps of representative genomic loci, particularly those exhibiting significant EV1 shifts, to visually support the claims regarding compartmentalization.

We thank the reviewer for this very valuable comment. Previous work has indeed identified A-A micro-compartments formed by the oncoprotein BRD4-NUT (*Rosencrance et al., Mol Cell, 2020*). In our study, we used TSA to transiently inhibit HDAC activity and induce global histone hyperacetylation. Interestingly, although we observed an increase in the number of A-compartment bins (EV1>0, **Fig.1c and Extended Data Fig. 1f**), we did not detect a notable increase in global A-A interaction strength (**Fig. 1d and Extended Data Fig.1g**).

The reviewer raises an important point, as the formation of A-A micro-compartments in the BRD4-NUT system might suggest that hyperacetylation could enhance A-A interactions. <u>However, our observations indicate that such effects are not universal and may depend on cellular context.</u> To explore this further, we reanalyzed the published Hi-C datasets from *Rosencrance et al. Mol Cell, 2020*), in which global A-A and B-B compartment interaction strengths were not explicitly quantified. Although overexpression of BRD4-NUT in HEK293 cells leads to the appearance and strengthening of A-A micro-compartments, global A-A and B-B compartment interaction strengths remain largely unchanged (**Supplementary Fig. 3b**). In contrast, in TC-797 cells, MZ1 treatment reduces A-A micro-compartments and is accompanied by a modest reduction in global A-A

compartment strength (**Supplementary Fig. 3c**). Notably, in both systems, overall compartment strength changes are limited (**Supplementary Fig. 3b-c**).

These analyses suggest that the impact of histone hyperacetylation on compartmentalization strength is cell-type dependent, consistent with the well-established cell-type specificity of A/B compartment organization. To further validate this observation, we performed additional Hi-C analyses in HCT-116 cells (**Supplementary Fig. 3d)**. In this context, we observed a notable reduction in A-A compartment interactions, accompanied by a mild decrease in B-B compartment interactions. Together, these results indicate that histone hyperacetylation can have cell-type-specific effects on compartment interactions, which may manifest as micro-compartment formation or modest global changes depending on cellular context.

While these findings are intriguing, the primary focus of our study is on cohesin-mediated loop extrusion and intra-TAD organization. A detailed investigation of compartment reorganization under hyperacetylation conditions is therefore beyond the scope of the present study.

a. We thank the reviewer for this helpful suggestion. We have now included saddle plot analyses in **Supplementary Fig. 3a**, which quantitatively assess A-A and B-B interaction strengths and show that overall compartment strength changes are minimal, consistent with our conclusions.

b. We thank the reviewer for this valuable suggestion. We identified two representative genomic regions exhibiting EV1 shifts and present the corresponding Hi-C contact maps in **Supplementary Fig. 2a-b**. We further increased the sequencing depth for each sample by at least 200 million reads and generated higher-resolution contact maps for these loci (**Supplementary Fig. 2c-d**). Importantly, increased sequencing depth did not alter the EV1 profiles for the same loci within the same samples.

Although EV1 profiles showed notable alternations, we did not observe corresponding changes in the underlying contact maps. To further assess potential differences, we generated differential contact heatmaps ($\log_2$[TSA/DMSO]) after observed/expected normalization using the deeper sequencing data (**Supplementary Fig. 2e-f**). These analyses similarly revealed no notable changes in contact patterns.

Taken together, these results indicate that TSA-induced histone hyperacetylation has a limited impact on chromatin compartmentalization, despite detectable changes in EV1 profiles.

3. The proposed causal relationship between the loss of intra-TAD contacts and the loss of insulation is not justified. These two observations are correlative and could both be parallel consequences of a single upstream effect, such as altered cohesin extrusion dynamics.

We thank the reviewer for this thoughtful and insightful comment. We would like to clarify that, in the original submission, we did not intend to establish a causal relationship between the loss of intra-TAD contacts and the reduction in insulation strength. In Page 7, we used the shoulder peaks in the TAD boundary pile-up plots to illustrate changes in intra-TAD interactions; however, we recognize that this presentation may have been misleading, and we apologize for the confusion.

Importantly, this comment raises an interesting and important question: how should reductions in insulation strength be interpreted, and how are these changes related to loop extrusion activity? We address this point below.

First, we revisited the interpretation of the TAD boundary pile-up plots. As shown in **Supplementary Fig. 4a**, the shoulder regions capture contacts within TADs and at TAD edges. These signals peak due to structural features such as CTCF-anchored loops and stripes, as illustrated in **Supplementary Fig. 4a** (blue dashed box as the sliding window to calculate insulation scores), which encompasses stripes, loops, and intra-TAD contacts. Therefore, a reduction in shoulder peaks reflects combined changes in intra-TAD interactions, stripes, and looping interactions. Given that CTCF-CTCF loops are largely unaffected by TSA treatment, the observed reduction in shoulder peaks upon TSA treatment likely reflects reduced intra-TAD and stripe interactions, rather than loss of looping per se.

In contrast, the valley (insulation depth) in the pile-up plots reflects inter-TAD interactions, as the insulation diamond (red dashed box in **Supplementary Fig. 4a**) specifically captures contacts between neighboring TADs. The valley itself does not reflect CTCF-CTCF looping interactions. Notably, a shallower valley corresponds to increased inter-TAD interactions. Thus, TSA-induced reductions in intra-TAD interactions are accompanied by increased inter-TAD contacts, manifesting as reduced insulation strength.

To further examine this relationship, we generated and analyzed Hi-C data from both RAD21 and CTCF degron cell lines (**Supplementary Fig. 4b**). Because acute RAD21 depletion induces G2/M arrest, all RAD21-depletion Hi-C analyses were performed on **G1-sorted cells**. Upon RAD21 depletion, shoulder peaks were nearly abolished, consistent with the loss of both intra-TAD interactions and CTCF-CTCF looping. Similarly, CTCF depletion led to a reduction in shoulder peaks, reflecting loss of CTCF-anchored loops. Strikingly, depletion of either RAD21 or CTCF resulted in increased inter-TAD interactions, indicating a concomitant loss of TAD boundary insulation.

Together, we think that disruption of cohesin-mediated loop dynamics leads to decreased intra-TAD organization and CTCF-CTCF looping interactions, while increased inter-TAD contacts, resulting in reduced insulation strength. In this sense, we agree with the reviewer that decreased intra-TAD interactions and increased inter-TAD interactions are both consequences of impaired cohesin-dependent loop extrusion dynamics.

To correct the misleading interpretation in the original submission, we have revised the manuscript text as follows (Page 7):

We next quantified changes in intra-TAD interactions by calculating the average interaction frequency within TADs (see Methods). We observed that TSA treatment significantly reduced intra-TAD interactions across replicates. Notably, we also detected a reduction in TAD boundary insulation strength. Rather than implying a causal relationship, we interpret this reduction as a consequence of how insulation scores are calculated: decrease in intra-TAD interactions result in an apparent weakening of boundary insulation. (Fig. 1l and Extended Data Fig. 1n).

4. The experiment in Fig. 1n, which uses cohesin-deficient cells to argue for cohesin's direct involvement, is indirect and has several caveats: a. Acute cohesin loss itself could cause cell cycle redistribution within the 3-hour treatment window, introducing a confounding variable. b. Quantifying cohesin's contribution by a direct comparison of the effect size (0.12 vs. 0.24) is an oversimplification and not biologically meaningful. c. Direct evidence is lacking. To firmly establish the impact, techniques such as cohesin Hi-ChIP should be employed to specifically examine cohesin-mediated contacts.

We thank the reviewer for these insightful comments. Below we provide detailed responses to each point.

a. Potential cell-cycle effects following acute cohesin depletion

We appreciate the reviewer's recognition of potential confounding effects caused by acute cohesin depletion. To minimize cell-cycle–related variability, in our original manuscript we performed Hi-C analysis on G1-sorted cells following acute RAD21 degron induction (**Fig. 1m-o and Extended Data Fig. 1o-r**), as in our previous studies (*Liu and Dekker, NCB, 2022*). This approach effectively avoids confounding effects arising from cell-cycle redistribution during the 3-hour treatment window.

b. Interpretation of effect size comparisons

We agree that comparing effect sizes alone (e.g., 0.12 vs. 0.24) is an oversimplification and may not be biologically meaningful. For this reason, in the original manuscript we additionally analyzed scaling plots, which report interaction frequencies as a function of genomic distance. As shown in **Fig. 1o and Extended Fig. 1p**, intra-TAD interactions are consistently reduced across a broad range of genomic separations following cohesin depletion, providing a more robust and quantitative assessment of the weakening of local chromatin contacts.

c. Direct evidence for cohesin-mediated contacts

We thank the reviewer for this very valuable suggestion. To directly assess cohesin-mediated contacts, we initially performed RAD21 PLAC-seq experiments and generated five paired RAD21 PLAC-seq libraries (DMSO versus TSA), totaling over one billion sequencing reads. However, despite this substantial sequencing depth, we encountered technical limitations in DNA recovery, and only one PLAC-seq library reached acceptable quality for downstream analysis.

In parallel, we implemented the ChIP-loop assay (*Kim and Dekker, 2018, Cold Spring Harbor Protocols*), which captures cohesin-mediated interactions from 3C libraries rather than Hi-C-derived libraries, as used in HiChIP or PLAC-seq. Three independent ChIP-loop libraries exhibited high data quality, enabling robust measurement of cohesin-mediated interactions within TADs. Notably, intra-TAD interaction analyses from all three ChIP-loop libraries, as well as from the single high-quality PLAC-seq library, consistently showed reduced interactions within TADs following TSA treatment (**Extended Data Fig. 1s-r** and **Supplementary Fig. 3e-g**), in agreement with our Hi-C results. Derivative plots further revealed a reduction in short-range interactions, while CTCF-CTCF loops remained largely unchanged. Since both RAD21 ChIP-loop and PLAC-seq specifically capture cohesin-mediated chromatin interactions, these results provide direct evidence that histone hyperacetylation impacts on cohesin-mediated interactions, fully consistent with our Hi-C analysis.

We appreciate the reviewer's insightful comments and agree that this validation experiment is critical for our study. Accordingly, we included two biological replicates in Extended Data Fig. 1s-r and described the results in the main text as outlined below (Page 8).

To further validate that cohesin-mediated interactions are altered by TSA-induced histone hyperacetylation, we performed RAD21-targeted ChIP-loop and PLAC-seq, both of which specifically capture and enrich cohesin-mediated chromatin interactions (See Methods). These analyses revealed a marked reduction in cohesin loop signals following TSA treatment. Notably, TSA-induced histone hyperacetylation led to decreased intra-TAD interactions while having minimal impact on CTCF–CTCF loop interactions, consistent with our observations from Hi-C analysis (Extended Data Fig. 1s-t, two replicates ChIP-Loop, Supplementary Fig. 3e, another replicate of ChIP, Supplementary Fig. 3f. one replicate of PLAC-seq).

We did not identify statistically significant loop calls using the FitHiChIP pipeline. Nevertheless, recognizing the importance of directly interrogating cohesin-mediated loop contacts, we pursued an alternative integrative strategy (**Supplementary Fig. 4c**).

Specifically, we identified 7,780 high-confidence RAD21 peaks that were consistently detected in both RAD21 ChIP-seq replicates under DMSO treatment. These peaks mapped to 7,488 20-kb genomic bins, yielding approximately 1.3 million candidate loop pairs. After restricting the analysis to genomic distances between 100 kb and 1 Mb, we obtained 22,315 candidate pairs, of which 1,184 did not overlap with annotated CTCF-CTCF loops. Pile-up analysis of Hi-C contact frequencies at these sites revealed no notable changes upon TSA treatment, similar with the minimal effects of TSA on CTCF-CTCF loops. These results indicate that TSA has limited impact on cohesin-mediated loop interactions when loop anchors are marked by strong cohesin enrichment, as detected by RAD21-based assays.

Together, these results strongly support our conclusion that TSA-induced histone hyperacetylation primarily affects cohesin extrusion dynamics rather than stable, anchor-defined loop interactions. We agree that imaging-based approaches could provide additional direct evidence, and such experiments represent an important direction for future investigation.

5. In Fig. 2c, the claim that CTCF binding is unchanged is not fully supported without spike-in controls. The lack of such controls makes it difficult to rule out the possibility of a global attenuation of ChIP signals, a caveat that also applies to the Rad21 data.

We thank the reviewer for these valuable comments. We agree that, in principle, spike-in ChIP-seq can help assess global changes in ChIP signal; however, its interpretation is subject to several well-recognized limitations. In particular, spike-in normalization assumes comparable antibody efficiency and chromatin accessibility between experimental samples and spike-in material, which is often difficult to achieve in practice. For factors such as cohesin, whose binding is locus-specific and context-dependent, spike-in approaches can be especially sensitive to technical variability, differences in chromatin composition, and non-uniform effects across the genome.

We attempted to perform spike-in ChIP-seq; however, these experiments were unsuccessful, largely due to the complexity and variability of spike-in signals. Similar concerns regarding the interpretation of ChIP-seq signals were also raised by Reviewer #1, and we address it in detail in our response to **Question 3 from Reviewer #1 (Supplementary Fig. 1e)**.

6. Fig. 2d shows a reduction in cohesin at CTCF binding sites, yet the authors claim CTCF-CTCF loops are largely unaffected. These two findings appear contradictory and require reconciliation, as stable loop structures would presumably require cohesin retention at boundaries.

We thank the reviewer for these valuable comments. We have explained this in the discussion section as highlighted below (Page 15).

"Interestingly, despite preserved CTCF-CTCF loops, we observe a marked reduction in cohesin ChIP-seq signal at CTCF loop anchor sites after TSA treatment. This uncoupling between cohesin occupancy and loop interactions suggests the presence of two cohesin populations at loop anchors. We propose that a TSA-sensitive population-likely consisting of newly loaded or transiently extruding, non-encircling cohesin-is preferentially depleted following chromatin hyperacetylation, whereas a TSA-resistant population-composed of topologically bound cohesin-maintains loop interactions. Supporting this model, RAD21 cleavage in TSA-treated nuclei

abolishes CTCF-CTCF loops, confirming that maintenance of this type of loops depends on the TSA-resistant, encircling form of cohesin (Fig. 3f-h).

The TSA-sensitive population may reflect cohesin in the process of transitioning between dynamic and stable binding modes. We propose two non-mutually exclusive scenarios. First, at a given loop anchor, CTCF may engage in multiple loop interactions across cells: some stabilized by topologically-bound cohesin holding two CTCF sites, and others formed via non-encircling complexes with distal non-anchor CTCF sites. Consistent with this, we showed in our previous work that only CTCF-CTCF loops are mediated by the topologically-bound form of cohesin, whereas loops where only one anchor is at a CTCF sites are held by the extruding non-topological form18. In this scenario, loss of the latter population upon TSA treatment would reduce cohesin ChIP signal, loss of the CTCF-nonCTCF loops, but will not alter aggregate CTCF-CTCF loop strength. Alternatively, paused extruding cohesin may transiently accumulate at CTCF-bound anchors already occupied by stable topologically-bound cohesin. TSA-induced depletion of the extruding population would again reduce cohesin ChIP signal, while preserving the underlying loop that are maintained by the stably bound cohesin complex. Both scenarios account for reduced cohesin occupancy with CTCF-CTCF loop maintenance and highlight the biochemical heterogeneity of cohesin at loop bases."

7. Is the observed reduction in cohesin a global phenomenon, or does it differ between cohesin peaks at CTCF sites versus those co-localized with cis-regulatory elements (e.g., promoters/enhancers), which may represent cohesin loading sites?

We thank the reviewer for this insightful comment. In response, we examined the impact of histone acetylation on RAD21 binding at active promoters that either do or do not bind CTCF (**Supplementary Fig. 4d**). We found that RAD21 ChIP-seq signals at active transcription start sites are sensitive to TSA treatment regardless of whether a nearby CTCF site is present (within 2 kb). Across the two biological replicates, cohesin signals show some variability, with RAD21 ChIP-seq signals in replicate I exhibiting less sensitivity to TSA treatment than those in replicate II; nevertheless, a subset of cohesin binding sites remains resistant to TSA treatment. In contrast, CTCF ChIP-seq signals are relatively resistant to TSA treatment and show higher consistency across replicates.

These observations are consistent with the dynamic nature of cohesin binding and suggest distinct modes of cohesin engagement at active transcription start sites. Active TSSs with and without nearby CTCF sites were defined as described in *Liu and Dekker, NCB, 2022*.

8. The cohesin quantification in Fig. 2a was performed on isolated nuclei, which does not distinguish between nucleoplasmic and chromatin-bound pools. A chromatin fractionation experiment is needed to determine if TSA treatment specifically reduces chromatin-associated cohesin.

We thank the reviewer for this very valuable comment. We agree that ChIP-seq does not report global levels of chromatin-bound proteins, and that our semi-in vitro assays using isolated nuclei cannot, on their own, distinguish between nucleoplasmic and chromatin-associated pools.

To directly address this concern, we performed chromatin fractionation experiments following the approach described by Abramo et al. (NCB, 2019). As shown in **Supplementary Fig. 1g**, TSA-induced histone hyperacetylation did not result in detectable changes in the distribution of cohesin

between nucleoplasmic and chromatin-bound fractions. These results indicate that histone hyperacetylation has minimal impact on the overall chromatin affinity of cohesin.

This observation supports our conclusion that histone hyperacetylation disrupts cohesin-mediated loop interactions within TADs without impairing cohesin's ability to associate with chromatin. During loop extrusion, cohesin simultaneously engages two regions of the chromatin fiber. Our data suggest that histone hyperacetylation impairs cohesin engagement with one of these chromatin anchors while maintaining overall chromatin binding. However, based on the current results, we cannot yet determine which specific anchor interaction is selectively compromised by histone hyperacetylation. We have added discussion of this model in the revised manuscript.

9. A decrease in RAD21 ChIP-seq signal does not directly indicate "reduced chromatin association." ChIP-seq measures relative enrichment at specific genomic regions and cannot assess global chromatin binding levels.

We thank the reviewer to point this out. We have revised this statement accordingly: "Further, RAD21 ChIP-seq signals also showed a general decrease in cohesin-enriched sites following histone acetylation".

We addressed global chromatin binding levels of cohesin when we address Question 1 from Reviewer #1 and Question 8 from Reviewer #2. Please refer to **Supplemental Fig. 1g** for the analysis of cohesin chromatin association across two biological replicates following TSA-induced hyperacetylation.

10. Numerous figures are mis-referenced throughout the manuscript, particularly those related to Figure 3.

We apologize for the mislabeled figures in the manuscript and appreciate the reviewer's time and patience in pointing out these errors. We have corrected these mistakes in the revised manuscript.

11. The finding that TSA treatment sensitizes CTCF loops to Rad21 cleavage is interesting. What potential underlying mechanism(s) do the authors propose for this observed sensitization?

We thank the reviewer for this insightful question. Our new ATAC-seq data (**Supplementary Fig. 1d**) indicate that histone hyperacetylation weakens DNA-histone interactions, resulting in reduced nucleosome-DNA contacts and a more expanded chromatin conformation, consistent with previous observations (*Nozaki et al., Molecular Cell, 2017*). Based on these results, we propose the following mechanism, and we thus revised the model as illustrated in Fig. 3i and the corresponding Discussion section (Page 16).

Histone hyperacetylation reduces nucleosome interactions by altering the surface charge of nucleosomes, thereby weakening chromatin-chromatin interactions between neighboring chromatin regions. At pairs of looping CTCF sites, cohesin topologically holds two chromatin fibers together. As a result, although histone hyperacetylation weakens interactions between the two acetylated chromatin fibers, CTCF-CTCF loop interactions remain largely intact due to cohesin-mediated tethering. Upon RAD21 cleavage, opening of the cohesin ring releases both chromatin fibers. Because acetylated chromatin exhibits weakened inter-fiber interactions, loop contacts at CTCF-CTCF sites become more sensitive to cohesin ring cleavage compared to non-acetylated chromatin. This explains the increased sensitivity of CTCF loops to RAD21 cleavage following TSA treatment (**Fig. 3i, bottom**)

Finally, it is intriguing that while loops mediated by the extruding form of cohesin are sensitive to TSA, chromatin association of cohesin is not. This suggests that cohesin has at least two interaction surfaces with chromatin that are both needed to hold the two anchors of a chromatin loop, and that chromatin binding of only one of these is TSA sensitive.

We appreciated this very insightful comment of the reviewer, based on this important comment, we also revised the corresponding abstract and result sections, highlighted as below:

Abstract:
(Page 2)
Using a semi-in vitro system with TEV-cleavable RAD21, we show that histone hyperacetylation renders CTCF-CTCF loops more sensitive to proteolytic cleavage that opens the cohesin ring. These results reveal that topological engagement of cohesin with DNA underlies the TSA resistance of CTCF-CTCF loops.

Results:
(Page 12)
In our previous studies, we showed that cohesin topologically engages chromatids at CTCF-CTCF loop sites, rendering these loops sensitive to RAD21 cleavage18. Based on our observaction that histone hyperacetylation has minimal impact on CTCF-CTCF loop interactions, we hypothesize that histone hyperacetylation dose not alter the mode by which cohesin engages chromatin at CTCF-CTCF loop sites, thereby preserving loop sensitivity to RAD21 cleavage.

Fig. 3 legend:
(Page 20)
Since chromatin with hyperacetylated histones exhibits weakened inter-chromatin interactions (red arrows), RAD21 cleavage results in a greater loss of loop interactions between pairs of CTCF sites. Dark blue dots indicate chromatin viewed from the top.

Minor Points:

1. For Western blot analysis, Histone H3 is a more appropriate loading control than Lamin A for these experiments.

We thank the reviewer for this helpful comment. Because the total H3 band migrates very close to the acetylated H3 band, we used Lamin as a loading control to quantify acetylated H3 levels on the same blot. This approach allowed us to avoid the need to strip and re-probe the membrane for total H3, thereby preserving signal quality and ensuring accurate quantification.

2. Several sentences are confusing. For example, the statement "Hi-C maps revealed that TSA treatment did not cause significant alterations in compartment boundaries or compartment interaction strength, consistent with the Hi-C analysis in cells" is ambiguous. It is unclear which "cells" are being referred to for comparison.

We thank the reviewer for this helpful comment. This sentence was intended to compare the similarity between nuclear and whole-cell Hi-C results. We apologize for the earlier confusing wording and have revised the sentence as follows (Page 11):

“Hi-C maps generated from purified nuclei revealed that TSA treatment did not cause significant alterations in compartment boundaries or compartment interaction strength, consistent with the Hi-C analysis performed on the intact cells from which the nuclei were purified.”

3. Describing the combined effect of TSA and TEV protease as "synergistic" is premature without formal statistical testing for synergy. The effect could simply be additive, and the language should be revised accordingly.

We thank the reviewer for this very valuable comment. In our revised manuscript, we removed the word of “synergistic” to avoid any misleading.
